# Abstracts from the British Society of Echocardiography annual meeting 2025

**DOI:** 10.1186/s44156-026-00108-4

**Published:** 2026-04-06

**BSEcho 2025 Summary**

It is time to reflect on another successful BSE conference, where we were welcomed to (an unseasonably) sunny Bournemouth in the usually chilly month of October. Across two days, around 700 delegates were present in person and nearly another 700 subsequently participated online to experience the best that echo has to offer.

The programme this year addressed a diverse range of subjects that are relevant to practitioners irrespective of experience. Furthermore, as the first ‘people’s conference’, we had been overwhelmed by suggested sessions and topics from the BSE community at large, and it was wonderful for us to include virtually all submissions in one form or another.

Those delegates attending in person were able to benefit from a variety of workshops that facilitated hands-on and small group learning, supervised simulator training, or could take advantage of vendor-sponsored sessions. Lectures were available to all and covered everything from pulmonary hypertension to physiologist-based practice, aortopathy to amyloid, and congenital to cardiomyopathies.

We were delighted to welcome several keynote speakers, all of whom shared their experience and knowledge with passion and clarity. We thank Professor Simon Ray, Dr Shantanu Sengupta, Dr Carol Whelan, and Professor Denisa Muraru for their contribution and for helping to make this year’s conference truly unforgettable.

Whilst we all value and enjoy the education and learning that conference delivers, what sets the BSE apart is a sense of togetherness and community that is unlike most other societies. What better way to demonstrate that togetherness than celebrating members who have contributed to echocardiography in their own settings up and down the country. On Friday evening we congratulated and recognised new BSE Fellows, aided and abetted by a few complimentary drinks, and suitably raucous applause.

Finally, a personal word from ourselves. It has been an absolute honour and pleasure for us to lead on education and to develop conference over the last three years. We hope that you have enjoyed coming as much as we have enjoyed putting on the show. Our time has now come to an end. We have passed the education baton onto Drs Lynne Williams and Kelly Victor, who will take the BSE forward and we wish them all the best.

Professor David Oxborough and Dr Liam Ring

**ABS001 Discrepancies in self-reported versus measured height and weight and the potential impact on transthoracic echocardiography findings**

Christopher Benson^1^, Richard Graham^1^, David Austin^1,2^, Chris Wilkinson^1,3^

^1^Academic Cardiovascular Unit, The James Cook University Hospital, Middlesbrough, UK, ^2^Population Health Science Institute, Newcastle University, Newcastle upon Tyne, UK, ^3^Hull York Medical School, University of York, York, UK

Published paper: 10.1186/s44156-025-00095-y

*Echo Research & Practice 2026*, **13(Suppl 1):**ABS001

## ABS002 Inappropriate ECHO requests in Irish hospitals

John Peter McCormick^1^, Ellen Beirne^2^, Robert Trueick^3^, Robert Evans^4^

^1^St Luke’s Hospital, Kilkenny, Ireland, ^2^Galway University Hospital, Galway, Ireland, ^3^Connolly Hospital, Blanchardstown, Dublin, Ireland, ^4^Waterford University Hospital, Waterford, Ireland

*Echo Research & Practice 2026*, **13(Suppl 1):**ABS002

### Background

Demand for ECHO has risen dramatically over the past three decades. Appropriate use criteria have been developed to minimize inappropriate ordering of ECHO, which can lead to excessive waiting list times and inefficient resource utilization. Despite this, inappropriate request rates of 10.9%-15.3% have been reported.

### Methods

100 sequential ECHO requests were prospectively analysed in each of three Irish hospitals. Requests were compared against the 2022 British Society of Echocardiography appropriate use criteria. Data relating to both appropriate and inappropriate indications was collected. Reports for ‘inappropriate’ scans were reviewed to determine whether clinically relevant information was obtained.

### Results

Of the 299 requests reviewed, 89 (29.8%) were deemed to be inappropriate or incomplete. Significant pathology was identified in 13 (14.65%) scans performed for inappropriate requests, of which 6 (6.7%) were deemed to be clinically relevant, while 7 (7.9%) represented incidental findings. Arrhythmia, heart failure and syncope in the setting of suspected structural heart disease were the most common ‘appropriate’ indications. ‘Falls workup’, unconfirmed stroke / transient ischemic attack presentations and syncope without evidence of underlying structural heart disease were the most common inappropriate requests.

### Conclusion

Almost a third of ECHO requests were found to be inappropriate or incomplete. The diagnostic yield of these scans was low. Such low-yield investigations likely contribute to excess waiting times and divert resources away from higher value interventions. Improved education and collaboration with general medical, geriatric and stroke services are indicated to improve service efficiency and diagnostic yield.

**Fig. 1(abstract ABS002):**
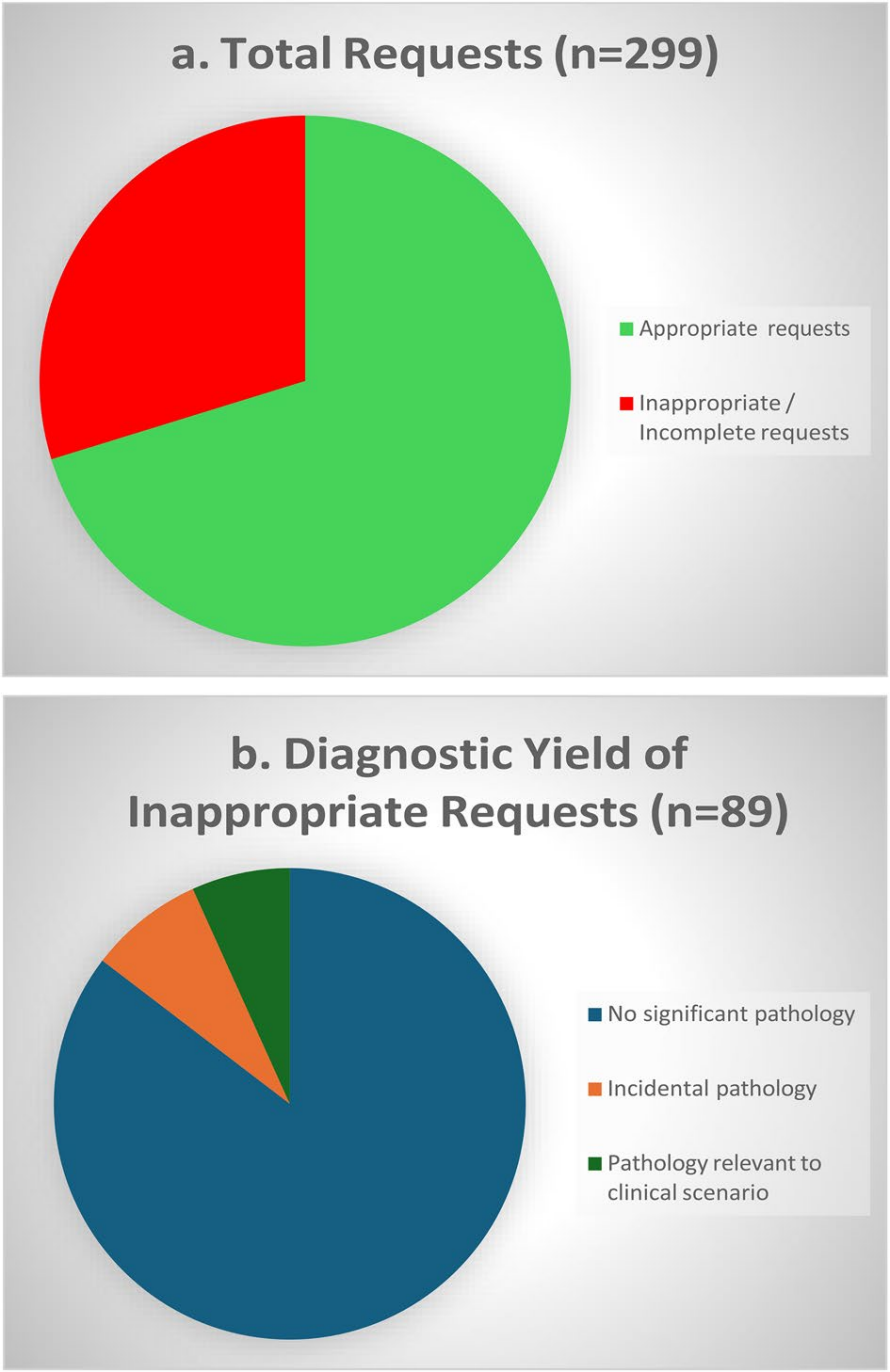
(a) Proportion of inappropriate requests. (b) Diagnostic yield of tests performed without an appropriate indication

**Fig. 2(abstract ABS002):**
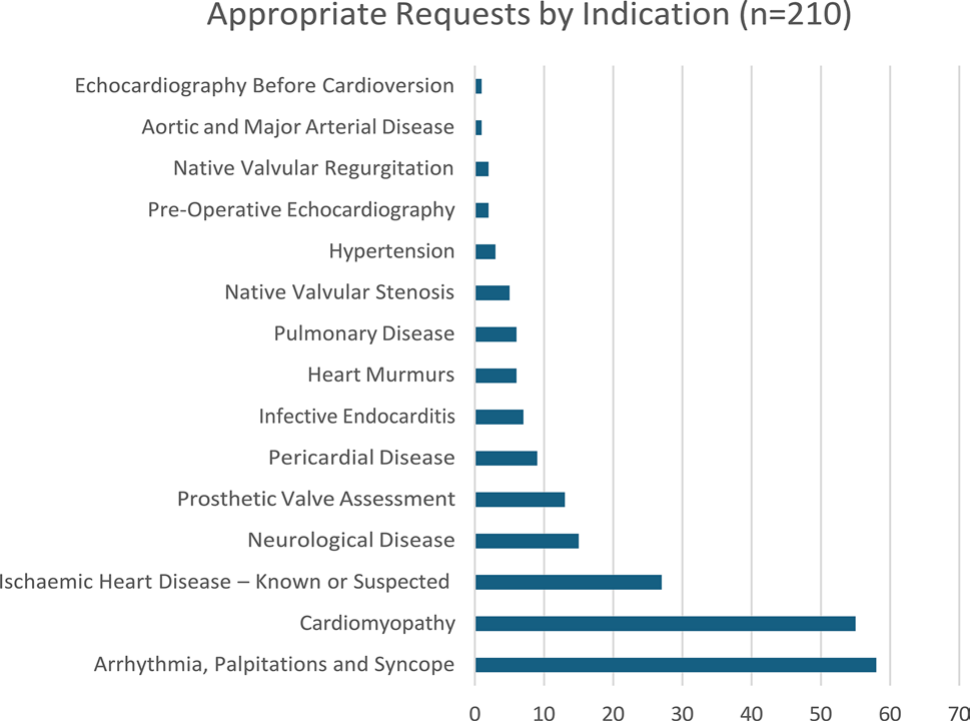
Most common appropriate indications for transthoracic ECHO

**Table 1:** Indications for ECHO

Appropriate Requests (n = 210)		Inappropriate Requests (n = 89)	
Indication	Requests	Indication	Requests
**Arrhythmia, Palpitations and Syncope**	**58**	**Arrhythmia, Palpitations and Syncope**	**32**
Clinical suspicion of structural heart disease in proven arrhythmia	32	‘Falls Workup’ - no further clinical information	17
Syncope in a patient with clinically suspected heart disease	18	‘Collapse’ or ‘Syncope work-up’- no further clinical information	11
Assessment of patients without clinical suspicion of structural heart disease who have an arrhythmia commonly associated with structural heart disease	6	‘Palpitations’ - no further clinical information	3
Post-operative evaluation of patients following RF ablation and surgical procedures in the absence of complications	1	Sinus bradycardia	1
Assessment of ventricular function for secondary prevention of SCD in VT	1		
**Cardiomyopathy**	**55**	**Cardiomyopathy**	**7**
Clinical or radiographic signs of heart failure	23	Breathlessness with alternate explanation or inadequate clinical information	5
Repeat assessment in documented cardiomyopathy with change in clinical status	15	Short interval repeat ECHO without change in clinical status	1
Baseline LV function and periodic review when using cardiotoxic drugs, egherceptin	7	Peripheral oedema - no further clinical infor- mation	1
Suspected cardiomyopathy based on abnormal examination, ECG, or family history in first degree relative	6		
Unexplained shortness of breath in the absence of clinical signs of heart failure if ECG/CXR abnormal	2		
Clinical cardiomegaly	1		
Repeat assessment in documented car- diomyopathy where result may change management or following procedures affecting function, eg cardiac resynchronisation, septal ablation	1		
**Ischaemic Heart Disease – Known or Suspected**	**27**	**Ischaemic Heart Disease – Known or** **Suspected**	**4**
Assessment of infarct size, presence of complications and baseline LV function following MI	15	Non-cardiac chest pain	2
Evaluation of patients with non-diagnostic ECG and indeterminate laboratory markers if performed during or immediately after cardiac chest pain	8	Troponin Leak’ - no further clinical information	1
Evaluation of LV function to guide fur- ther therapy or assess effect of inter-	2	Non-specific ECG changes in setting of acute non-cardiac illness	1
vention, e.g. drug therapy, ICD implantation, CRT, patients scheduled to undergo coronary artery bypass surgery			
Chest pain with haemodynamic instability	1		
Stress echocardiography to assess re- versible ischaemia, myocardial viability and risk stratification	1		
**Neurological Disease**	**15**	**Neurological Disease**	**20**
Unexplained stroke or TIA without evidence of prior cerebrovascular disease or without significant risk factors of other cause	13	Unconfirmed stroke or transient ischemic attack prior to other work-up	13
Acute interruption of blood flow to major peripheral or visceral artery	2	Explained stroke or transient ischaemic attack	7
**Prosthetic Valve Assessment**	**13**		
Baseline assessment of newly implanted prosthetic valve	5		
Repeat assessment of prosthetic valve with change in clinical status	4		
Repeat assessment of prosthetic valve with clinical findings suggestive of dysfunction	4		
**Pericardial Disease**	**9**		
Suspected pericarditis, pericardial effusion, tamponade or constriction	9		
**Infective Endocarditis**	**7**	**Infective Endocarditis**	**7**
To characterise valvular lesions, haemodynamic consequences and ventricular response in a patient with clinically proven or suspected endocarditis	6	High CRP	3
Detection of high-risk complications, eg fistula, abscess, mass lesions	1	Single positive blood culture with atypical or- ganism	3
		Dental abscess without positive blood cultures	1
**Heart Murmurs**	**6**	**Heart Murmurs**	**3**
Murmur in the presence of cardiac or respiratory symptoms	5	Incidental murmur - no further clinical information	3
Murmur in an asymptomatic individual in whom clinical features or other in-vestigation suggest structural heart disease	1		
**Pulmonary Disease**	**6**	**Pulmonary Disease**	**2**
Lung disease with clinical suspicion of cardiac involvement (cor pulmonale)	2	Pulmonary embolism - no further clinical information	1
Suspected or established pulmonary hypertension	2	Diagnosis of Pulmonary Embolism Prior to CT	1
Suspected or established pulmonary embolism to inform a decision regarding thrombolysis	1		
To distinguish cardiac from non-cardiac causes of dyspnoea when the results of clinical and other diagnostic testing are ambiguous	1		
**Native Valvular Stenosis**	**5**		
Repeat assessment of known stenosis with change in clinical status	3		
Periodic repeat assessment of asymptomatic individual with moderate stenosis for valve severity, ventricular size and function	1		
Initial assessment of aetiology and severity, ventricular size and function	1		
**Hypertension**	**3**	**Hypertension**	**1**
Suspected LV dysfunction	2	Hypertension not meeting criteria for ECHO	1
Evaluation of LVH and LV remodelling where this will alter management	1		
**Pre-Operative Echocardiography**	**2**	**Pre-Operative Echocardiography**	**9**
Unexplained shortness of breath in the absence of clinical signs of heart failure if ECG and/or CXR abnormal	2	Pre-Operative request not meeting criteria for ECHO	9
**Native Valvular Regurgitation**	**2**		
Repeat assessment in known regurgitation with change in clinical status	1		
Periodic repeat assessment of asymptomatic individual with known severe re- gurgitation for ventricular size and function	1		
**Aortic and Major Arterial Disease**	**1**		
Repeat assessment of prior surgical repair of aorta	1		
**Echocardiography Before Cardioversion**	**1**		
Guidance for decision to attempt cardioversion, eg LV function, MV disease	1		
		**Miscellaneous**	4
		Acute kidney injury	1
		Low Glasgow Coma Scale	1
		No clinical information	1
		Post surgery ‘baseline’	1

RF = Radiofrequency, SCD = Sudden cardiac death, VT = ventricular tachyardia, LV= left ventricle, CXR = Chest X-Ray, MI = Myocardial Infarction, ICD = Implantable Cardioverter-Defibrillator, CRT= Cardiac resynchronization therapy, TIA = Transient Ischemic Attack, MV = Mitral Valve

**ABS003 Clinical appropriateness of inpatient transthoracic echocardiography referrals when applying the British Society of Echocardiography triaging guidance**

Samuel Walsh^1,2^

^1^Bradford Teaching Hospitals NHS Foundation Trust, UK, ^2^Manchester Metropolitan University, UK

*Echo Research & Practice 2026*, **13(Suppl 1):**ABS003

### Background

Transthoracic echocardiography (TTE) is one of the most utilized imaging modalities. However, the demand for TTE outweighs the ability to supply it, with waiting lists drastically increasing over recent years. Additionally, with the high burden of patients admitted into inpatient care and a decreasing number of available beds, TTE is frequently used within the management of inpatients. To prevent overutilisation of TTE when it is not clinically required, the British Society of Echocardiography have previously published triaging guidance for inpatient TTE in 2022. There is limited research regarding the effectiveness of guidelines within clinical practice, which warrants further investigation.

### Aims

We aim to assess the appropriateness of inpatient TTE referrals at Bradford Royal Infirmary against the BSE triaging guidelines. We also aim to compare clinical findings of TTE against TTE referral appropriateness.

### Methods

A retrospective analysis of inpatient TTE referrals was conducted between 1^st^ November-31^st^ December 2022 at Bradford Royal Infirmary, which identified 475 eligible patients for analysis. The BSE triaging guidelines were used to categorize referrals as ‘appropriate’ or ‘inappropriate’ for the inpatient setting. Qualitative data was used to identify referral reasons, clinical findings, and management of the patient. Quantitative data was used when collecting mean (±SD) data and calculating percentages of referral appropriateness and clinical findings. Comparison of TTE findings with TTE referral appropriateness was conducted using a Chi-squared test.

### Results

The mean age of patients was 63 (± 22) years. 58% of patients were male. The average waiting time for inpatient TTE was 2 days, with 23% of all patients waiting ≥3 days. 21% (*N*=99) of patients were discharged prior to having a TTE. 26% (*N*=124) of inpatient TTE referrals were deemed inappropriate. A lack of relevant clinical information was the main reason for inappropriate TTE. Inappropriate inpatient TTE referrals demonstrated significantly less new abnormal findings (23% vs. 47%, P=<0.001) and abnormal findings requiring further inpatient management (11% vs. 34%, P=<0.001), when compared with appropriate referrals. The 11% (*N*=14) of inappropriate referrals requiring further inpatient management because of TTE findings were all attributed to lack of relevant clinical information on the TTE referral.

### Conclusion

This study highlighted that a vast amount of inpatient TTEs were being inappropriately referred. Fifty-six hours of TTE scanning time could have been saved and utilized in other areas, such as reducing the outpatient TTE waiting lists. This study also highlighted the effectiveness of BSE triaging guidance, particularly in highlighting those with abnormal TTE findings. Further research is warranted into improving TTE referral appropriateness. This could involve educational interventions on BSE triaging guidance or implementing an improved referring system to aid referrers to include all relevant clinical information.

## ABS005 The importance of using the latest BSE guidelines on age-related cut-offs for NT-proBNP on clinical management

Ahmed M. Khalifa^1^, Mostafa Abdulaziz ^1^, Harshavardhani Addada ^1^, Sitara Khan ^1^

^1^Frimley Park Hospital, Surrey, UK

*Echo Research & Practice 2026*, **13(Suppl 1):**ABS005

### Background

In 2024, the BSE released guidance around inpatient echo requesting using age-adjusted NT- proBNP levels.

### Purpose

To evaluate adherence to this BSE guidance.

### Methods

Retrospective review of the clinical notes of 110 inpatients who underwent echocardiography for suspected heart failure, over a three-month period in a Surrey DGH. In addition to NT- proBNP levels, clinical symptoms and signs, investigation results, frailty score, and any change in patient management following the echo were documented.

### Results

The mean patient age was 76.6 (± 12.3) years, 53% were male. While 80% of patients had documented heart failure symptoms/signs, 20% did not. Of the patients with symptoms, 25% did not meet the new BSE NT-proBNP cut-off levels. Severe valve disease was identified in 8. Frailty of a moderate or greater degree, defined by a frailty score of 5-8, was present in 27% of patients within our study. 73% of these frail patients did not have a documented change in their management after the echocardiogram. Overall, a change in patient management was documented in 35% of the entire patient group.

### Conclusion

A significant proportion of echocardiogram requests for “suspected heart failure” were based on high NT-proBNP levels in the absence of clinical heart failure symptoms/signs. Looking at echo requests following appropriately measured NT-proBNP, age-adjusted NT-proBNP was not consulted in a quarter of patients. Over 70% of moderate to severely frail patients had no change in management post-echocardiography, suggesting that the results may have had limited value in the overall management of this subgroup. An impactful change on management, demonstrated as initiation of prognostic medications, device therapy and surgical or procedural interventions, was noted in a third of patients.

## ABS006 Heart valve clinics: An expanding role for the clinical scientists - validation of a framework for competency and certification

Can Zhou^1^, Jerusalem Fekadu^1^, Anna Hayes^1^, Nathalie Aure^1^, Masha Sivalinganathan^1^, Lucy Bowen^1^, Brian Campbell^1^, Sheila Subbiah^1^, Curtis Page^1^, Sophie Bennett^1^, Ronak Rajani^1,2^, Camelia Demetrescu^1^

^1^Cardiology, Guy’s and St Thomas’ Hospitals NHS Trust, London, UK, ^2^Cardiac CT, Guy’s and St Thomas’ Hospitals NHS Trust, London, UK

Published paper: https://openheart.bmj.com/content/openhrt/11/2/e002865.full.pdf

*Echo Research & Practice 2026*, **13(Suppl 1):**ABS006

## ABS007 AI-assisted myocardial infarction diagnosis from echocardiography videos

Aaisha Khan^1^, Iman Islam^1^, Nilanka Mannakkara^2^, Daniela Noakes^2^, Ahmed Mansour^2^, Lia Davies^2^, Cecilia Marcolin^2^, Annelise Aquilina^2^, Federico Dignazi^2^, Samuel Parsons^2^, Andrew King^1^, Thomas Day^1, 2^

^1^School of Biomedical Engineering and Imaging Sciences, King’s College London, UK, ^2^Guy’s and St Thomas’ NHS Foundation Trust, London, UK

*Echo Research & Practice 2026*, **13(Suppl 1):**ABS007

### Background

Echocardiography after myocardial infarction (MI) provides valuable diagnostic information through assessment of regional wall motion abnormalities, but interpretation requires significant expertise and remains subject to intra- and inter-observer variability. Artificial intelligence (AI) has shown promise in the automatic interpretation of echocardiography images.

### Purpose

To implement and evaluate an AI model for MI diagnosis from echocardiographic videos, in terms of stand-alone diagnostic performance and its use as a decision-support tool for clinicians with particular attention to the potential of AI visual explanations for improving clinical trust calibration.

### Methods

We used a ResNet18-LSTM model with an attention mechanism to classify MI versus healthy from apical two-chamber (A2C) and four-chamber (A4C) echocardiographic views. Data included 127 A4C and 120 A2C videos from the publicly available HMC-QU dataset. Thirteen A4C and 12 A2C videos were reserved as independent test sets, with the remainder used for training. Performance metrics included sensitivity, specificity, accuracy, positive predictive value (PPV), negative predictive value (NPV), and area under the ROC curve (AUC), reported with 95% confidence intervals to reflect uncertainty due to the small dataset. Gradient-weighted Class Activation Mapping (Grad-CAM) generated visual explanations. Eight trainee cardiologists evaluated clips under three conditions: (a) original clips, (b) clips with AI prediction, (c) clips with AI prediction and Grad-CAM visual explanations.

### Results

For A2C views, the model achieved sensitivity 1.00 (95% CI 0.65–1.00), specificity 0.40 (0.12–0.77), accuracy 0.75 (0.47–0.91), and AUC 0.943 (0.72–1.00). For A4C, sensitivity was 0.75 (0.41–0.93), specificity 1.00 (0.57–1.00), accuracy 0.85 (0.58–0.96), and AUC 0.925 (0.73–1.00). Combined A2C+A4C results were sensitivity 0.93 (0.69–0.99), specificity 0.91 (0.62–0.98), accuracy 0.92 (0.75–0.98), and AUC 0.95 (0.85–1.00). In the end-user study, AI alone achieved 80.0% accuracy versus 77.0% for clinicians, while combining AI with human judgment did not improve performance. Adding Grad-CAM explanations reduced accuracy to 72% and specificity from 93.8% to 83.8% (*p* = 0.046).

### Conclusion

Our findings suggest that while AI models can effectively detect MI on echocardiographic videos, current explainability techniques may misalign with clinical reasoning, potentially impairing diagnostic performance. Future effective integration of AI into echocardiographic workflows will require not only high accuracy but also AI visual explanation strategies that complement clinician expertise.

**Fig. 1(abstract ABS007):**
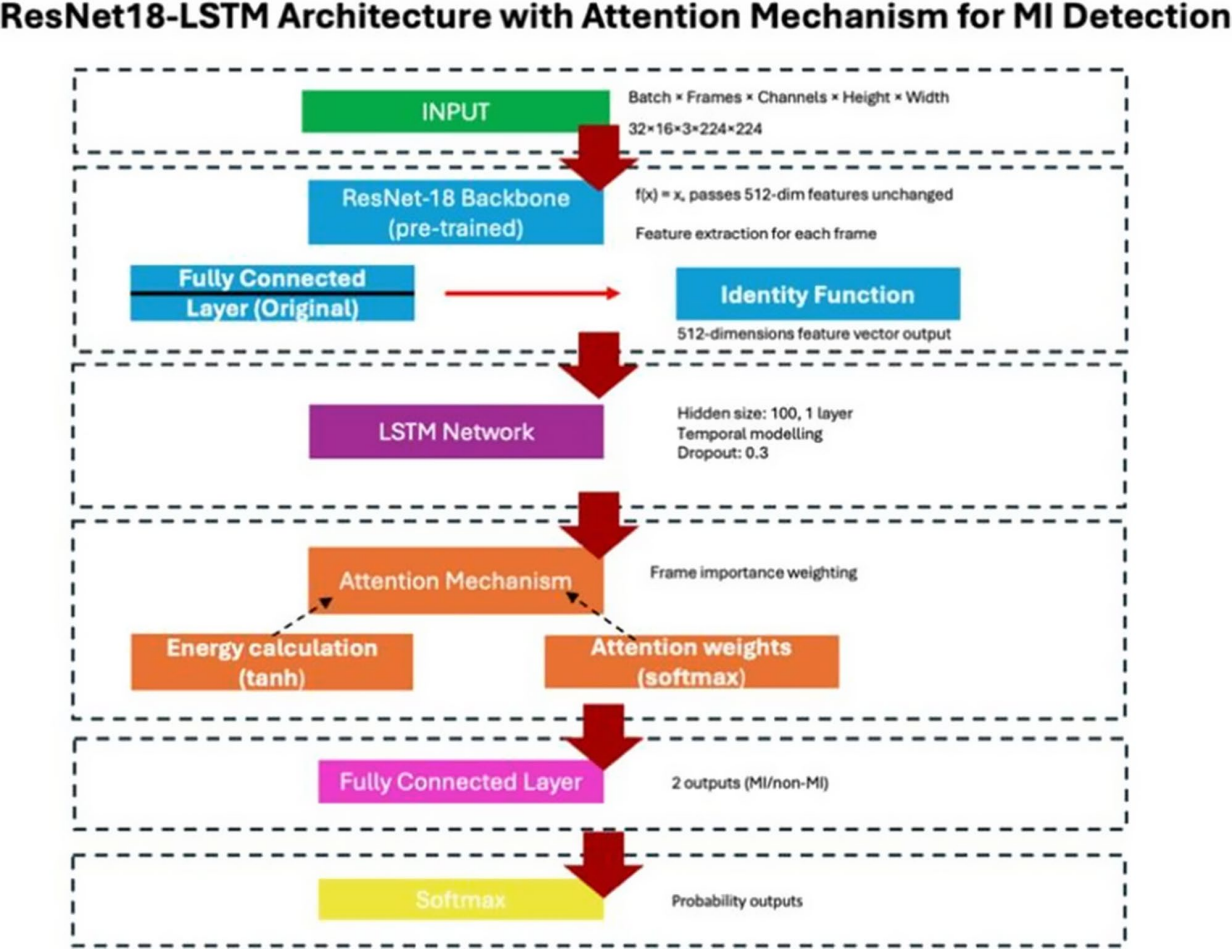
Architecture diagram of the ResNet18-LSTM with attention model

**Fig. 2(abstract ABS007):**
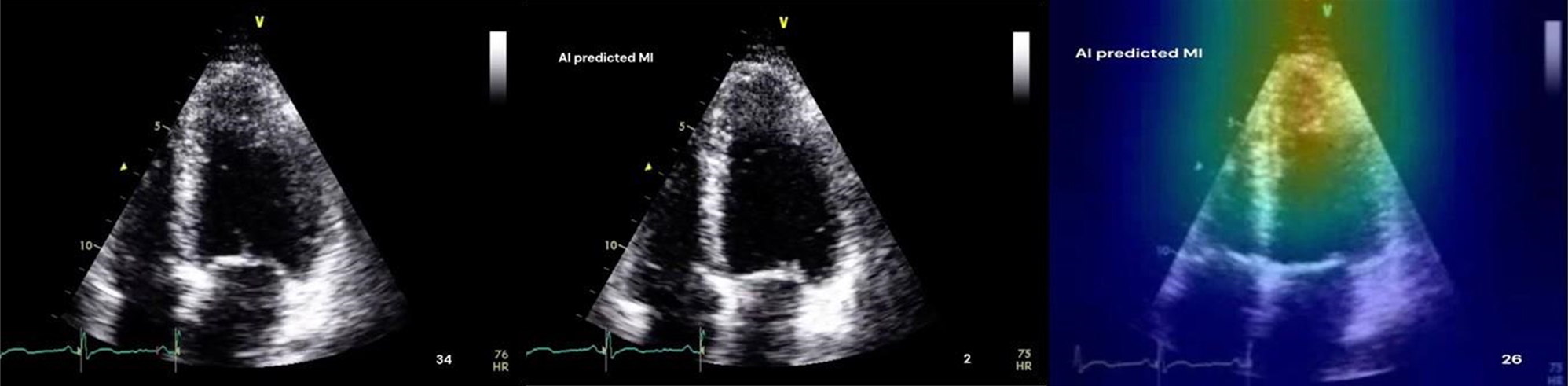
Example of the three randomised viewing conditions for the same A4C echocardiographic clip, as seen by clinicians. **Left:** unaltered echo clip; **middle:** AI model prediction (“MI” or “non-MI”) overlaid; **right:** AI prediction with additional Grad-CAM heatmap highlighting regions important to the model’s decision

**Fig. 3(abstract ABS007):**
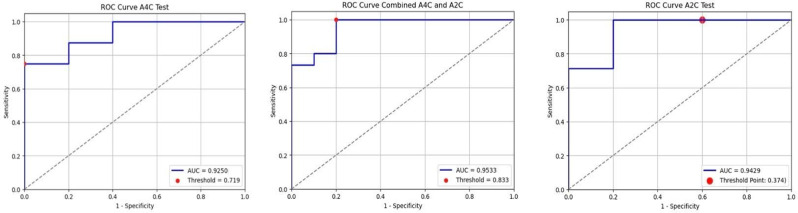
ROC curves showing performance for (left to right): A2C view, combined A2C and A4C, and A4C view. AUC values: 0.9250 (A4C), 0.9533 (Combined), 0.9429 (A2C)

## ABS010 The clinical relevance of incidental findings of aortic valve calcification and aortic dilation identified on lung cancer screening CT scans

Olivia O’Connor^1^, Robin Shome^1^, Sitara Khan^1^

^1^Frimley Health Foundation Trust, UK

*Echo Research & Practice 2026*, **13(Suppl 1):**ABS010

### Background

The Targeted Lung Health Check (TLHC) scheme is a national programme offering CT screening for lung cancer for smokers aged 55-74. Although primarily flagging suspicious lung lesions for investigation, the programme also identifies incidental findings which require careful consideration to avoid unnecessary overtreatment.

### Purpose

We aimed to evaluate the clinical relevance of incidental findings on TLHC CT scans referred to cardiologists by the programme’s Screening Review Meeting (SRM), namely the findings of aortic valve calcification and aortic dilation.

### Methods

We collected data from Epic for 113 patients scanned on the TLHC programme between December 2022 and December 2024 who were referred from the SRM to cardiologists at Frimley Health Foundation Trust.

### Results

54 patients (48%) had at least moderate aortic valve calcification on CT scan, with 30 (56%) identified as moderate and 24 (44%) as severe. 50 (93%) underwent a follow-up echocardiogram. 17% of those with moderate calcification on CT had moderate or severe AS, and 62% of those with severe calcification on CT had moderate or severe AS. 49 patients (43%) had aortic dilation on CT scan. 45 (92%) underwent a follow-up echocardiogram. Of those with mild dilation on CT scan, 7 (41%) had mild aortic regurgitation (AR) on echocardiogram and one (6%) had moderate AR. Of those with moderate dilation on CT scan, 15 (63%) had mild AR on echocardiogram. Of those with severe dilation on CT scan, 3 (75%) had mild AR on echocardiogram. None of the patients with aortic dilation of any severity on CT had severe AR.

### Conclusion

The incidental finding of severe aortic valve calcification on CT scans is clinically significant in nearly two thirds of patients, requiring either ongoing valve surveillance or review in cardiology clinic. Incidental aortic dilation correlates only infrequently with aortic regurgitation that is greater than mild in severity.

## ABS011 Comparing 3D echocardiography with 2D and cardiac magnetic resonance imaging for right ventricular assessment in congenitally caused pulmonary hypertension

James Benstead^1^, Trishla Patel^1^, Rehan Junejo^2^

^1^Manchester Heart Centre, Manchester Royal Infirmary Hospital, UK, ^2^Life Sciences Department, Manchester Metropolitan University, UK

*Echo Research & Practice 2026*, **13(Suppl 1):**ABS011

### Background

Pulmonary hypertension (PH) is common in adult congenital heart disease, frequently associated with septal defects which cause a pressure overload on the right ventricle (RV). The ability of the RV to manage these increased pressures determines patient prognosis. The currently used 2D-echocardiography is limited in comprehensively quantifying RV dysfunction, requiring cardiac magnetic resonance imaging (CMRI) to provide volumetric measures. 3D-echocardiography offers an alternative which encompasses functional and volumetric data obtained via 2D- echocardiography and CMRI respectively. Therefore, 3D-echocardiography has the potential to provide a more comprehensive assessment of RV function and volumetric changes for patients with congenitally caused PH.

### Purpose

Compare the accuracy of 3D-echocardiography against 2D-echocardiography for selective RV assessment measures in congenitally caused pulmonary hypertension patients with septal defects.

### Methods

Retrospectively compared 2D-echocardiography and CMRI RV based measurements against 3D-echocardiography values in a cohort of 30 patients diagnosed with congenitally caused pulmonary hypertension. RV measures assessed included tricuspid annular plane of systolic excursion (TAPSE), fractional area change (FAC), end systolic volume, end diastolic volume and RV ejection fraction. CMRI scans were required to be within 12 months of 2D/3D echocardiographic assessment. Paired samples t-testing assessed statistical significance (*P*<0.05) between methods of measurement, with Bland Altman plots demonstrating mean levels of agreement.

### Results

A statistically significant underestimation was reported for all 3D volumetric measures in comparison to CMRI volumetric measurements (Table 2). However, there was no significant difference between 3D and 2D functional RV measures (TAPSE, FAC) as well as CMRI measurement of RV ejection fraction, suggesting strong levels of agreement (Table 3).

### Conclusion

For this patient cohort, 3D-echo has proven successful in reproducing RV functional measures; however, it significantly underestimated all volumetric measures in comparison to CMRI. However, this underestimation was proportional, allowing for accurate RV ejection fraction calculation which provides important diagnostic information helping clinicians manage congenitally caused PH patients.

**Table 2:** Means and standard deviations for each 3D, 2D and CMRI measure, with P values

Measure	3D mean (S.D)	2D mean (S.D)	P values
Fractional Area Change (%)	36.2 (10.69)	35.73 (8.76)	0.417
Tricuspid Annular Plane Systolic Excursion (cm)	2.12 (0.52)	2.08 (0.51)	0.086
		**CMRI mean (S.D)**	
RV ejection fraction (%)	47.44 (6.69)	47.49 (7.04)	0.94
End Diastolic Volume (ml)	174.36 (54.85)	184.63 (59.88)	<0.01
End Diastolic Volume indexed (ml/m^2^)	100.38 (33.42)	106.46 (36.54)	<0.01
End Systolic Volume (ml)	93.07 (36.63)	99.27 (41.52)	0.01
End Systolic Volume indexed (ml/m^2^)	53.74 (22.87)	57.43 (26.20)	0.02

**Table 3:** Bland Altman calculated levels of bias between 3D and 2D/CMRI measures, with 95% upper and lower limits of agreement

Measure	Bias	Upper limit of agreement	Lower limit of agreement
Fractional Area Change (%)	0.47	6.55	-5.62
Tricuspid Annular Plane Systolic Excursion (cm)	0.04	0.26	-0.18
RV ejection fraction (%)	0.052	7.16	-7.06
End Diastolic Volume (ml)	6.2	24.66	-12.26
End Diastolic Volume indexed (ml/m^2^)	3.68	15.44	-8.07
End Systolic Volume (ml)	10.27	38.88	-18.35
End Systolic Volume indexed (ml/m^2^)	6.08	23.5	-11.34

## ABS012 First-phase ejection fraction as a marker for timely intervention in pressure-overload rat model

Shukun He^1^, Jing Wang^1^, Haotian Gu^2^

^1^Huazhong University of Science and Technology, Wuhan, China, ^2^King’s College London, UK

*Echo Research & Practice 2026*, **13(Suppl 1):**ABS012

### Background

First-phase ejection fraction (EF1), a novel yet simple measure of early systolic function, has previously been shown to be a strong predictor of adverse outcomes in patients with aortic stenosis and is closely associated with myocardial fibrosis in pressure-overload heart failure rat models. However, it remains unclear whether early intervention—such as aortic valve replacement—can reverse early systolic dysfunction identified by EF1.

### Purpose

To evaluate, in a randomized controlled trial using an aortic banding (AB) rat model, whether EF1-guided early intervention (aortic debanding) can reverse cardiac remodeling and improve early systolic function.

### Methods

Abdominal AB was performed in 36 rats to induce pressure-overload, mimicking aortic stenosis. An additional 12 rats underwent a sham procedure and served as controls. The AB rats were randomly assigned to either an EF1-guided or EF-guided intervention group. Echocardiography was performed weekly to monitor EF1 and EF changes. In the EF1 group, debanding was triggered when EF1 showed a significant reduction compared to sham rats; in the EF group, debanding occurred when EF declined significantly. All animals were followed weekly with echocardiography after debanding and were then sacrificed for final analysis (Fig. 1).

### Results

EF1 was significantly reduced in AB rats by week 3 compared to sham controls (30 ± 1.7% vs. 34 ± 1.3%, *P*<0.01), while EF showed a significant reduction at week 6 (59 ± 2.1% vs. 62 ± 1.0%, *P*<0.01). In the EF1- guided early debanding group, both EF and EF1 improved significantly six weeks after intervention. In contrast, in the EF-guided late debanding group, neither EF nor EF1 significantly improved post-intervention (Fig. 2).

### Conclusion

EF1-guided early intervention effectively improved systolic function in a randomized controlled animal study, supporting its potential as a practical tool for timely therapeutic decision-making.

**Fig. 1(abstract ABS012):**
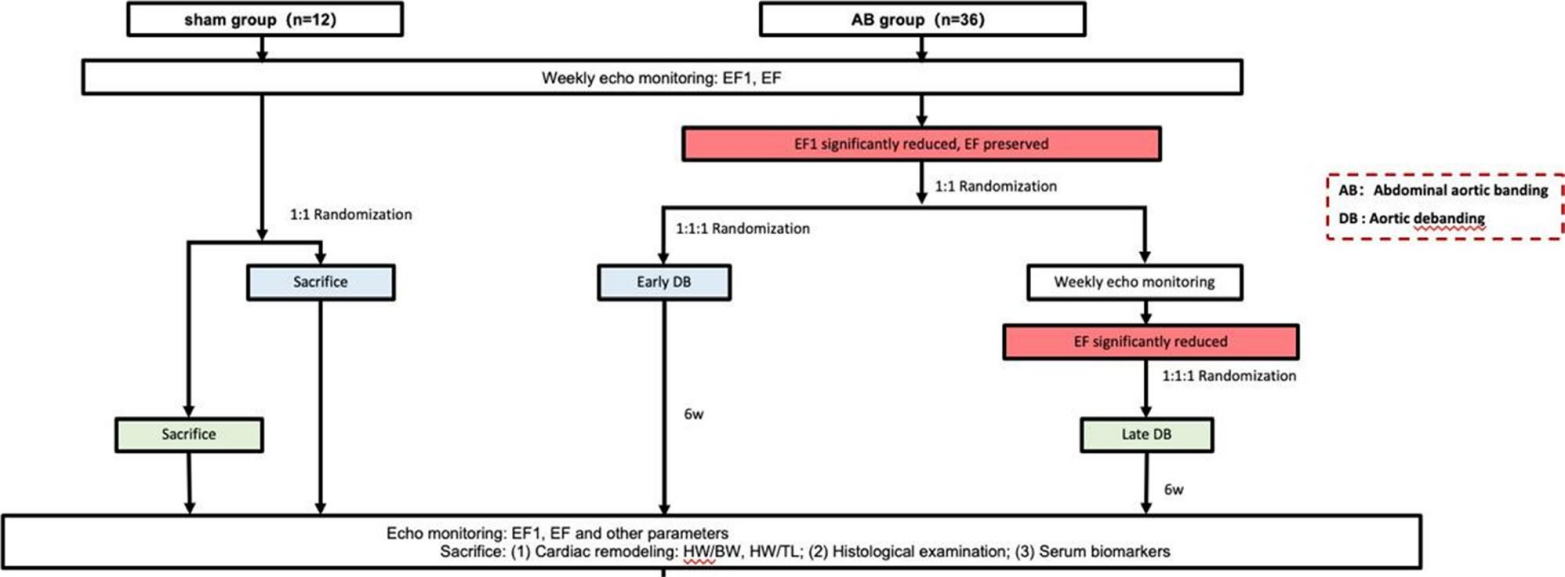
Experimental design

**Fig. 2(abstract ABS012):**
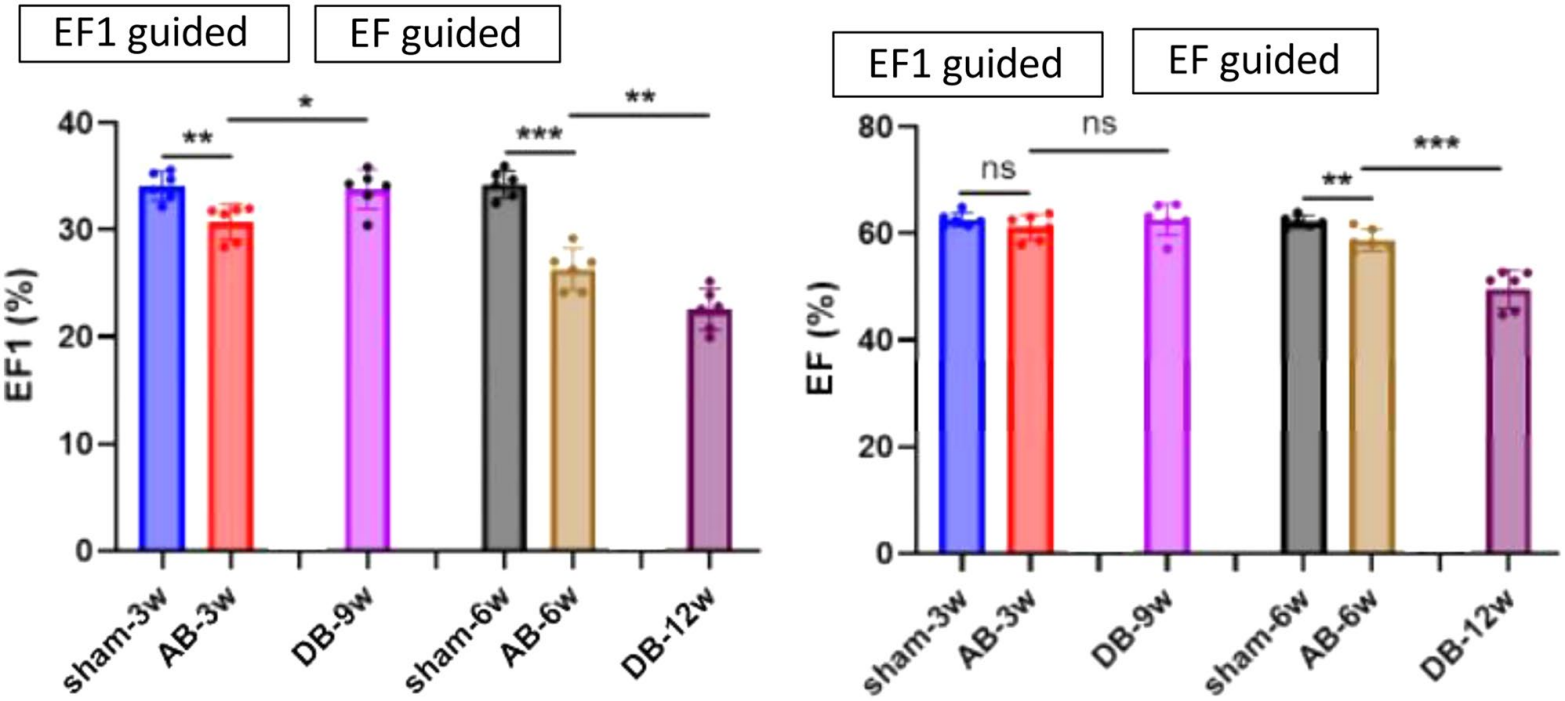
Progression of EF1 and EF in the EF1-guided and EF-guided groups

## ABS013 Calibrated integrated backscatter is associated with increased left ventricular concentricity and left atrial stiffness in resistance trained individuals using Anabolic-Androgenic Steroids

Florence Place^1^, Chloe Hamilton^1^, Harry Carpenter^1^, Luca J. Howard^1^, Barbara N. Morrison^2^, Neil Chester^1^, Robert Cooper^1^, Ben N. Stansfield^3,5^, Keith P. George^1^, Peter Angell^4^, David Oxborough^1^

^1^Research Institute for Sport and Exercise Sciences, Liverpool John Moores University, UK, ^2^School of Human Kinetics, Trinity Western University, Langley, British Columbia, Canada, ^3^Department of Pharmacology and Toxicology, College of Pharmacy, University of Arizona, USA, ^4^) School of Health and Sports Sciences, Liverpool Hope University, UK, ^5^Department of Molecular Medicine, Center for Inflammation Science and Systems Medicine, UF Scripps Institute for Biomedical Innovation and Technology, Jupiter, USA

*Echo Research & Practice 2026*, **13(Suppl 1):**ABS013

### Background

Anabolic-Androgenic Steroids (AAS) are used by resistance trained athletes. Calibrated integrated backscatter (ciB) is used to measure ultrasonic reflectivity of the myocardium and is a marker for tissue characterisation, related to fibrosis, myocyte disarray and hypertrophy, which have been documented in AAS users. This study aimed to assess resistance athletes to establish differences in ciB in users (CU) and non-users (NU) of AAS and determine any relationship to left ventricular and left atrial (LA) structure and function.

### Methods

Male (n=120) and female (n=21) resistance athletes (age 30±7 years); (CU n=95, NU n=46) were recruited. ciB was measured from a parasternal long axis orientation from the antero-septum, posterior wall and pericardium at end-diastole and calculated as the difference between pericardial intensity and the average of the posterior and anteroseptal walls. In addition, LA stiffness index (E/E’/LA reservoir strain), left atrioventricular coupling index (LA end-diastolic volume/left ventricular end-diastolic volume) and left ventricular concentricity (LV mass/LVEDV^0.667^) were calculated. Between group differences were analysed using an independent t-test or Mann-Whitney U test. Associations between ciB and structural and functional parameters were assessed using Pearsons correlation or Spearman’s rank correlation depending on distribution of data.

### Results

ciB was significantly higher in CU (-19.54 ± 4.63) than NU (-20.88 ± 3.94, *P*=.047). A significant positive correlation was observed between ciB and concentricity (r(138)=.276, *p*<.001), LV mass index (r_s_(139)=.318, *p*<.001), E/E’ (r(138)=0.169, *p*=0.043), LA conduit strain (r(135)=-0.174, *p*=0.043), LA stiffness index (r_s_(134)=0.246, *p*=0.004) and left atrioventricular coupling index (r(134)=0.241, *p*=0.005). ciB was not correlated with any parameters of left ventricular systolic function.

### Conclusions

Resistance trained athletes using AAS have a small but significantly higher ciB than non-users, potentially suggesting differences in myocardial tissue characteristics within this population. This was significantly associated with left ventricular concentricity and LA function, which may indicate a role of ciB in identifying early cardiac risk in this population.

**Fig. 1(abstract ABS013):**
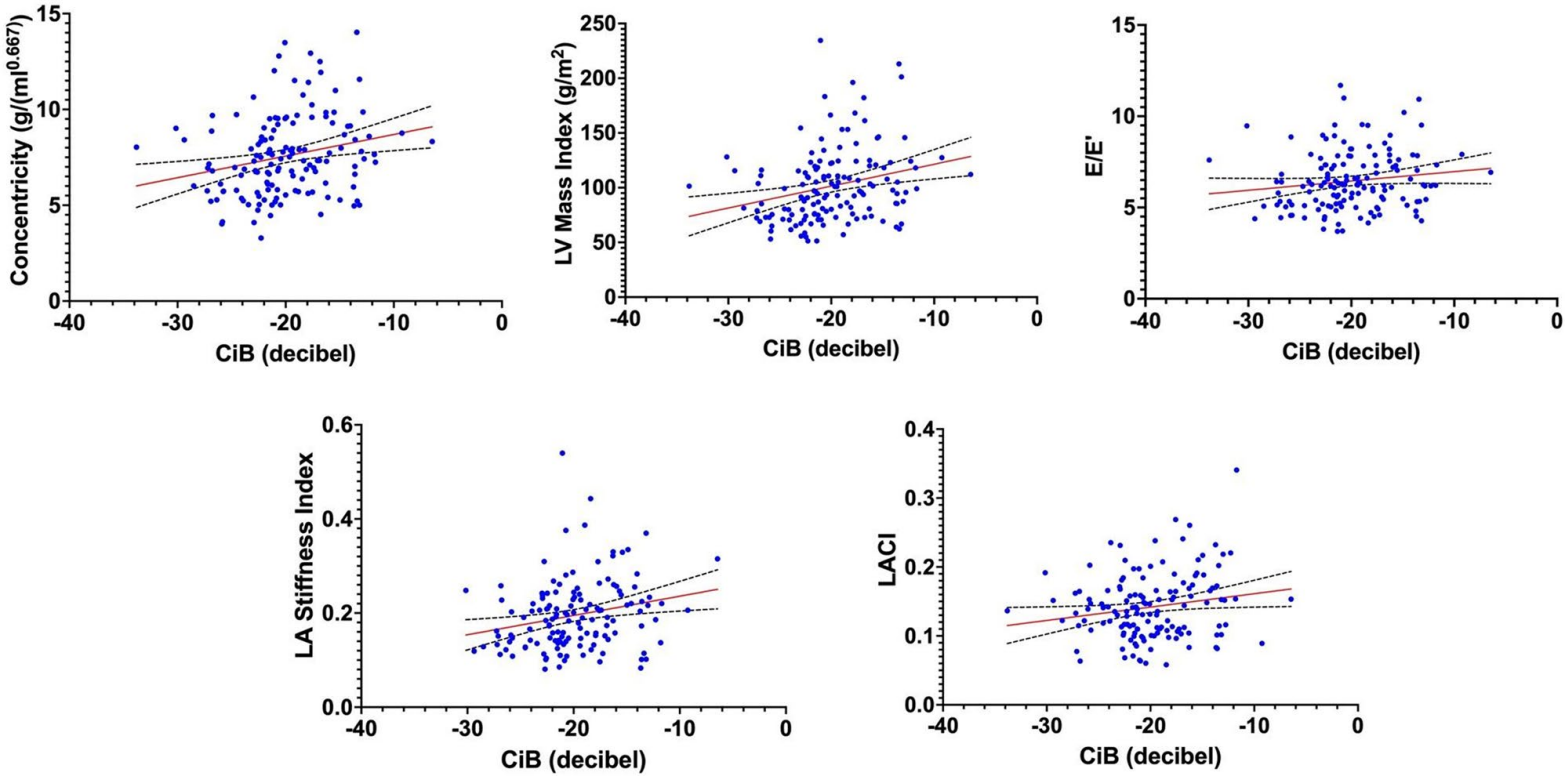
The relationship between calibrated integrated backscatter and cardiac parameters (correlation ± standard deviation). CiB = calibrated integrated backscatter, LV = left ventricular, LA = left atrial, LACI = left atrioventricular coupling index

**Fig. 2(abstract ABS013):**
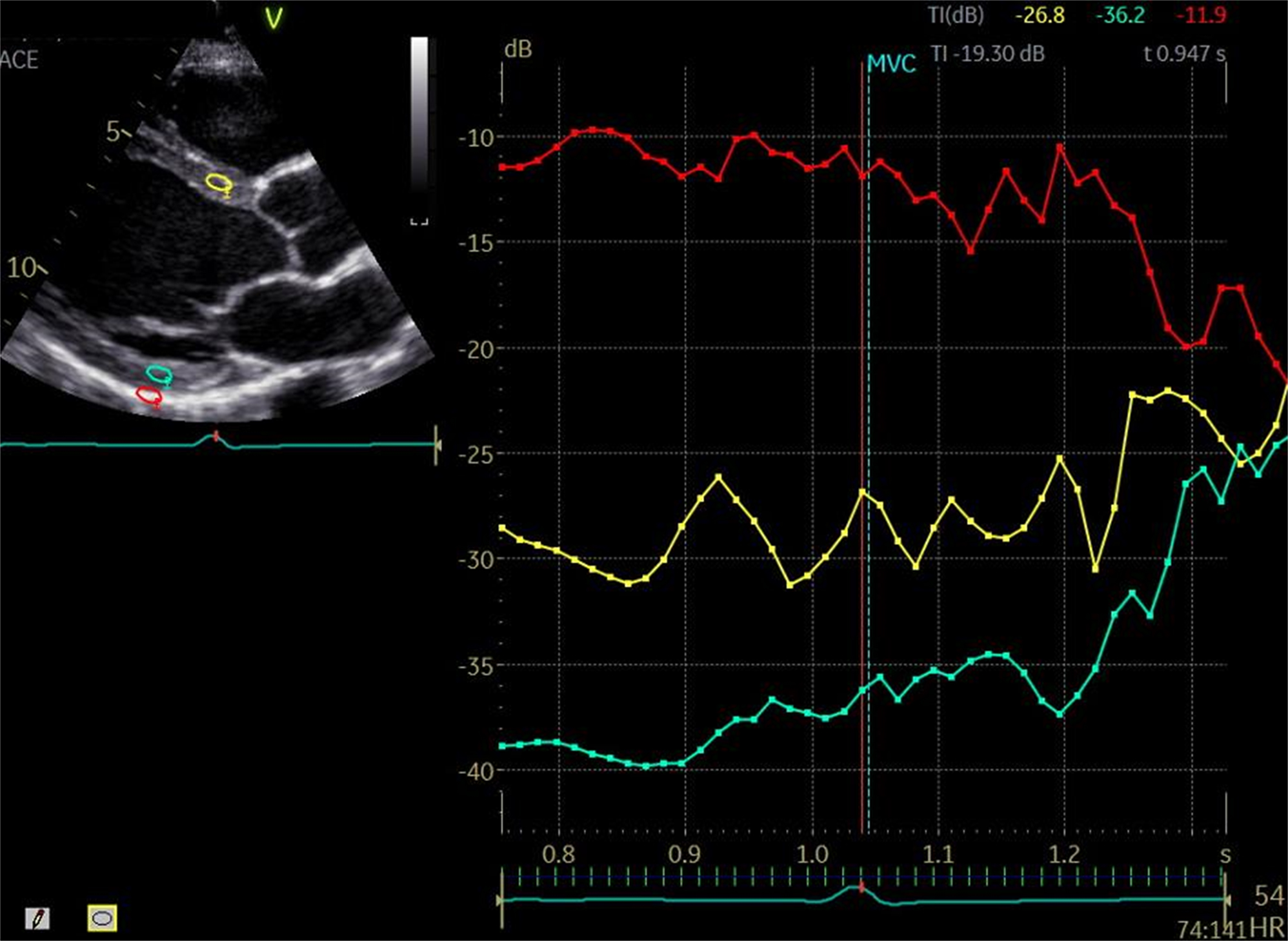
Integrated backscatter assessment of anteroseptal wall (yellow), posterior wall (blue) and pericardium (red). Used to calculate calibrated integrated backscatter (difference between the average of the posterior and anteroseptal walls and pericardium; e.g., (-26.8-36.2/2)–11.9)

**Fig. 3(abstract ABS013):**
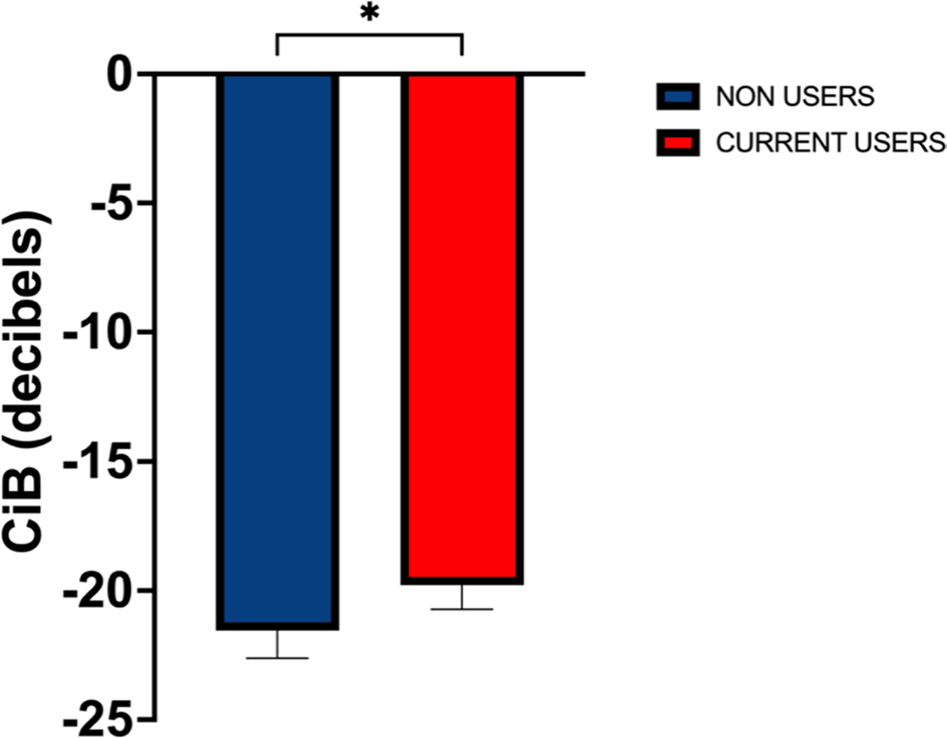
Calibrated integrated backscatter (CiB). (* Denotes significance P < 0.05)

**ABS014 Bright echogenic foci in the mitral valve in children with homozygous familial hypercholesterolaemia (HoFH): A novel finding changes echocardiographic scanning protocol for children with HoFH**

Alexandra Savis^1^, Grace Eyles^1^, Hannah Bellsham-Revell ^1^Michael Champion ^1^

^1^Evelina London Children’s Hospital, Guy’s and St Thomas’ NHS Foundation Trust, UK

*Echo Research & Practice 2026*, **13(Suppl 1):**ABS014

### Background and introduction

Homozygous familial hypercholesterolaemia (HoFH) is a rare inherited disorder of lipid metabolism that increases the risk of atherosclerotic disease in children from as early as the first decade of life. Echocardiographic assessment of HoFH has focussed primarily on cardiac function and imaging the proximal coronary arteries.

### Purpose

Mitral valve (MV) thickening and regurgitation is a very unusual finding in children with an otherwise structurally normal valve. An incidental finding of prominent focal bright areas in the MV leaflets and chordal apparatus (Figs. 1 and 2) in one patient led to a review of this cohort’s echocardiograms.

### Methods

An observational retrospective study of 11 HoFH patients (3.5-16 years) seen in a co-located paediatric cardiac and metabolic tertiary service. A review of the most recent echocardiogram was conducted to reassess the MV. All scans were performed on a Philips EpiQ 7c ultrasound system.

### Results

Of the 11 patients, 8 patients (7 to 16 years) showed evidence of small, bright echogenic foci seen in the MV leaflets and chordal apparatus.

### Conclusion

To our knowledge, this is the first reported echocardiographic finding of MV involvement in children with HoFH. Case studies describe extensive lipid deposits in the leaflets of the MV at cardiac surgery. We believe we are describing the early stages of this process. This result has led to a revision of current scanning guidelines to include qualitative assessment of the MV leaflets and apparatus in this cohort. Further longitudinal follow-up is required to see if this effect worsens or is reversible with early and targeted medical therapy.

**Fig. 1(abstract ABS014):**
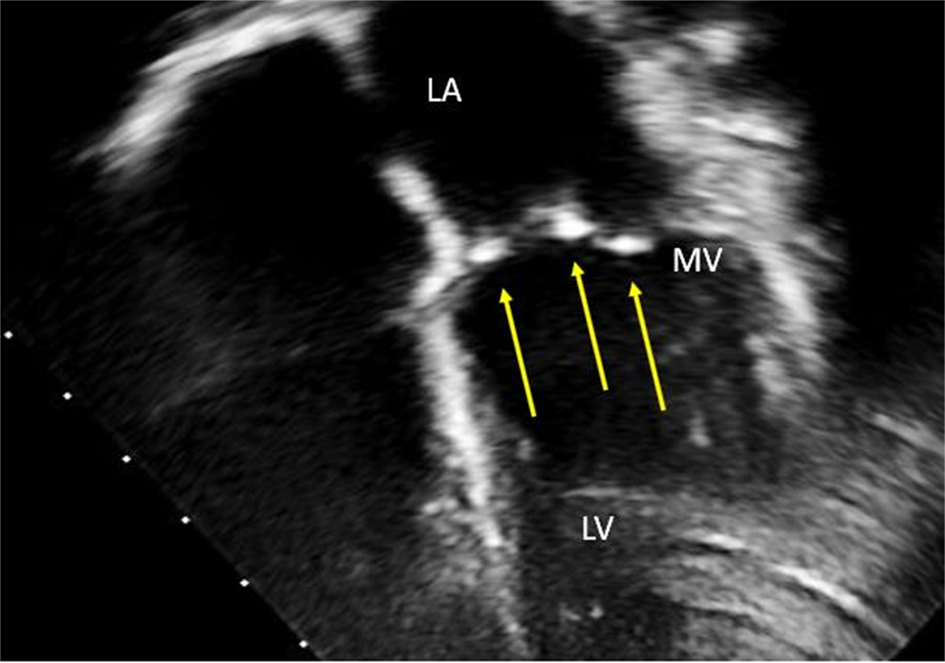
Bright echogenic foci seen in the mitral valve leaflets at the yellow arrows (MV, mitral valve: LV, left ventricle, LA, left atrium)

**Fig. 2(abstract ABS014):**
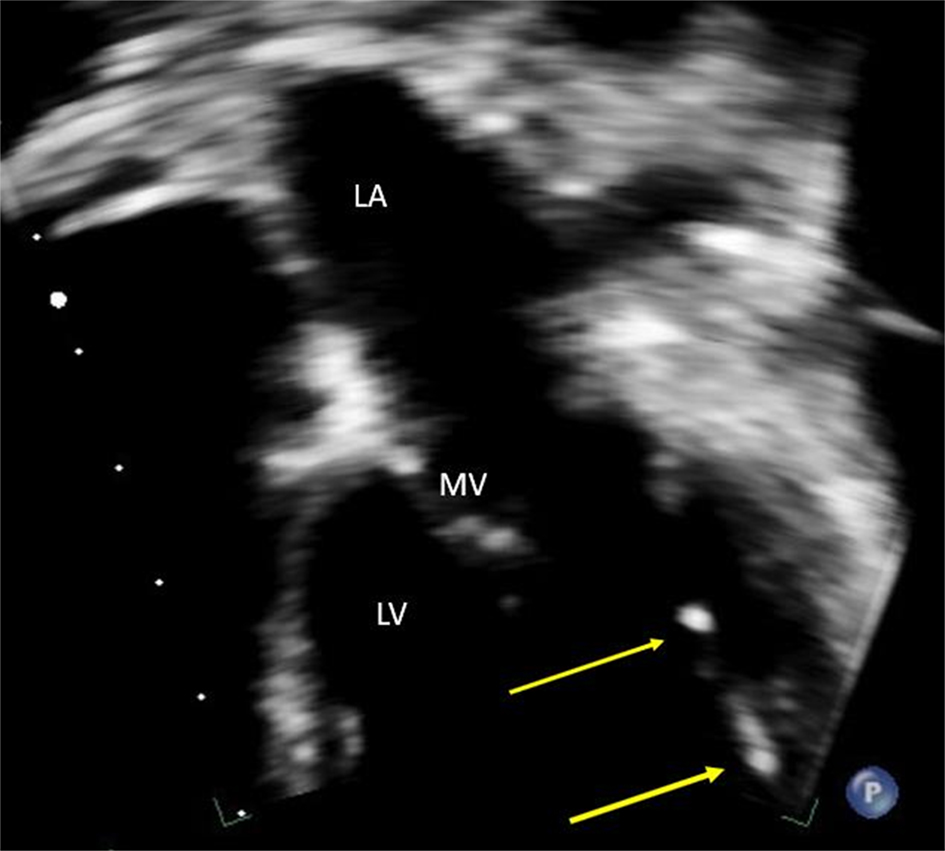
Bright echogenic foci seen in the mitral valve apparatus at the yellow arrows (MV, mitral valve: LV, left ventricle, LA, left atrium)

## ABS015 Infective endocarditis: A retrospective study investigating the relationship between disease cause and patient outcomes

Lauren Grinstead,^1^Rosanna Edwards^1^, Liam Ridge^2^

^1^Wrexham Maelor Hospital, Betsi Cadwaladr University Health Board, UK, ^2^Department of Life Sciences, Manchester Metropolitan University, UK

*Echo Research & Practice 2026*, **13(Suppl 1):**ABS015

**Background** Infective endocarditis (IE) is a rare disease associated with high mortality rates. Fewer cases of rheumatic heart disease and increasing numbers of healthcare-associated infections in developed countries has led to a shift in the epidemiological profile of the disease. An understanding of the disease profile in the local population will support clinicians in optimising patient management through the recognition of early indicators of disease progression.

**Aim** To explore the relationship between disease cause and patient outcomes in Betsi Cadwaladr Health Board patients admitted with a diagnosis of IE.

**Methods** Data from all Betsi Cadwaladr University Health Board patients admitted with a primary diagnosis of ‘Acute and sub-acute infective endocarditis’ between April 2010 and July 2023 were analysed retrospectively. To determine statistical significance between age, gender, causative pathogen, risk factors, comorbidities, and in-hospital mortality, a two-tailed Chi-square test or Fisher’s exact test was performed for each analysis. The strength of each relationship was quantified using an odds ratio. Ethical approval was granted by the Health Research Authority, Health Care Research Wales, and Manchester Metropolitan University.

**Results** Of the 248 cases included in this study, there were 28 recordings of in-hospital mortality (11%). In-hospital mortality was statistically significant in patients aged ≥60 years (*p*=.028), in those who had a previous myocardial infarction (*p*=.004), and in those with polymicrobial IE (*p*=.020). No significant gender or risk factor bias for in-hospital mortality was seen. *Staphylococcus aureus* was the predominant pathogen (30%) and was causative in 40% of patients with hepatitis C (*p*=.022). Other strep was responsible for 29% of all cases. The most prevalent comorbidities were kidney disease (23%) and essential hypertension (21%).

**Conclusion** This pilot study suggests in-hospital mortality of patients with IE is significantly affected by disease cause and patient profile. Patients ≥60 years of age, those with a history of previous MI, and those with polymicrobial IE have an increased risk of in-hospital mortality. A comprehensive study with full access to patient records would allow for analysis of patient management with respect to patient mortality. This has the potential to highlight ways of optimising the management of these high-risk patients for improved outcomes.

## ABS016 Predictors of outcomes in atrial functional mitral and tricuspid regurgitation

Jawza Aldakhil^1,2^, Kush Patel^2^, Sveeta Badiani^2^, Steffen E. Petersen^1,2^, Guy Lloyd^1,2^, Sanjeev Bhattacharyya^1,2^

^1^William Harvey Research Institute, Queen Mary University London, UK, ^2^Barts Health NHS Trust, London, UK

*Echo Research & Practice 2026*, **13(Suppl 1):**ABS016

**Background** Atrial functional mitral and tricuspid regurgitation (AFMR/AFTR) arise from longstanding atrial fibrillation (AF). Survival predictors in this population remain poorly defined.

**Objectives** To identify clinical and echocardiographic predictors of all-cause mortality in patients with AFMR and/or AFTR.

**Methods** Adults with AF and ≥ moderate MR and/or TR who underwent transthoracic echocardiography between 2017 and 2021 were retrospectively reviewed. Patients with structural heart disease, left-ventricular ejection fraction <50%, or sub-optimal image quality were excluded.

**Results** A total of 151 patients were included. 54 with isolated AFTR, 25 with isolated AFMR, 37 with combined disease, and 35 AF controls with mild or no regurgitation. Mean follow-up was 4±2.6 years, during which 71 patients (47%) died. LA reservoir strain (LASr) progressively worsened from controls (11.3±5.3%) to AFTR (9.0±3.9%), combined disease (8.9 ± 3.4%) and AFMR (7.2 ± 3.6%); p=0.004) (Table 4). ROC analysis identified LASr ≤ 6.5% as the optimal cut-off for predicting mortality. (AUC 0.620, sensitivity 48%, specificity 81%.) Kaplan-Meier analysis showed significantly lower survival in patients with LASr ≤6.5% (Fig. 1). In Cox regression univariable analysis, age, LASr ≤6.5 %, hypertension, pulmonary artery systolic pressure, and right-atrial strain ≤11.5 % predicted mortality. In the multivariable model, age (HR 1.088 per year, 95% CI: 1.041–1.137; p<0.001) and LASr ≤6.5 % (HR 2.32, 95% CI: 1.17–4.59; p=0.016) remained strong independent predictors (Table 5).

**Conclusion** Older age and reduced LASr (≤6.5%) are powerful, independent predictors of all-cause mortality in patients with AFMR and/or AFTR.

**Table 4:** Demographic and echocardiographic characteristics

Variable	Control (n=35)	AFMR (n=25)	AFTR (n=54)	Combined (n=37)	p-value
Age (years)	71.60±10.11	81.24±7.77	78.81±11.14	79.97±9.09	**<0.01**
Gender (Female)	12 (34%)	15 (60%)	35 (65%)	24 (65%)	**0.02**
Diabetes	1 (8%)	8 (32%)	14 (27%)	7 (19%)	0.13
Hypertension	5 (39%)	17 (68%)	29 (55%)	32 (89%)	**<0.01**
Hypercholesterolaemia	1 (8%)	4 (16%)	10 (19%)	11 (31%)	0.23
Smoking	3 (23%)	7 (29%)	10 (20%)	4 (11%)	0.32
Myocardial Infarction	1 (8%)	3 (12%)	8 (15%)	9 (25%)	0.26
LASr (%)	11.30±5.26	7.22±3.64	9.00±3.90	8.88±3.41	**0.004**
LAV (ml)	85.31±24.19	127.08±70.20	99.63±31.59	128.08±87.11	**0.003**
LV EDV indexed (ml/m^2^)	46.39±8.54	55.30±14.53	47.12±18.73	54.53±12.73	0.072
LVEF (%)	59.00±7.90	66.22±11.95	60.96±9.06	61.47±9.64	**0.044**
GLS (%)	-15.05±7.24	-14.20±3.83	-15.59±3.38	-15.04±3.87	0.800
TAPSE (cm)	1.95±0.50	1.62±0.35	1.75±0.42	1.69±0.46	**0.027**
PASP (mmHg)	31.80±8.51	41.05±15.88	40.28±13.34	42.83±18.28	**0.044**
MR EROA (cm^2^)	-	0.239±0.153	-	0.215±0.115	0.528
TR EROA (cm^2^)	-	-	0.322±0.149	0.314±0.285	0.865

LASr, Left Atrial Reservoir Strain; LAV, Left Atrial Volume; LV EDV, Left Ventricle End Diastolic Volume; LVEF, Left Ventricle Ejection Fraction; GLS; Global Longitudinal Strain; TAPSE, Tricuspid Annular Plane Systolic Excursion; PASP, Pulmonary Artery Systolic Pressure; MR EROA, Mitral Regurgitation Effective Regurgitant Orifice Area; TR EROA, Tricuspid Regurgitation Effective Regurgitant Orifice Area

**Table 5:** Univariable and multivariable cox regression analysis

Variable	HR (95% CI)	P value	HR (95% CI)	P value
**Univariate**			**Multivariate**	
Age	1.075 (1.044–1.107)	<0.001	1.088 (1.041–1.137)	<0.001
Hypertension	1.952 (1.087 – 3.506)	0.025	1.113 (0.530–2.336)	0.778
PASP (mmHg)	1.013 (1.001–1.025)	0.029	1.007 (0.990–1.024)	0.427
LASr (≤6.5%)	2.649 (1.599–4.388)	<0.001	2.319 (1.171–4.594)	0.016
RASr (≤11.5%)	2.822 (1.569–5.077)	<0.001	2.143 (1.024–4.487)	0.043

PASP, Pulmonary Artery Systolic Pressure; LASr, Left Atrial Reservoir Strain; RASr, Right Atrial Reservoir Strain

**Fig. 1(abstract ABS016):**
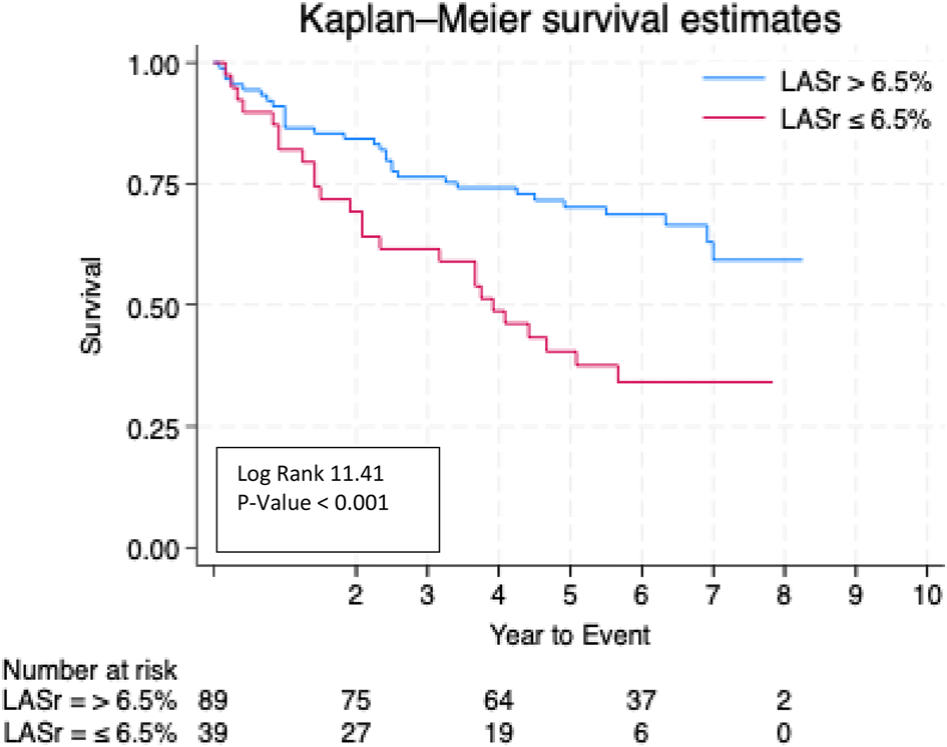
Kaplan Meier survival for all-cause mortality by LASr cut-off (≤ 6.5 % vs > 6.5 %)

## ABS017 Children of UPBEAT: Child heart structure and function 10 years after the UPBEAT lifestyle intervention in obese pregnancy

Samuel J. Burden^1^, Simrat Kaur^1^, Rahaf Alshehri^2^, Ernestene Vasa^2^, Carolyn Gill^1^, Leyre Villar^1,3^, Scott Nelson^4^, Naveed Sattar^5^, Mark Anderson^6^, Reena Perchard^7^, Federico Fiori^8,9^, Paramala Santosh^8,9^, Manish Sinha^10,11^, Lucilla Poston^1^, Paul D. Taylor^1^, Haotian Gu^2^, on behalf of the UPBEAT consortium

^1^Department of Women and Children’s Health, School of Life Course and Population Sciences, King’s College London, UK, ^2^Cardiovascular Medicine and Science Research, School of Cardiovascular and Metabolic Medicine & Sciences, King’s College London, UK, ^3^University Hospital Carl Gustav Carus, Dresden, Germany, ^4^School of Medicine, Dentistry & Nursing, Reproductive & Maternal Medicine, University of Glasgow, UK, ^5^School of Cardiovascular and Metabolic Health, BHF Glasgow Cardiovascular Research Centre, University of Glasgow, UK, ^6^Department of Paediatric Medicine, The Newcastle upon Tyne Hospitals NHS Foundation Trust, UK, ^7^Division of Developmental Biology and Medicine, Manchester Academic Health Science Centre, University of Manchester, UK, ^8^Institute of Psychiatry, Psychology and Neuroscience, King’s College London, UK, ^9^South London and Maudsley NHS Foundation Trust, UK, ^10^Department of Paediatric Nephrology, Evelina London Children’s Hospital, UK, ^11^King’s College London British Heart Foundation Centre, UK

*Echo Research & Practice 2026*, **13(Suppl 1):**ABS017

**Background** Children exposed to maternal obesity in pregnancy are more likely to have altered heart structure and function and develop adult cardiovascular disease. There is evidence that diet and lifestyle interventions during obese pregnancy can limit the degree of cardiac remodelling, but existing studies are limited to small cohorts of younger children (<7-years-old). Here, we present an interim analysis of children of UPBEAT – a 10-year cardiovascular follow-up of children from the UK Pregnancies Better Eating and Activity Trial (UPBEAT).

**Methods** A cross-sectional, longitudinal follow-up study of 9–14-year-old children from UPBEAT was conducted in four centres across the UK (London, Glasgow, Manchester, Newcastle; Ethics Committee #23/LO/0410). Children had cardiac structure and function assessed by echocardiography, vascular stiffness by B-mode ultrasound and applanation tonometry, and blood pressure and heart rate variability by 36-hour ambulatory wearable devices.

**Results** An interim analysis of n=100 children found that the UPBEAT intervention reduced interventricular septal wall thickness (IVS: -0.06cm, p=0.002), IVS z-score (-0.39, p=0.003), relative wall thickness (-0.02, p=0.054), and increased peak septal wall early diastolic myocardial velocity (e’: +0.8cm/s, p=0.023). There were differences in asleep, log adjusted ambulatory HRV (low frequency power, p=0.049; high frequency power, p=0.029; LF/HF ratio [indicative of reduced sympathovagal balance], p=0.038). There were no apparent differences in ambulatory blood pressure recordings or other echocardiography, vascular stiffness, or HRV measures between the UPBEAT intervention and standard care arms. As of 26^th^ June 2025, n=16 participant visits have been completed. At BSEcho 2025, we will present updated results from n>200 children.

**Conclusions** Preliminary findings indicate that the UPBEAT lifestyle intervention in obese pregnancy reduces the degree of cardiovascular remodelling and autonomic dysfunction in 9–14-year-old children, which if sustained until adulthood, could provide primordial prevention against adult cardiovascular disease.

## ABS018 First-phase ejection fraction: A secondary analysis in the SABRE study

Rahaf M. Alshehri^1^, Nish Chaturvedi^2^, Charlotte Manisty^2^, Chloe Park^2^, Andrew Wong^2^, Alun Hughes^2^, Phil Chowienczyk^1^, Haotian Gu^1^

^1^King’s College London, UK, ^2^University College London, UK

*Echo Research & Practice 2026*, **13(Suppl 1):**ABS018

**Background** First-phase ejection fraction (EF1), a novel and simple, yet robust measure of early left ventricular systolic function, has emerged as a promising tool in assessing early systolic dysfunction. This study aimed to evaluate the prognostic value of EF1 in the SABRE study – a large, tri-ethnic, community-based cohort.

**Methods** EF1 was retrospectively analysed from echocardiography in 1,127 participants (mean age 69.6 ±6.1 years) and defined as the percentage change in left ventricular volume from end-diastole to the time of peak aortic flow velocity, using Simpson’s biplane method. The primary outcome was all-cause mortality.

**Results** Over a median follow-up of 8 years, 159 deaths were recorded. Left ventricular ejection fraction (LVEF) and EF1 were significantly lower in non-survivors compared to survivors (LVEF: 58.7±11%vs 62±9.5%, p<0.0001; EF1: 20.1±9.3% vs 28.5±8.6%, p<0.001), whereas global longitudinal strain (GLS) did not differ (Table 6). In univariable Cox-regression analysis, EF1 was a strong predictor of all-cause mortality, and remained the strongest predictor of events after adjustments for confounders (HR: 0.89, 95% CI: 0.87-0.92, p<0.001), outperforming LVEF and other echo measures (Table 7). ROC analysis identified an optimal cut-off value of 22% for EF1. Kaplan-Meier analysis confirmed that EF1<22% was strongly associated with all-cause mortality (Fig. 1).

**Conclusion** EF1 is an independent and powerful predictor of all-cause mortality in a large multi-ethnic, community-based population. It may be useful for identifying early systolic function in the general population.

**Table 6:** Baseline characteristics and echocardiographic measurements between survivors and non-survivors. n= 1127

Variables	Survivors n=968	Non-survivors n=159	p-value
Age, (years)	68.9±5.8	74± 6.2	<0.001
Sex: Male Female	729 245	129 30	0.086
BMI, (kg/m2)	27.1±4.1	26.4±4.3	0.045
HR, (bpm)	64±13.5	65.3±15	0.271
SBP (mmHg)	138.9±17.7	143.6±21	0.002
DBP (mmHg)	76.7±9.7	74.8±10.4	0.026
NT ProBNP (pg/mL)	163.6±11.1	547±85.1	<0.001
LV EDV (ml)	92.6±22.4	95.7±30.3	0.081
LVMi (g/m^2^)	93.6±22.2	101.2±25.4	0.001
LAVi (ml/m^2^)	23.4±8.8	25.9±10.9	0.001
E/e’	9.3±3.06	10.8±4.7	<0.001
LVEF, %	62±9.5	58.7±11	0.0001
GLS, %	-19.07±2.8	-18.6±3.9	0.284
EF1, %	28.5±7.7	20.1±9.3	<0.001
TPAVF	141.2±22.6	139±24.3	0.254

BMI: body mass index, HR: heart rate. SBP: systolic blood pressures, DBP: diastolic blood pressures, NT BNP: N-terminal pro B-type natriuretic peptide, LV EDV: left ventricular end-diastolic volume, LVMi: Left ventricular mass index, LAVi: left atrial volume index, LVEF: left ventricular ejection fraction, GLS: global longitudinal strain, TPAVF: time to peak aortic valve flow

**Table 7:** Cox regression analysis

Univariate					Multivariate			
Variables	HR	95% CI		P value	HR	95% CI		P value
Age	1.13	1.1	1.15	<0.001	1.06	1.03	1.10	<0.001
Sex	0.71	0.47	1.06	0.111				
BMI	0.96	0.92	0.99	0.045	0.96	0.91	1.02	0.238
SBP	1.01	1,00	1,02	0.003	1.01	1.00	1.02	0.027
DBP	0.98	0.96	0.99	0.017	0.97	0.95	0.99	0.035
NT ProBNP	1.00	1.0003	1.0005	<0.001	0.99	0.99	1.00	0.797
Troponin	1.04	1.03	1.05	<0.001	1.02	1.01	1.04	0.001
Ethnicity								
South Asian African Caribbean	1.05 0.65	0.75 0.39	1.47 1.09	0.760 0.110				
LV EDV	1.00	0.99	1.01	0.127				
LVMi	1.01	1.00	1.02	0.008	0.99	0.98	1.01	0.888
E/e’	1.08	1.05	1.11	<0.001	1.00	0.95	1.05	0.965
E/A	0.37	0.17	0.79	0.011	0.5	0.19	1.28	0.153
TPAVF	0.99	0.98	1.00	0.247				
LVEF	0.97	0.95	0.98	<0.001	1.00	0.98	1.02	0.841
EF1	0.87	0.85	0.89	<0.001	0.89	0.87	0.92	<0.001

Multivariate Cox regression analysis was adjusted for significant confounders, including age, sex, BMI, systolic and diastolic blood pressure.

**HR: hazard ratio (per 1-unit change), CI: confidence interval.**

BMI: body mass index, NT Pro BNP: N-terminal pro B-type natriuretic peptide, LV EDV: left ventricular end-diastolic volume, LVMi: Left ventricular mass index, LVEF: left ventricular ejection fraction, GLS: global longitudinal strain, TPAVF: time to peak aortic valve flow

**Fig. 1(abstract ABS018):**
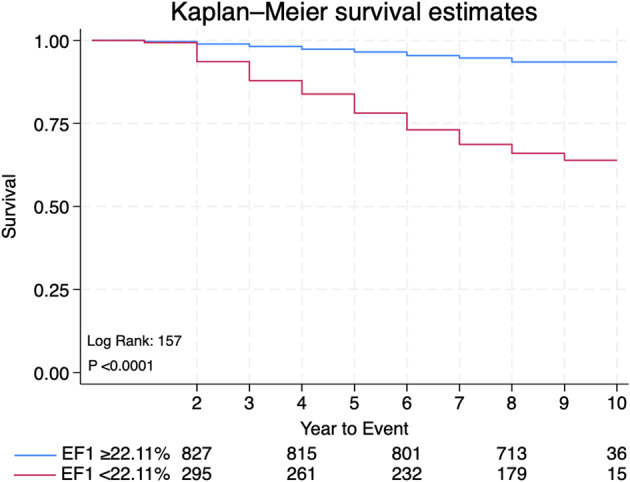
Kaplan-Meier curve of EF1 cut-off value of 22.11% in the total population

**ABS019 Echocardiography screening for pulmonary hypertension in high-risk sickle cell disease patients in a dedicated cardiology-haemoglobinopathy clinic**

Ana Ferreira^1,2^, Jose Bingcang^1,2^, Perla Eleftheriou^3^, Malcolm Walker^2^, Polyvios Demetriades^1,2^

^1^Barts Heart Centre, Barts Health NHS Trust, London, UK, ^2^Cardiology, University College London Hospital, NHS Foundation Trust, UK, ^3^Haematology, University College London Hospital, NHS Foundation Trust, UK

*Echo Research & Practice 2026*, **13(Suppl 1):**ABS019

**Background** Pulmonary hypertension (PH) is a serious and increasingly recognised complication in adults with sickle cell disease (SCD), with a reported prevalence of 6–11% and associated with significantly increased mortality. Approximately 40% of cases have pre-capillary, whilst the remaining have mixed/post-capillary PH, defining treatment strategies.

**Purpose** This project aims to evaluate echocardiographic screening in high-risk SCD patients seen in a dedicated cardiology-haemoglobinopathy clinic.

**Methods** We retrospectively collected clinical and echocardiographic data from SCD patients reviewed in the past 6 months.

**Results** We identified 75 patients (male=37%, mean age=47, HbSS=91%), 39% on exchange transfusions (RCE) and 19% on hydroxyurea (HU). Fourteen patients (19%) had high probability (HP) of PH as per BSE criteria (mean TR VMax 3.5 m/s, mean PASP 59mmHg). Nine (12%) of these were referred for right heart catheterisation (RHC) of which six (8%) have confirmed PH and four (5%) were commenced on pulmonary vasodilators. Remaining patients with HP-PH did not have RHC due to left heart disease or being unfit for procedure. In addition, it was noted that 29% (n=22) had impaired LV function (LVSD).

**Conclusions** We identified a high proportion of patients with HP-PH. This likely reflects selection bias in a high-risk group under specialist care, evident by the high percentage of patients on RCE/HU and with LVSD. RHC remains the gold standard in diagnosing PH, however in our practice it is reserved for those with potential to start pulmonary vasodilators (without LVSD). Increasing availability of specialist haematology care alongside early introduction of advanced treatments (RCE/HU) likely accounts for the relatively low incidence pre-capillary PH in our population. In conclusion, echocardiography remains a useful first-line screening tool for PH in SCD. In the future, we aim to investigate the correlation of pulmonary pressures with timing of transfusions.

## ABS020 Left ventricular diastolic function assessment: a quality improvement project

Rehan Akhtar^1^, Mike Rees^1^, Adele Oxborough^1^, Chris Hunt^1^, James Redfern^1^

^1^Countess of Chester Hospital, UK

*Echo Research & Practice 2026*, **13(Suppl 1):**ABS020

**Background/introduction** As treatment options for the management of heart failure with preserved ejection fraction (HFpEF) have expanded, the importance of accurate assessment of diastolic dysfunction has increased.

**Purpose** To improve the reporting of left ventricular diastolic function (LVDF) and adherence to British Society of Echocardiography (BSE) guidelines in patients undergoing transthoracic echocardiography (TTE) at a district general hospital.

**Methods** The TTE reports of 50 patients were reviewed at baseline, and one month after each intervention. Patients undergoing a BSE Level 3 TTE study were included. Focused or incomplete studies were excluded. The Plan, Do, Study, Act methodology was utilised to enact change. During the first cycle, a short lecture on diastolic physiology was delivered at the departmental meeting and data collected the following month (February 2024). Subsequently, a poster based on the unpublished BSE guidelines on LVDF (Fig. 1) was displayed in the department (April 2024). Following publication of the completed guidelines, teaching on the update was provided, in addition to roll-out of recommended reporting terms in the reporting software (October 2024).

**Results** At baseline, 38% of reports included a comment on overall LVDF. This rose to 88% following the series of interventions (Fig. 2). A substantial increase in the reporting of age-specific e’ in eligible patients (0% to 78%) was also noted.

**Conclusion** A substantial improvement in reporting of LVDF and age-specific e’ was achieved by implementing the BSE Diastolic Function Guidelines. With new echo machines, we plan to expand the use of LA strain assessment for indeterminate studies.

**Fig. 1(abstract ABS020):**
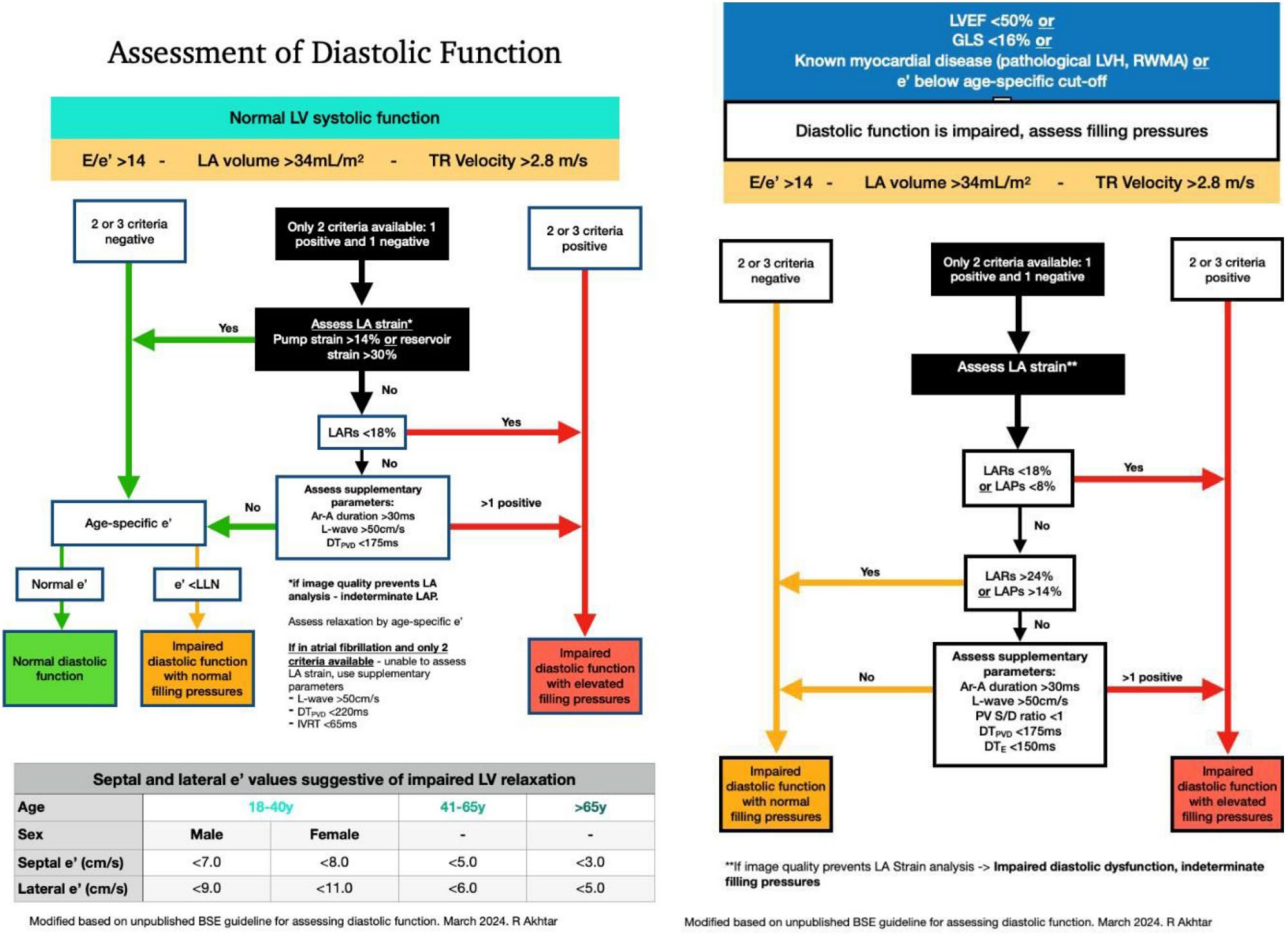
Posters developed during the second cycle based on unpublished BSE guidance

**Fig. 2(abstract ABS020):**
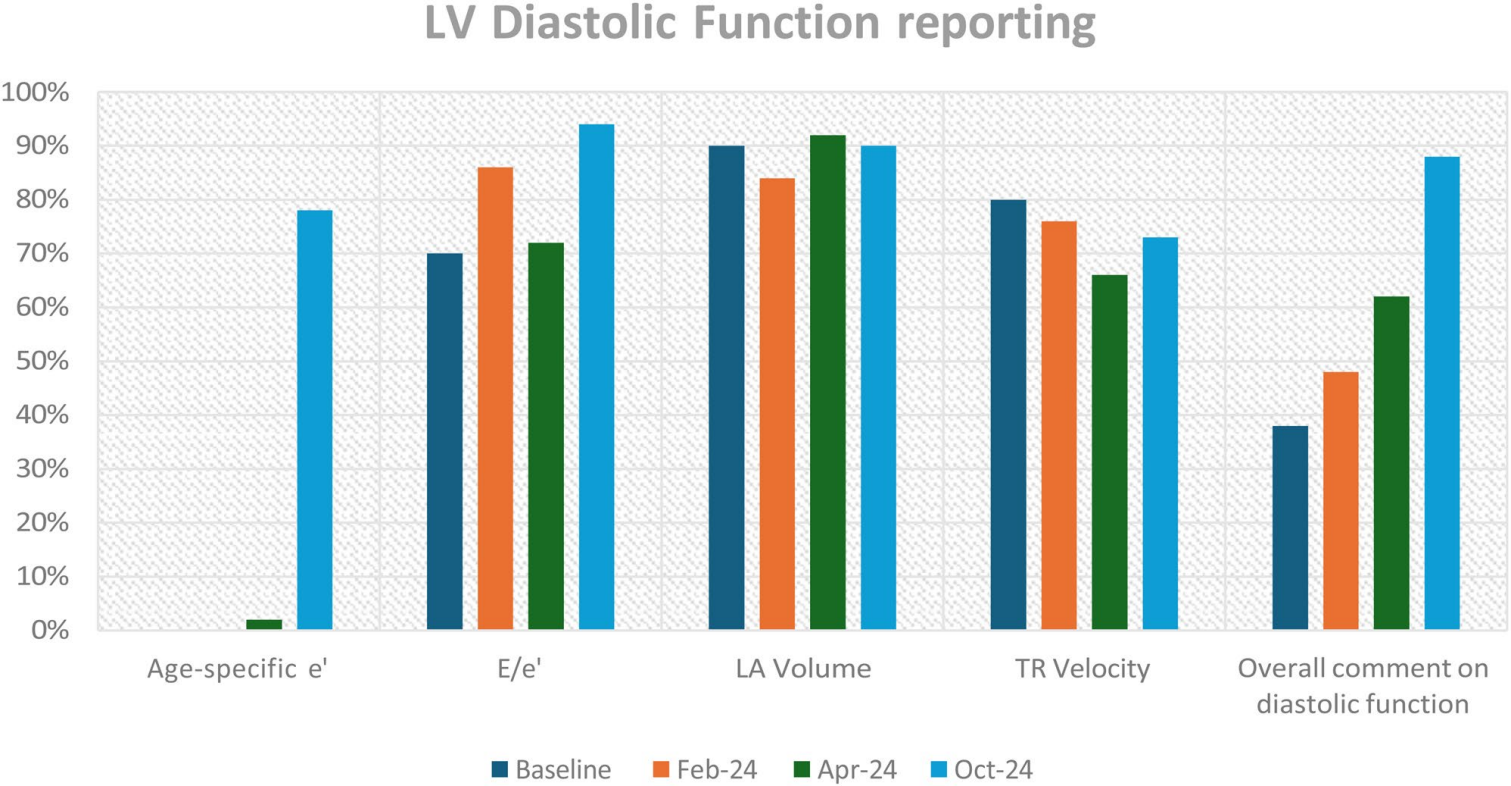
Overall results, at baseline and then following the three interventions

**ABS022 Implementing a physiologist-led echocardiography clinic for survivors of childhood cancer: A novel model for late eﬀects cardio-oncology service delivery**

Ana Ferreira^1,2^, Jose Bingcang^1,2^, Victoria Grandage ^3^, Rachel Windsor ^3^, Katrina Ingley ^3^, Alison Webb ^3^, Alison MacKlin^3^, Micaela Plucinski^3^, Sanjeev Bhattacharyya ^1,2^, Arjun K. Ghosh^1,2^, Polyvios Demetriades^1,2^

^1^Barts Heart Centre, Barts Health NHS Trust, London, UK, ^2^Cardiology Service, University College London Hospital NHS Foundation Trust, UK, ^3^Haematology/Oncology Service, University College London Hospital, NHS Foundation Trust, UK

*Echo Research & Practice 2026*, **13(Suppl 1):**ABS022

**Background** Survivors of childhood and adolescent cancer are at risk of delayed cardiotoxicity due to prior anthracycline chemotherapy and/or chest radiotherapy. International guidelines recommend routine echocardiographic surveillance to enable early cardiotoxic detection (Table 8). With increasing demand on cardio-oncology services, innovative delivery models are needed. While physiologist-led clinics are established in cardiology, this is the first known application in a late effects setting.

**Purpose** To describe the implementation and early outcomes of a physiologist-led echocardiographic surveillance clinic tailored to cancer survivors.

**Methods** The service targets individuals diagnosed before age 25, now >5 years post-treatment, with prior anthracycline and/or chest radiotherapy exposure. Medium and high-risk patients are triaged to a physiologist-led clinic for echocardiography and basic clinical review. Escalation pathways are in place to cardio-oncology or haematology where indicated (Fig. 1). The physiologist also completes documentation and patient correspondence.

**Results** Over a period of 1 year, 123 survivors have been referred (mean age 29 years; 47% female). To date, 52 patients have been reviewed. Of these, 18 (35%) required further action. Four patients with borderline left ventricle ejection fraction were referred for cardiac MRI and intensified follow-up; one was referred to a consultant-led clinic. Other indications included palpitations, prolonged QTc, and chest pain, prompting Holter, cardiac MRI, CTCA or biomarker testing. Additional referrals included Haematology late effects (n=3), GP for blood pressure management (n=4) and counselling (n=2). Patients without symptoms or concerning findings remain under routine surveillance.

**Conclusion** This is the first physiologist-led echo clinic dedicated to late effects cardio-oncology care. The model is scalable, patient-centred and guideline-compliant, supporting timely detection and escalation of cardiac issues in cancer survivors. It reduces hospital visits, optimises consultant capacity, and ensures structured surveillance. Crucially, it expands the physiologist’s role with reported gains in clinical ownership and job satisfaction.

**Table 8:**
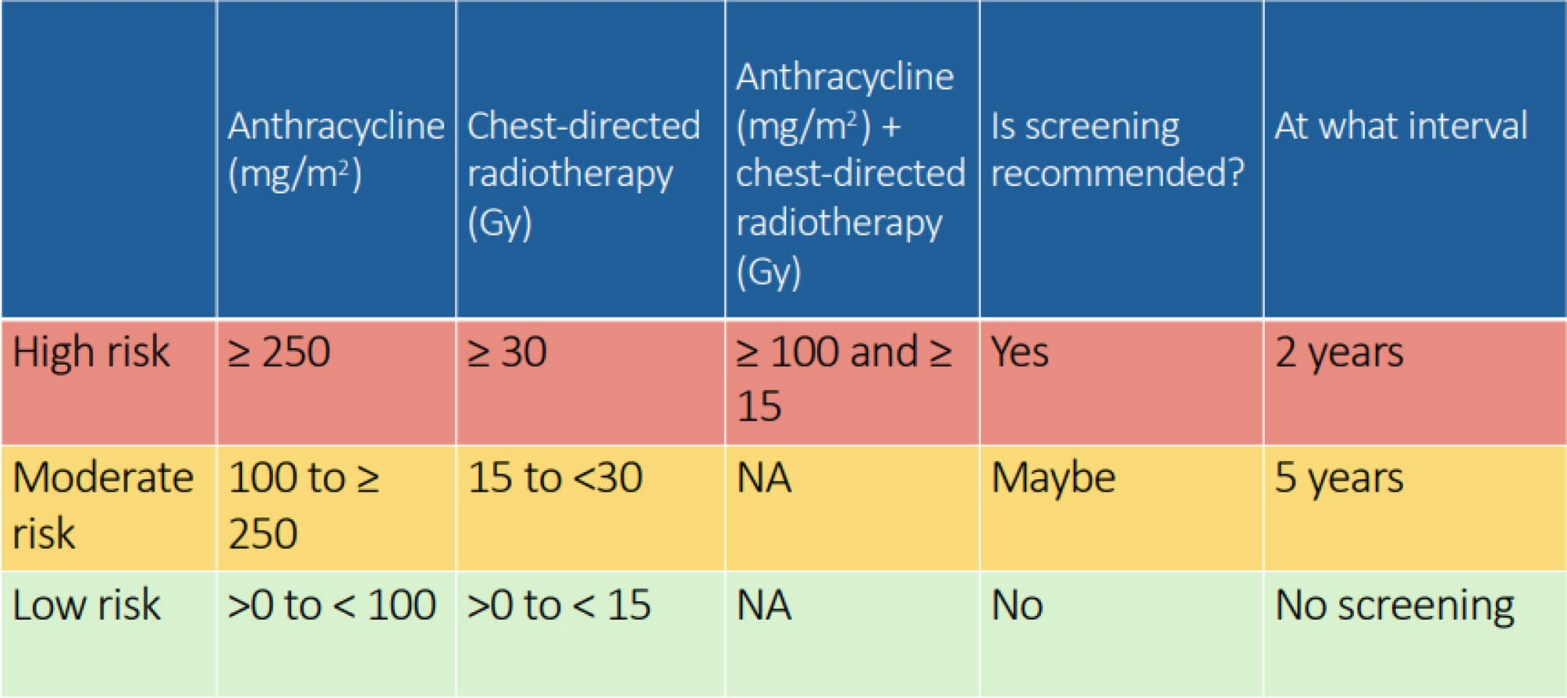
Recommended frequency of echocardiogram

**Fig. 1(abstract ABS022):**
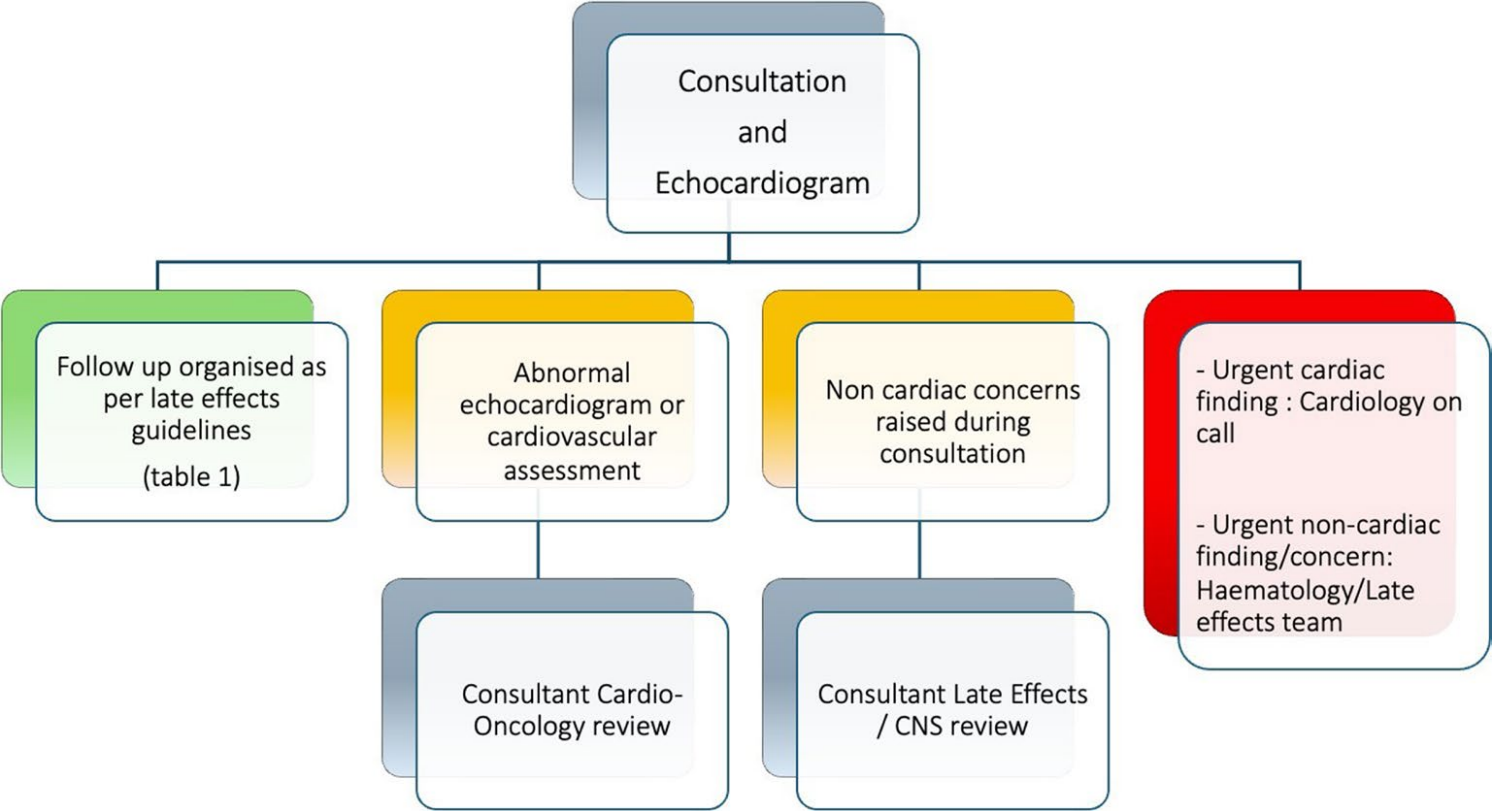
Escalation of ﬁndings from the physiologist-led late eﬀects clinic

## ABS023 Impact of British Society of Echocardiography triaging guidelines on inpatient echocardiography referrals: A 3-month retrospective audit

Aliki Tsagkridi^1,2^, Jose Bincgang^1,2^, Ana Ferreira^1,2^, Isha Mehta^1,2^, Ruth Outschoorn^1,2^, Alexander Sirker^1,2^Sanjeev Bhattacharyya^1,2^

^1^Barts Heart Centre, Barts Health NHS Trust, London, UK, ^2^Cardiology department, University College London Hospitals NHS Foundation Trust, UK

*Echo Research & Practice 2026*, **13(Suppl 1):**ABS023

**Background** Rising demand for inpatient transthoracic echocardiography requires efficient triaging to optimise resources and ensure timely care. The British Society of Echocardiography triaging guidelines provide a standardised framework to assess referral appropriateness.

**Purpose** To evaluate the impact of implementing BSE triage guidelines on inpatient echocardiography referrals, focusing on appropriateness, rejection rates, resource utilisation, and service implications.

**Methods** A retrospective audit of 487 inpatient echocardiography referrals between July and October 2024 was conducted across multiple specialties. Initial triaging was performed by cardiac physiologists using BSE criteria, with complex cases escalated to the echocardiography consultant via EPIC, the hospital’s electronic health record system. Appropriateness was retrospectively assessed by the lead author.

**Results** Of 487 referrals, 170 (35%) were deemed inappropriate. From those, 111 (23%) were formally rejected. Main reasons for rejection included inappropriate clinical indications (55%), recent normal echoes with normal biomarkers (23%), requests unrelated to current admission (10%) and end-stage disease (8%). Effective triaging improved prioritisation of urgent cases, reduced unnecessary echocardiograms and enabled resource reallocation. If all inappropriate referrals were rejected as per BSE guidelines, approximately 680 inpatient echoes could be avoided annually — equivalent to saving 2 echoes per day with an estimated financial benefit of £22,500 per year.

**Conclusion** Implementing BSE triage guidelines significantly enhanced clinical efficiency and resource utilisation. These findings support wider integration of BSE triaging principles into electronic health record systems to improve inpatient echocardiography services, particularly in high-demand tertiary centres. Embedding triage pathways alongside targeted referrer education will sustain appropriate referrals and maximise clinical and financial benefits.

## ABS024 Retrospective observational study of incidence, prevalence and clinical management of hypertrophic cardiomyopathy

Eaint Kay Khine Thein^1^, Farid Ebrahimjee^1^, Alexander Hanshall^1^, Rajaperumal Ravi^1^, Okkar Myint Zaw^1^, Robert Ambrogetti^1^, Rodney DePalma^1^

^1^Wycombe Hospital, Buckinghamshire Healthcare NHS Foundation Trust, High Wycombe, UK

*Echo Research & Practice 2026*, **13(Suppl 1):**ABS024

**Introduction** Hypertrophic cardiomyopathy (HCM) is a genetic heart condition characterized by the thickening of the heart muscle, which can impede normal blood flow and lead to various cardiac complications. In the United Kingdom, HCM affects approximately 1 in every 500 individuals. The incidence and prevalence of diagnosis and follow-up is not well understood in the Buckinghamshire region. This study looked at data including patient symptoms and echocardiogram findings from the last two years to determine the incidence, prevalence and clinical management of HCM in Wycombe Hospital.

**Methods** We conducted a retrospective analysis using a local database of echocardiograms and clinical correspondence within the Evolve system to determine the incidence of hypertrophic cardiomyopathy (HCM) over the past two years. Echocardiographic studies were assessed based on the British Society of Echocardiography (BSE) guidelines, specifically applying the diagnostic threshold of ≥15mm maximal left ventricular (LV) wall thickness in one or more myocardial segments as an initial screening criterion for HCM. Patients meeting this criterion were further evaluated through clinical follow-up data to determine the symptoms, family history, electrocardiogram (ECG) changes, other BSE recommended echocardiography minimum dataset. Additionally, these patients were reviewed as to whether they subsequently underwent Cardiac MRI, the gold standard for confirming the diagnosis of HCM as per BSE and ESC recommendations. After the diagnosis of HCM is confirmed, we thereafter studied these patients in view of the surveillance, genetic testing, hypertrophic cardiomyopathy sudden cardiac death (HCM-SCD) risk score and the decision-making regarding implantable cardiac defibrillator (ICD).

**Results** Preliminary results showed that 52 patients had a wall thickness of >15mm. Only 22 of these patients went on to have follow-up with Cardiac MRI. Most patients (31 out of 52) had the result of a clinical diagnosis of hypertrophic cardiomyopathy (HCM). Out of which, only 18 patients had cardiac MRI. Other patients from the study had the clinical diagnosis of hypertensive heart disease, aortic stenosis and cardiac amyloidosis, with 2 patients in each category. The rest of the patients are awaiting clinical appointment or further investigations to identify the cause of left ventricular hypertrophy.

**Discussion** These findings suggest that a subset of patients may remain undiagnosed with HCM, highlighting the importance of comprehensive follow-up evaluations, including cardiac MRI, to better assess its incidence in this population. To enhance clinical management, we plan to educate local clinicians on HCM guidelines and the BSE HCM minimum dataset, ensuring more accurate diagnosis and optimal patient care.

## ABS025 Reduction of early left ventricular systolic function in patients with positive stress echocardiography – an exploratory analysis in the EVAREST study

Yujia Yang^1,2^, Casey Johnson^3^, Samuel Krasner^3^, Phil Chowienczyk^1,2^, Manish Sinha^1,2^, Paul Leeson^3,^Haotian Gu^1,2^

^1^King’s College London, UK, ^2^Guy’s and St Thomas’ NHS Foundation Trust, London, UK, ^3^The University of Oxford, UK

*Echo Research & Practice 2026*, **13(Suppl 1):**ABS025

**Background** Ischaemic heart disease (IHD) remains a leading cause of morbidity and mortality in the UK. Stress echocardiography (SE) is the first-line diagnostic tool for IHD. First-phase ejection fraction (EF1) and first-phase global longitudinal strain (GLS1) are novel measures of early left ventricular systolic function and may be reduced in patients with IHD.

**Purpose** To perform an exploratory analysis of resting EF1 and GLS1 in a subsample of participants from the EVAREST study, a UK-wide observational SE study.

**Methods** Resting EF1 and GLS1 were retrospectively measured in 106 patients. EF1 was defined as the volume change, and GLS1 as myocardial longitudinal deformation, up to the time of maximal aortic valve velocity. Patients were categorized based on clinically reported SE results (positive: n=51; negative: n=55).

**Results** Patients with positive SE were heavier (Table 9) and had significantly greater left ventricular mass (Table 10). Resting GLS, EF1 and GLS1 were significantly lower in patients with positive SE results compared to those with negative results (GLS: -16.53 ± 2.45% vs. -18.31 ± 3.16%, p=0.003; EF1: 18.12 ± 7.81% vs. 25.63 ± 7.23%, p<0.001; GLS1: –4.23 ± 2.09% vs. –5.52 ± 1.87%, p=0.002). These differences remained statistically significant after adjustment for body mass index, left ventricular mass, and EF. EF1 demonstrated the highest diagnostic accuracy for identifying SE positivity (Area Under the Curve =0.769), outperforming other systolic function measures (Fig. 1).

**Conclusion** GLS, EF1 and GLS1 are reduced at rest in patients with positive SE despite preserved EF, suggesting early systolic dysfunction. These novel markers may be useful in detecting early ischaemia but require validation in a larger cohort.

**Table 9:** General characteristics between patients with negative and positive SE results

Measure	SE negative (n=55)	SE positive (n=51)	P value
Age (Years)	68.33 ± 9.27	64.57 ± 10.40	0.053
Male Sex (n, %)	33(60%)	30(59%)	0.608
Height (m)	1.68 ± 0.09	1.71 ± 0.10	0.228
Weight (kg)	74.40 ± 14.84	86.15 ± 18.37	0.002
BMI (kg/m^2^)	26.15 ± 4.44	29.18 ± 5.41	0.006
HR (bpm)	70.40 ± 11.48	71.71 ± 11.04	0.552
SBP (mmHg)	138.5 ± 18.31	140.0 ± 18.17	0.678
DBP (mmHg)	79.41 ± 15.86	74.16 ± 12.56	0.065

BMI = body mass index; HR = Heart Rate; SBP = Systolic Blood Pressure; DBP = Diastolic Blood Pressure

**Table 10:** Echocardiographic measures between patients with negative and positive SE results

Measure	SE negative (n=55)	SE positive (n=51)	P value
LVM(g)	145.34 ± 63.10	176.04 ± 55.02	0.009
LVMI(g/m^2^)	79.01 ± 34.36	90.81 ± 28.42	0.087
EF (%)	62.15 ±5.91	61.18 ± 5.92	0.399
EF1 (%)	25.63 ± 7.23	18.12 ± 7.81	<0.001
GLS (%)	-18.31 ± 3.16	-16.53 ±2.45	0.003
GLS1 (%)	-5.52 ± 1.87	-4.23 ± 2.09	0.002
TPAVF/TRR ratio	0.16 ± 0.04	0.15 ± 0.04	0.182

LVM = Left Ventricular Mass; LVMI = Left Ventricular Mass Index; EF = Ejection Fraction; EF1 = First phase Ejection Fraction; GLS = Global Longitudinal Strain; GLS1 = First phase Global Longitudinal Strain; TPAVF = Time to Peak Aortic Velocity Flow Velocity; TRR = R-R interval

**Fig. 1(abstract ABS025):**
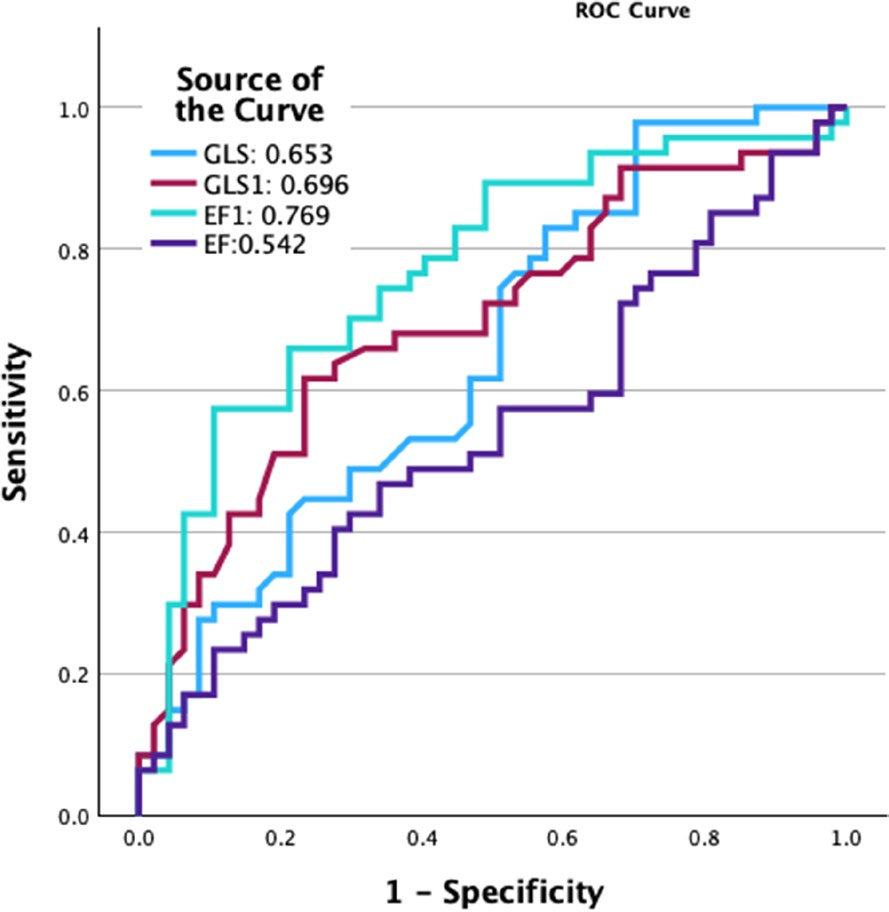
ROC analysis

## ABS026 Physiologist-led triaging of transthoracic echocardiogram requests for infective endocarditis

Niall Carlin ^1^

^1^Royal Infirmary of Edinburgh, UK

*Echo Research & Practice 2026*, **13(Suppl 1):**ABS026

**Introduction** Infective endocarditis (IE) is a serious infection of the heart’s endocardium, primarily affecting heart valves and caused by microorganisms in the bloodstream forming infected vegetations. A transthoracic echocardiogram (TTE) is the first-line imaging modality for suspected IE. Noticing an increase in *‘query IE’* referrals, a retrospective audit was conducted over three months (January to March) from 2022 to 2025 at the Royal Infirmary of Edinburgh. NHS Lothian’s local protocol for IE diagnosis follows a modified Duke criterion.

**Objective** This audit aimed to evaluate the number of TTE requests for suspected IE and assess adherence to local protocol, as well as determine if physiologist-led triaging affected the number of confirmed IE cases.

**Method** A patient data, results and echocardiography system were reviewed for all patients referred for *‘query IE’* TTEs between January and March from 2022 to 2025. In July 2023, the ECHO department implemented a stricter policy enforcing local protocol for TTE requests.

**Results**

**Fig. 1(abstract ABS026):**
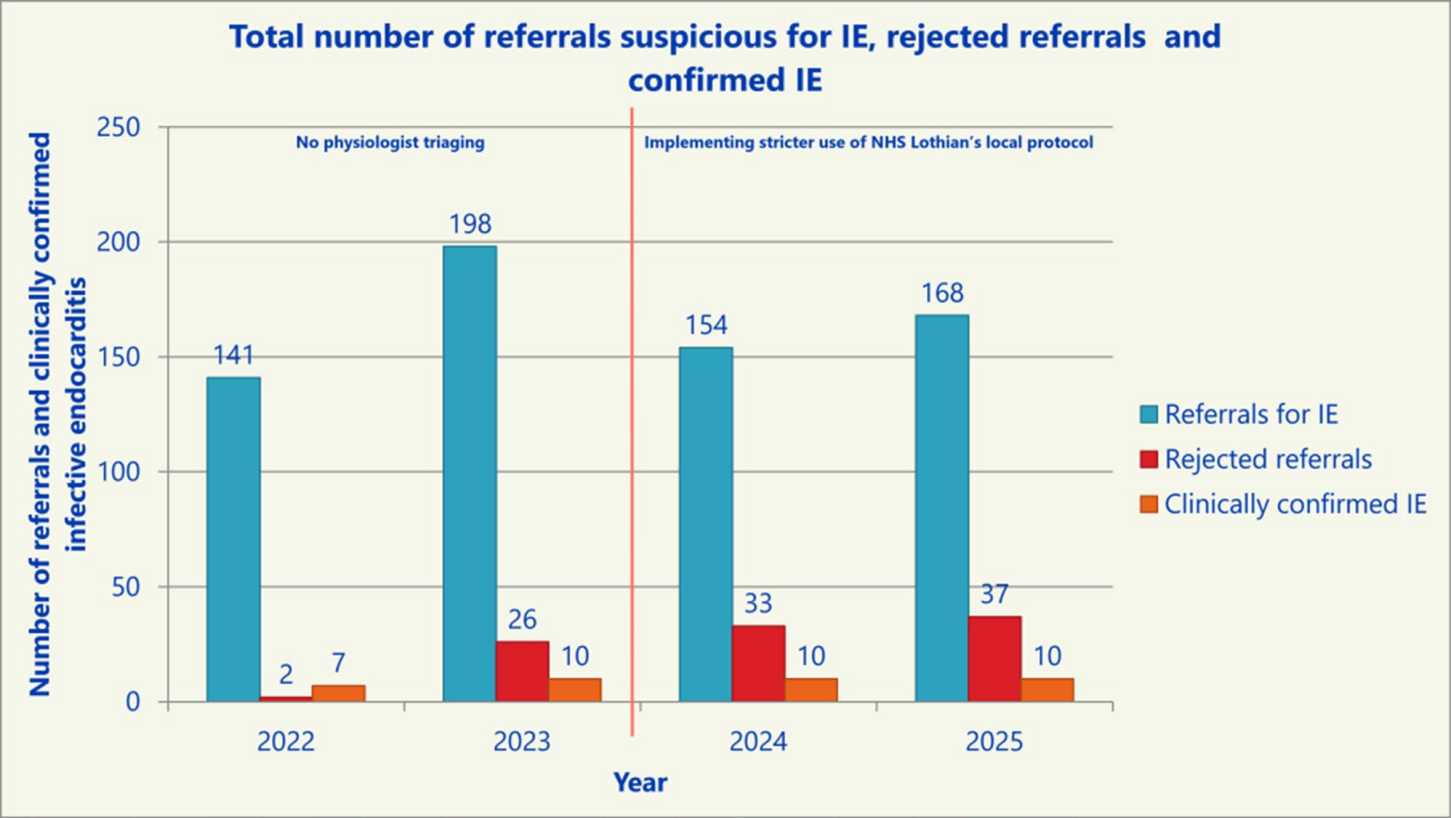
Total number of referrals suspicious for IE, rejected referrals and confirmed IE

**Fig. 2(abstract ABS026):**
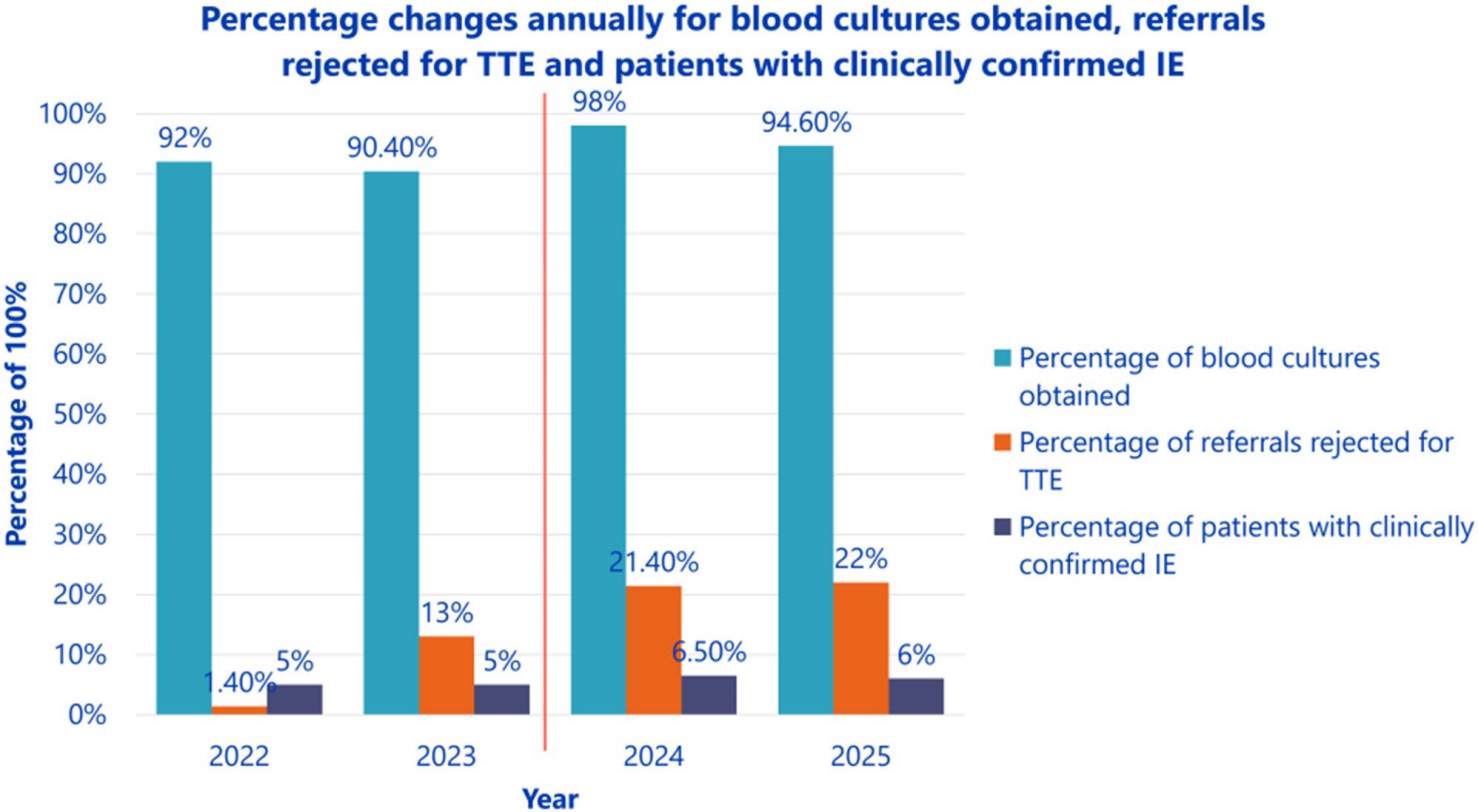
Percentage changes annually for blood cultures obtained, referrals rejected for TTE and patients with clinically confirmed IE

**Conclusion** Following protocol enforcement, inpatient TTE requests for suspected IE decreased by 15.1% from 2023 to 2025, while the proportion of confirmed IE cases rose from 5% to 6%. Additionally, blood culture rates increased from 90.4% (2023) to 98% (2024), underscoring the importance of positive blood cultures before TTE referral.

## ABS027 The right heart of the elite cyclist: A comparative study of male and female sprint and Endurance athletes

Max Knights^1^, Aneil Malhotra^2^, Robert Cooper^3^, Shaun Robinson^3,4^, Tristan Ramacharan^5^, Joseph Maxwell^3^, Jerusalem Fekadu^6^, Camille Galloway^7^Keith George^3^, Nigel Jones^8^and David Oxborough^3^

^1^Salford Royal NHS Foundation Trust, UK, ^2^Institute of Sport, Manchester Metropolitan University, UK, ^3^Research Institute for Sport and Exercise Sciences, Liverpool John Moores University, UK, ^4^Imperial College Healthcare NHS Trust, London, UK, ^5^Heart Unit, Birmingham Women’s and Children’s NHS Foundation Trust, UK, ^6^Guy’s and St Thomas’ NHS Foundation Trust, London, UK, ^7^School of Health and Exercise Sciences, University of British Columbia Okanagan, Kelowna, Canada, ^8^Medical Department, British Cycling, Manchester, UK

*Echo Research & Practice 2026*, **13(Suppl 1):**ABS027

**Background/introduction** Olympic level cycling involves various sub-specialities including pure endurance and pure sprint disciplines with the associated differences in isometric and isotonic training components. In view of this, the elite cyclist serves as the ideal model to study training specific right heart variances within the same elite sporting discipline.

**Purpose** The aims of the study are two-fold: i) to establish physiological structural and functional differences in the right ventricle (RV) and right atrium (RA) in elite endurance and sprint cyclists, and ii) to determine discipline specific differences between male and female elite cyclists.

**Methods** One hundred and eighty-six (110 males and 76 females) elite international level cyclists (mean age 23 ± 5) were recruited. Cyclists were grouped by discipline (endurance and sprint) and sex (male and female). Group-specific training volumes (endurance, sprint and strength) were reported and metabolic equivalent test (MET) hours per week were subsequently calculated. All cyclists were evaluated by 2D, TDI and strain echocardiography with a focus on RV and RA structure and function. A two-way ANOVA with Tukey Post-Hoc tests were used to compare between groups.

**Results** There were no significant differences between any groups for overall training hours per week. There was a significant main effect of both sex and discipline on RV and RA structure with larger absolute and scaled values seen in male and endurance cyclists than female and sprint cyclists (Fig. 1) (*P<*0.05). Endurance MET hours per week were significantly correlated to RV and RA structural dimensions (Fig. 2) (*P*<0.05). There were significant main effects of sex and discipline on RV function but no specific group differences observed following post-hoc testing (Fig. 3). There were no significant correlations of RV function to training specific MET hours per week.

**Conclusions** Endurance cycling training induces greater right heart structural remodelling than sprint training in elite cyclists. Sex and discipline are both strong and independent influences on RV and RA structural adaptation in elite cyclists, whilst endurance and sprint training do not elicit significantly different RV and RA functional adaptations in elite cyclists.

**Fig. 1(abstract ABS027):**
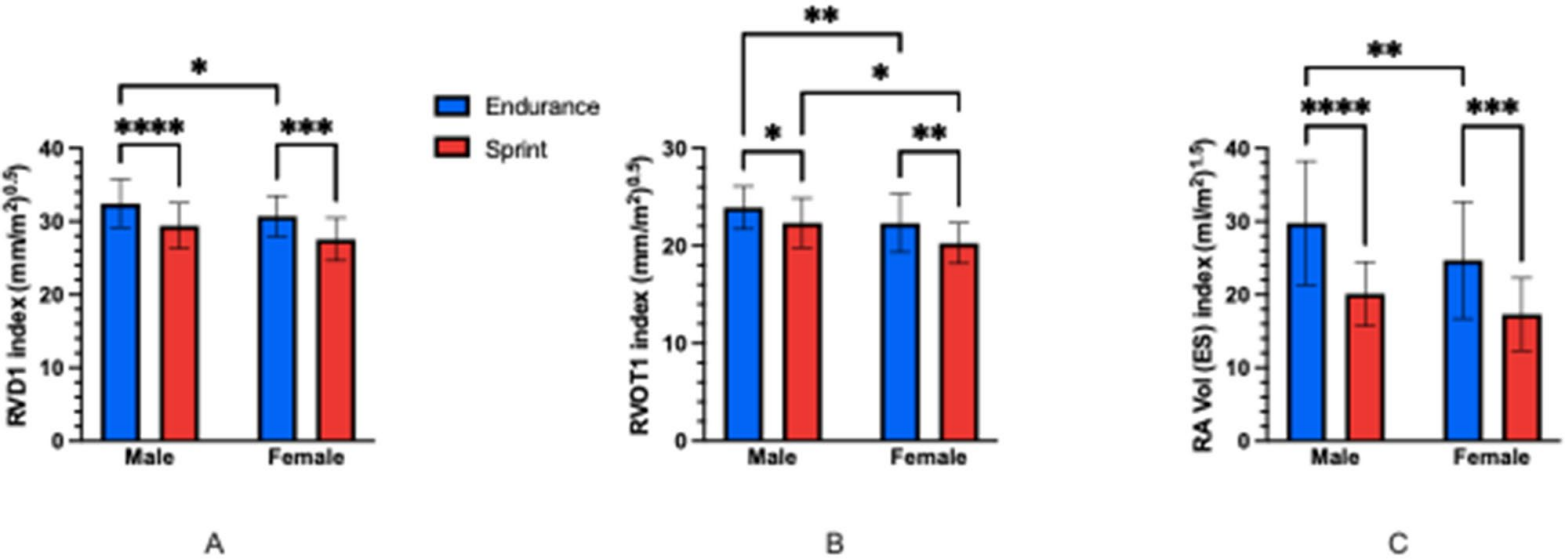
Comparison of RV and RA structural parameters between groups A) RVD_1_ index B) RVOT_1_ index C) RA Vol (ES) index

**Fig. 2(abstract ABS027):**
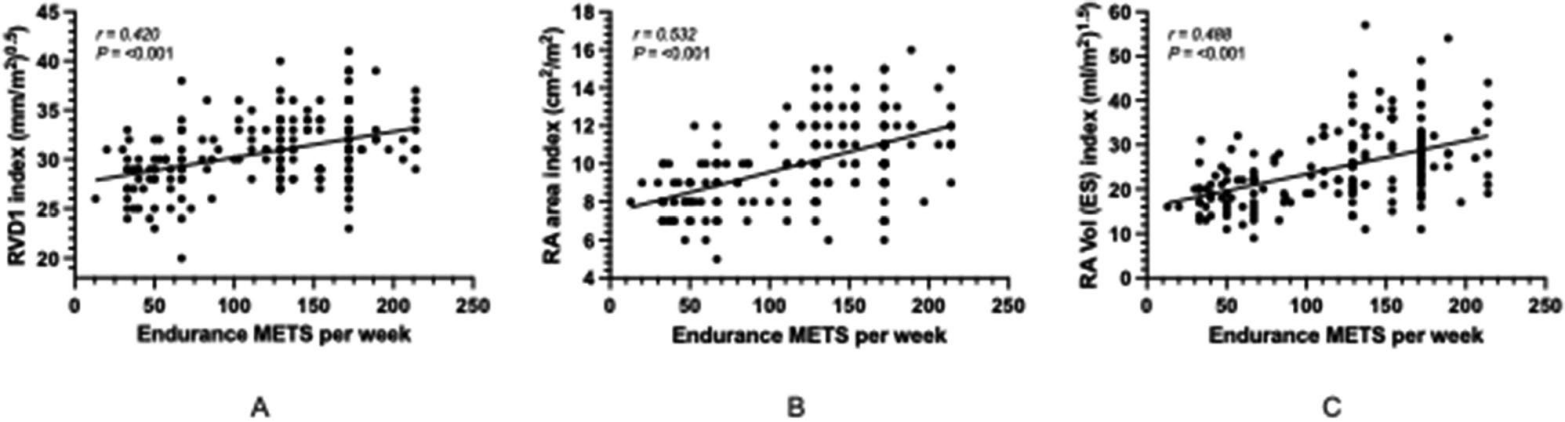
Correlations of endurance METS to RV and RA structure. A) RVD_1_ index B) RA area index C) RA Vol (ES) index

**Fig. 3(abstract ABS027):**
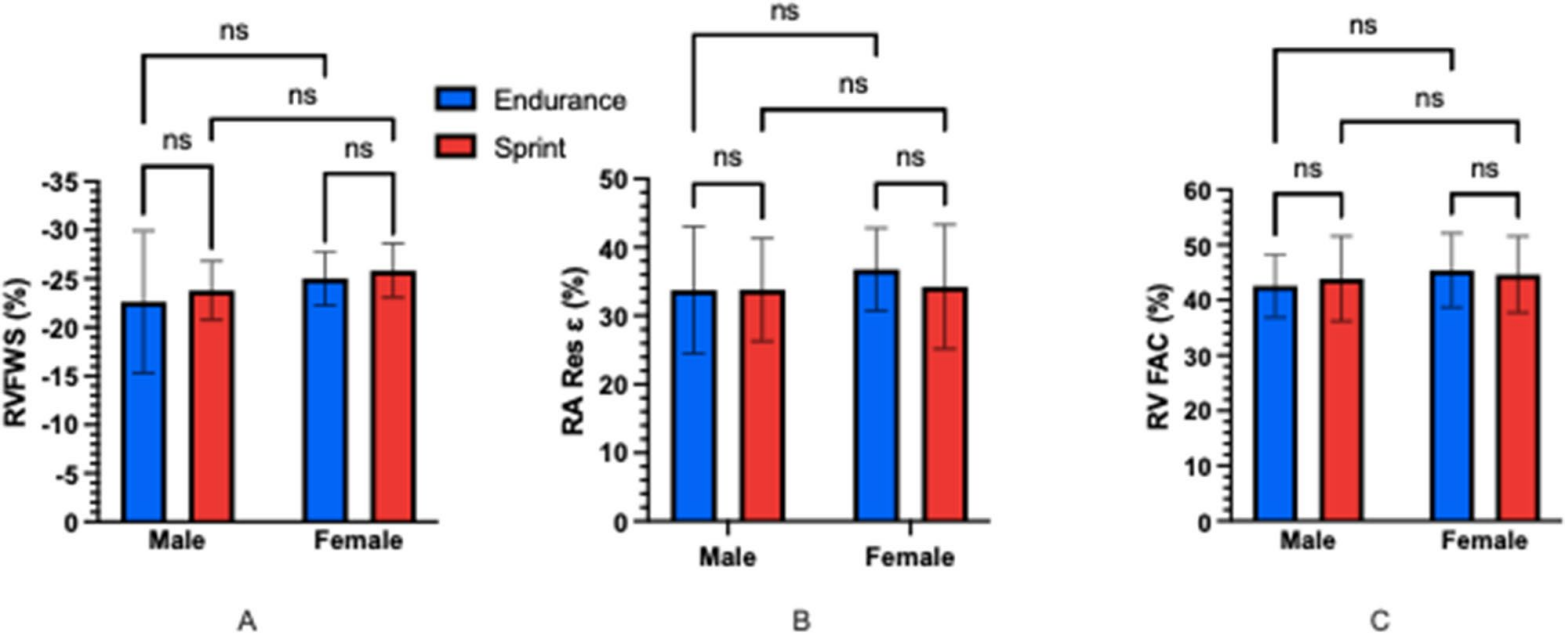
Comparison of RV and RA functional parameters between groups A) RVFWS B) RA Res ɛ C) RV FAC. Abbreviations: RVFWS, right ventricular free wall strain; RA Res ɛ, right atrial reservoir strain; RV FAC, right ventricular fractional area change; RVD1 right ventricular basal diameter; RA Vol (ES), right atrial end systolic volume

## ABS031 Feasibility, reproducibility, and accuracy of three-dimensional knowledge-based reconstruction for right ventricular assessment in adults with repaired tetralogy of fallot

Dario Freitas^1,2^, Cristina Almeida^3^, Haotian Gu^4^, Saleha Kabir^5^, Elena Surkova^6,7^, Shakeel Qureshi^5^, John Simpson^5^, Alessandra Frigiola^1,4^, Antonio F. Corno^2^, Rehan T. Junejo^2^, Camelia Demetrescu^1^, Yaso Emmanuel^1^

^1^Guy’s and St Thomas’ Hospital NHS Foundation Trust, London, UK, ^2^Department of Life Sciences, Manchester Metropolitan University, UK, ^3^The Hospital for Sick Children, Toronto, Canada, ^4^Institute of Cardiovascular Imaging Science, King’s College London, UK, ^5^Evelina London Children’s Hospital, Guy’s and St Thomas’ NHS Foundation Trust, UK, ^6^Royal Brompton and Harefield Hospitals, Guy’s and St Thomas’ NHS Foundation Trust, London, UK, ^7^National Heart and Lung Institute, Imperial College, London, UK

*Echo Research & Practice 2026*, **13(Suppl 1):**ABS031

**Background** Accurate and reproducible assessments of right ventricle (RV) morphology and function are critical in adults with repaired tetralogy of fallot (rTOF). Cardiac magnetic resonance (CMR) is the gold standard; however, accessibility limitations support the need for alternative imaging modalities. This study evaluates feasibility, reproducibility and accuracy of three-dimensional knowledge-based reconstruction (3D-KBR) for longitudinal RV monitoring.

**Methods** 34 asymptomatic rTOF patients (32±11 years) with moderate-severe pulmonary regurgitation and 22 healthy controls (35±5 years) underwent echocardiography and CMR in a single-centre prospective longitudinal study (rTOF follow-up interval average: 255 days). RV volumes and ejection fraction (EF) were quantified using 3D and 3D-KBR echocardiography and compared to assess accuracy. Additionally, Intra- and inter-observer reproducibility for 3D-KBR were blindly assessed by two operators.

**Results** Compared with controls, rTOF patients had significantly increased RV volumes and reduced function across all imaging modalities (Table 11) with high feasibility (85-94%). Importantly, while standard 3D values differed particularly from CMR (EDV *r*= 0.91, p<0.001; ESV *r*= 0.79, p<0.001; EF *r*= 0.33, p=0.096), 3D-KBR data was within 10% for both operators and more strongly correlated (EDV *r*= 0.95, p<0.001; ESV *r*= 0.91, p<0.001; EF *r*= 0.64, p<0.001) (Fig. 1). 3D-KBR demonstrated excellent intra- and inter-observer reproducibility which slightly declined in rTOF patients during follow-up (Table 12).

**Conclusion** Despite minor differences in volumes and EF, 3D-KBR offers a valuable alternative to CMR for interim surveillance and support clinical management in rTOF patients. Additionally, 3D-KBR appears to be a feasible and reproducible technique for longitudinal RV assessment in these patients.

**Table 11:** Echocardiographic and CMR parameters of RV size and systolic function in both groups

Parameter	Control group	rTOF group	Mean Difference [95% CI] (rTOF group – Control group)	*p* **value**
**Baseline Visit**				
Image quality (sub-optimal)	2 (10.5%)	17 (53.1%)		
**Echocardiography 2D assessment (operator 1)**	**(n = 22)**	**(n = 34)**		
Basal diameter, mm	41.00 ± 4.49	49.09 ± 6.10	8.09 (4.86, 11.33)	<0.001
Mid diameter, mm	37.00 ± 5.95	50.22 ± 8.86	13.22 (8.61, 17.83)	<0.001
Length, mm	84.37 ± 6.83	92.03 ± 7.97	7.66 (3.26, 12.07)	<0.001
TAPSE, mm	22.26 ± 2.60	18.45 ± 3.64	-3.81 (-5.73, -1.89)	<0.001
S′, cm/s	14.05 ± 1.81	11.03 ± 2.28	-3.01 (-4.25, -1.78)	<0.001
EDA, cm^2^	26.18 ± 5.45	37.43 ± 8.45	11.25 (6.89, 15.61)	<0.001
ESA, cm^2^	14.65 ± 3.40	21.32 ± 5.76	6.67 (3.74, 9.60)	<0.001
FAC, %	44.28 ± 4.21	43.35 ± 5.61	-0.93 (-3.92, 2.06)	0.535
Free wall LS basal, %	−27.08 ± 4.42	−26.09 ± 5.32	0.99 (-1.93, 3.91)	0.498
Free wall LS mid, %	−23.58 ± 3.34	−21.41 ± 4.77	2.17 (-0.33, 4.67)	0.087
Free wall LS apical, %	−25.39 ± 5.21	−19.56 ± 5.75	5.83 (2.59, 9.06)	<0.001
Free wall Apical 4-chamber LS, %	−25.59 ± 2.92	−22.36 ± 3.94	3.23 (1.14, 5.33)	0.003
Global 4-chamber LS, %	−21.99 ± 2.63	−19.57 ± 3.36	2.42 (0.61, 4.23)	0.010
**Echocardiography 3D assessment (operator 1)**	**(n = 22)**	(n = 27***)		
EDV, mL	131.75 ± 31.95	181.22 ± 50.45	49.47 (23.01, 75.93)	<0.001
ESV, mL	61.12 ± 16.30	92.28 ± 27.61	31.16 (16.88, 45.43)	<0.001
EF, %	53.72 ± 4.03	49.14 ± 5.81	-4.58 (-7.69, 1.47)	0.005
**Echocardiography 3D KBR assessment (operator 1)**	(n = 19*)	(n = 32**)		
EDV, mL	168.47 ± 49.9	226.53 ± 64.10	58.06 (23.54, 92.56)	<0.001
ESV, mL	81.71 ± 27.12	117.91 ± 35.59	36.20 (17.15, 55.25)	<0.001
EF, %	51.47 ± 6.23	48.08 ± 5.38	-3.38 (-6.70, -0.05)	0.005
**Echocardiography 3D KBR assessment (operator 2)**	(n = 19*)	(n = 32**)		
EDV, mL	174.86 ± 48.96	227.57 ± 72.40	52.71 (15.01, 90.41)	0.007
ESV, mL	88.31 ± 26.11	122.13 ± 41.99	33.82 (12.31, 55.33)	0.003
EF, %	49.53 ± 4.31	46.73 ± 6.09	-3.10 (-6.30, -0.11)	0.058
**CMR assessment**		**(n = 34)**		
EDV, mL		235.85 ± 66.53		
ESV, mL		115.21 ± 38.87		
EF, %		51.76 ± 6.71		

Data is presented as mean ± SD or *n* (%). *3 healthy volunteers were excluded from a total of 22. **2 patients were excluded from a total of 34. ***7 patients were excluded from a total of 34

*p* value of < or = 0.005 is considered of significance

TAPSE, tricuspid annular plane systolic excursion; EDA, end-diastolic area; EDV, end-diastolic volume; ESA, end-systolic area; ESV, end-systolic volume; LS, longitudinal strain; FAC, fractional area change; EF, ejection fraction; 3D KBR, three-dimensional knowledge-based reconstruction; CMR, cardiac magnetic resonance

**Table 12:** Reproducibility of 3D KBR method between operator 1 and 2 to assess RV volumes and EF in the rTOF group

Inter-observer variability	rTOF group			
Baseline Visit (n = 32*)				
**Echocardiography 3D KBR operator 1 vs. 3D KBR operator 2**	**ICC (95% CI)**	**Coefficient of variation (%)**	**Mean bias ± SD**	***p*** **value**
EDV, mL	0.966 (0.931, 0.984)	10.85	-1.04 ± 24.63	0.812
ESV, mL	0.943 (0.884, 0.972)	15.02	-4.22 ± 18.03	0.195
EF, %	0.659 (0.302, 0.834)	12.27	1.66 ± 5.80	0.116
Follow-up Visit (n = 19**)				
**Echocardiography 3D KBR operator 1 vs. 3D KBR operator 2**	**ICC (95% CI)**	**Coefficient of variation (%)**	**Mean bias ± SD**	***p*** **value**
EDV, mL	0.979 (0.946, 0.992)	9.37	-8.68 ± 20.90	0.087
ESV, mL	0.986 (0.964, 0.995)	8.12	-6.41 ± 9.97	0.012
EF, %	0.903 (0.748, 0.963)	5.62	0.97 ± 2.54	0.111

Data is presented as mean ± SD or *n* (%)

*p* value of < or = 0.005 is considered of significance

*2 patients were excluded from a total of 34. *19 patients had a follow-up visit in the clinic. The average time between visits was 255 days (IQR: 68). EDV, end-diastolic volume; ESV, end-systolic volume; EF, ejection fraction; 3D KBR, three-dimensional knowledge-based reconstruction

**Fig. 1(abstract ABS031):**
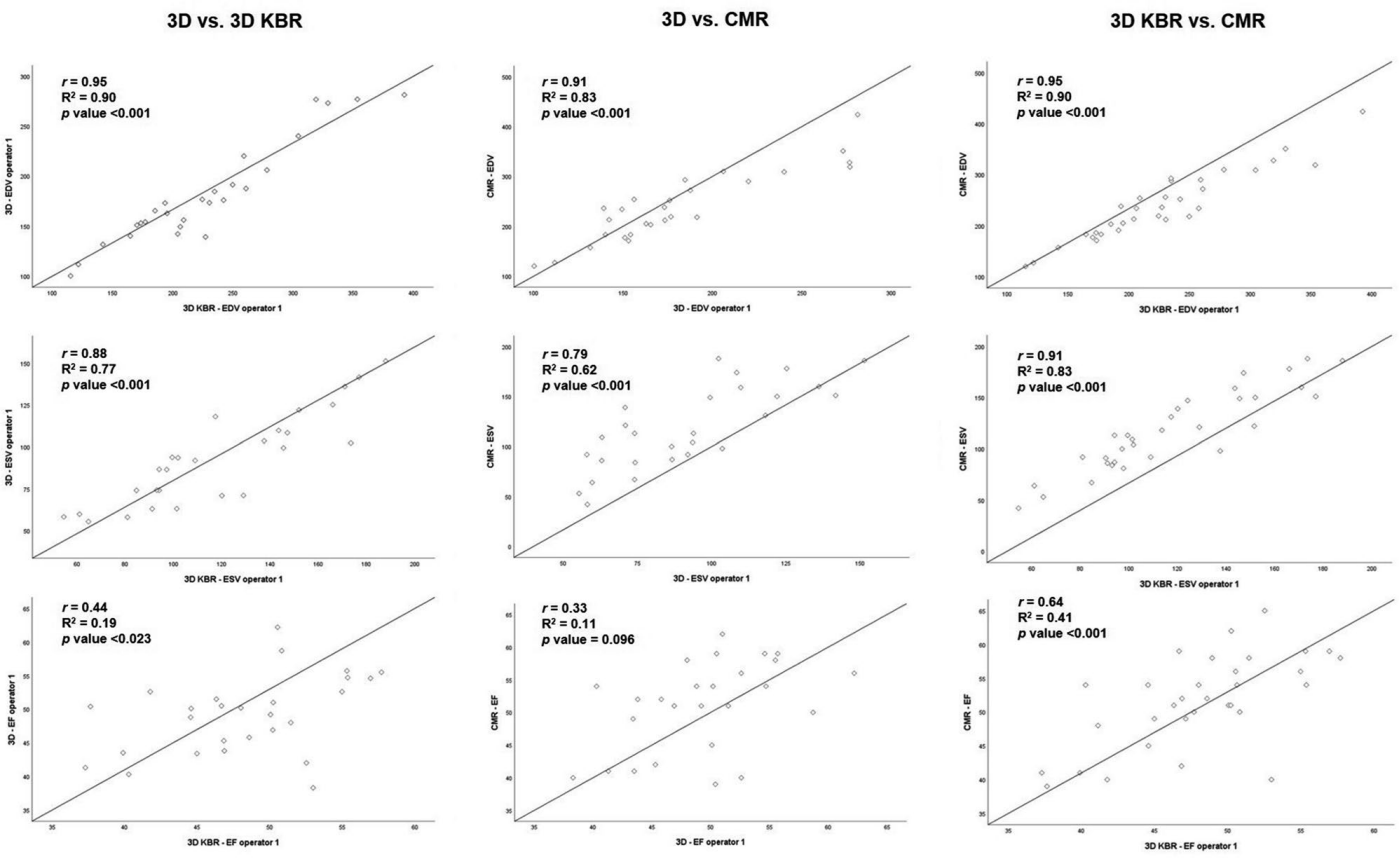
Inter-method agreement for RV volume and EF measurements in rTOF patients (operator 1, visit 1), comparing 3D vs. 3D KBR, 3D vs. CMR, and 3D KBR vs. CMR

## ABS032 Temporal variability and heterogeneity of Doppler profiles in mid-cavity obstructive hypertrophic cardiomyopathy

Will Davies^1^, Tim Husselbury^1^, Guido Mezzanotte^1^, Konstantinos Moschonas^1^, Massimiliano Lorenzini^1^, Constantinos O’Mahony^1^, Saidi Mohiddin^1^, James Malcolmson^1^

^1^St Bartholomew’s Hospital, London, UK

*Echo Research & Practice 2026*, **13(Suppl 1):**ABS032

**Background** Left ventricular mid-cavity obstruction (LVMCO) is a less common hypertrophic cardiomyopathy (HCM) variant. Doppler echocardiography can identify LVMCO but often underestimates magnitude. Detection of LVMCO and associated LV morphological abnormalities have therapeutic implications.

**Purpose** To evaluate LVMCO Doppler profiles’ temporal changes.

**Methods** Retrospective longitudinal study involving serial transthoracic echocardiograms (TTE). Doppler profiles were categorised as (1) non-obstructive flow acceleration, (2) early obstructive prolonged (holo-systolic) apical emptying, (3) overt obstructive LVMCO Doppler signal void with distinct paradoxical jet or (4) overt obstructive high-velocity complete spectral jet (>30 mmHg) (Fig. 1).

**Results** A total of 232 LVMCO patients (mean age 64.0 (SD 13) years, 28% female, median follow-up 8 (IQR 3) years) were classified as having a discrete apical chamber (DAC) in 43% or overt LV apical aneurysm (LVAA) (57%). In the DAC group, index and latest Doppler profiles demonstrated overt LVMCO (category 3 or 4) in 77% and 91% respectively. In the LVAA group, index and latest Doppler profiles demonstrated overt LVMCO (category 3 or 4) in 95% and 98% respectively, with 89 and 93% showing Doppler signal void and paradoxical diastolic jet (category 3) at index and latest TTE. New overt LVMCO developed in 29 (13%) patients (95% had cavity obliteration without baseline DAC/LVAA). Overt LVMCO became unobstructive in 11 (5%) patients (45% had a drop in LVEF to <60%) (Fig. 2).

**Conclusions** Doppler signal void with paradoxical jet is the commonest LVMCO Doppler profile irrespective of apical LV morphology. LVMCO Doppler profiles are dynamic, progressive and categorisation vary over time.

**Abbreviations:** HCM, hypertrophic cardiomyopathy; LVMCO, left ventricular mid-cavity obstruction; DAC, discrete apical chamber; LVAA, left ventricular apical aneurysm.

**Fig. 1(abstract ABS032):**
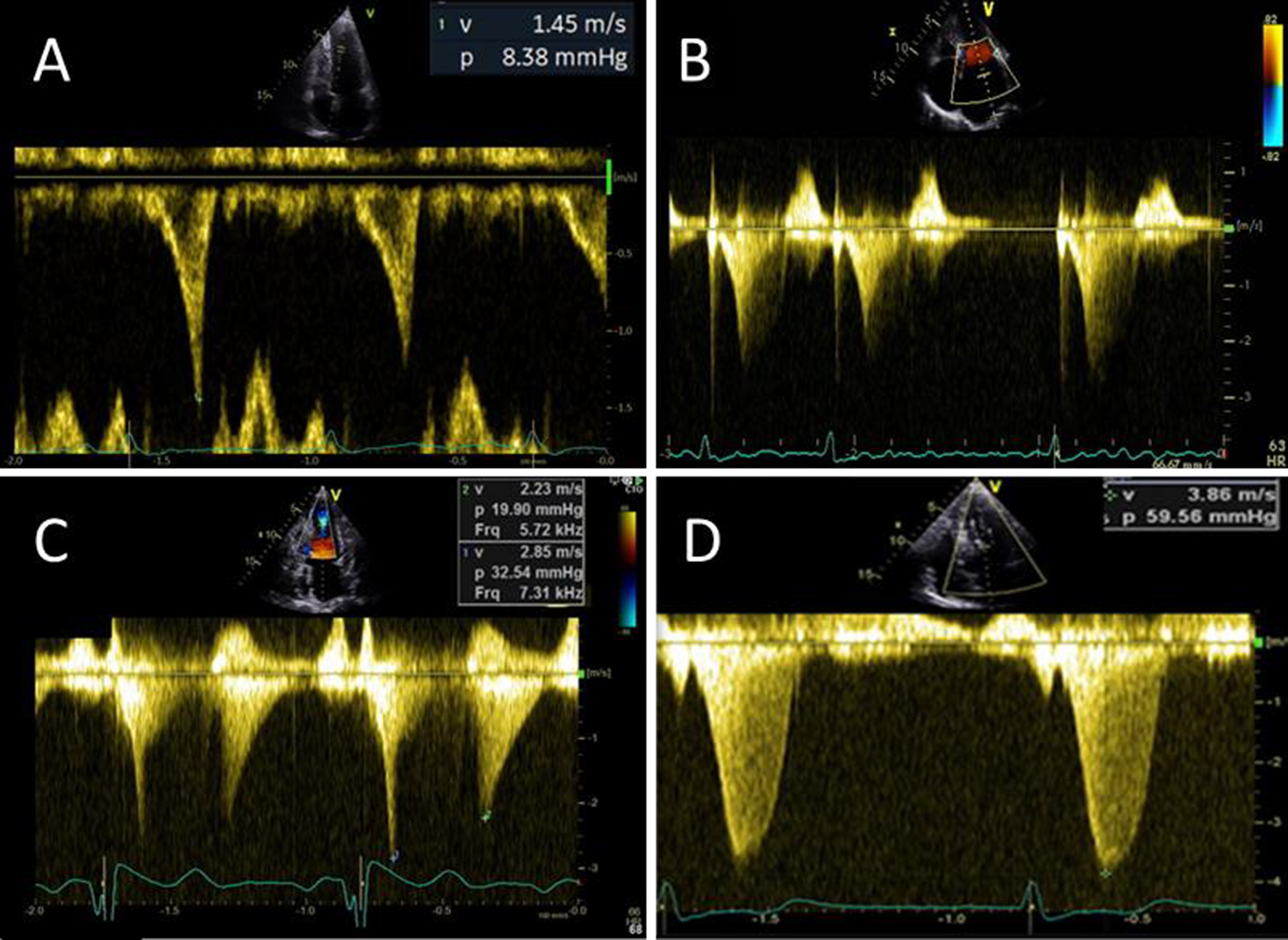
TTE images displaying four distinct Doppler profiles: Category 1 non-obstructive flow acceleration defined as either early or mid-systolic peak without being holo-systolic (A); Category 2 early-obstructive prolonged apical emptying defined as persisting throughout systole and <30 mmHg (B); Category 3 overt obstructive Doppler signal void with paradoxical jet defined as two distinct jets in systole and paradoxical diastolic jet of any gradient (C); and Category 4 high velocity complete spectral jet defined as holo-systolic and >30 mmHg (D). TTE, transthoracic echocardiography; LVMCO, left ventricular mid-cavity obstruction

**Fig. 2(abstract ABS032):**
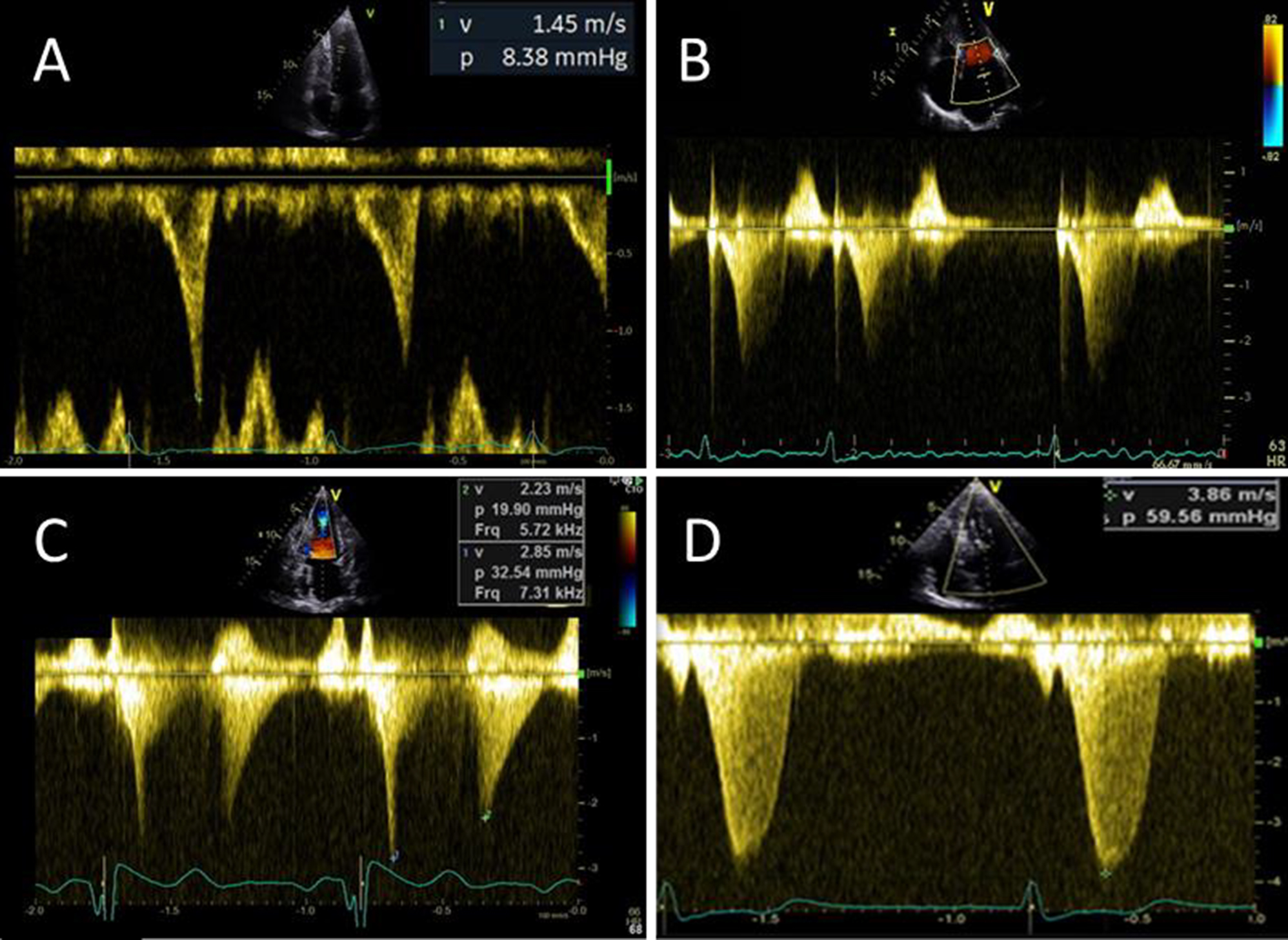
Whole cohort Doppler profile category tracker across index and latest TTE. Blue ovals show the absolute quantity of patients moving from one category to another and orange squares show the percentage of patients in each category at index and latest TTE. Cat, Category; TTE, transthoracic echocardiogram

**Fig. 3(abstract ABS032):**
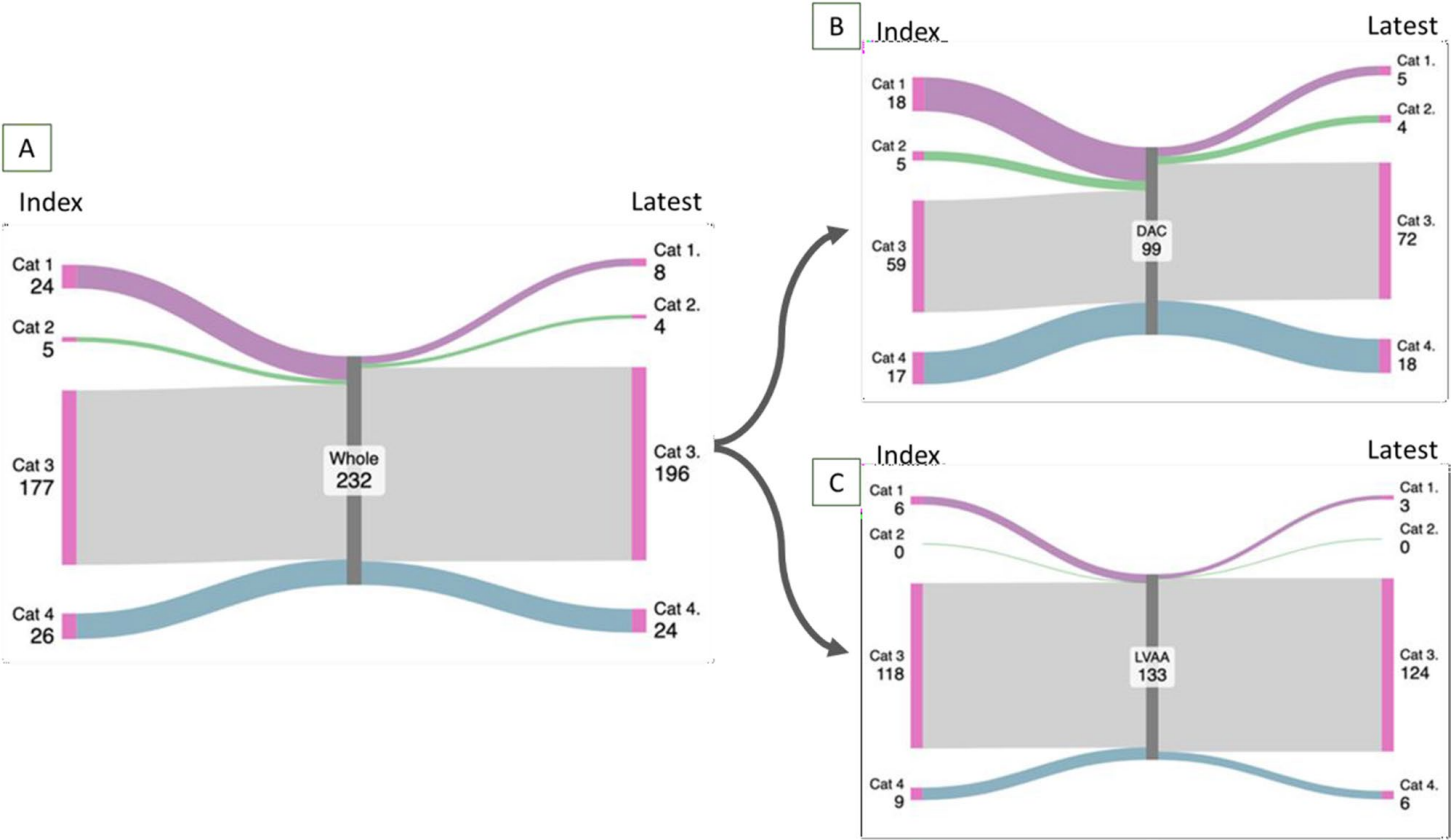
Sankey diagrams highlighting absolute numbers of patients in each Doppler profile category at point of index and latest TTE. A: Whole cohort, B: DAC, and C: LVAA. Cat, Category; TTE, transthoracic echocardiogram; DAC, discrete apical chamber; LVAA, left ventricular apical aneurysm

## ABS033 Echocardiography guided permanent pacemaker optimisation for symptomatic obstructive hypertrophic cardiomyopathy

Jannah Ahmad^1^, Will Davies^1^, Tim Husselbury^1^, Guido Mezzanotte^1^, Saidi Mohiddin^1^, James Malcolmson^1^

^1^St Bartholomew’s Hospital, London, UK

*Echo Research & Practice 2026*, **13(Suppl 1):**ABS033

**Introduction** Pacing-induced contractile dyssynchrony can reduce left ventricular (LV) outflow tract obstruction (LVOTO) in hypertrophic cardiomyopathy (HCM) patients. Echocardiography guided optimisation of atrioventricular (AV) delays is essential to avoid truncation of LV filling. Long-term efficacy data of this technique is sparse.

**Purpose** To evaluate the effectiveness of pacemaker optimisation in reducing LVOTO and alleviating symptoms in HCM patients.

**Methods** A retrospective, single-centre cohort study including HCM patients with isolated LVOTO referred for echocardiography-guided pacing optimisation of existing dual chamber permanent pacemaker (PPM) devices. Optimal AV delay was selected based on maximal QRS duration without truncation of LV filling on Pulsed wave Doppler echocardiography. Maximal LVOTO gradients (resting or Valsalva) and reported New York Heart Association (NYHA) functional class were recorded at baseline and 1-year post-optimisation.

**Results** Between July ‘17 and June ‘24, 75 patients (60±15 years, 48% female) underwent optimisation. Atrial sensing ventricular pacing was used in all patients. Mean programmed sensed AV delay was 102±31ms. Pacing acutely reduced maximal LVOTO gradients from 77±34 mmHg to 59±36 mmHg, representing a mean reduction of 19±20 mmHg (p<0.0001) (Fig. 1). In the 53 patients with 1-year follow-up data available, further gradient reductions were observed to 50±30 mmHg (p=0.04) (Fig. 2). Median NYHA class at baseline was 2 (IQR 0.5) compared to 1 (IQR 1) at 1 year (p=0.045) (Fig. 3).

**Conclusion** Echocardiography-guided pacemaker optimisation modestly reduces LVOTO gradients, with further reduction over time and trend towards improvement in exertional symptoms. Longer-term follow-up data will confirm if this trend is continued and explore predictors of response.

**Abbreviations** HCM, hypertrophic cardiomyopathy; LVOTO, left ventricular outflow tract obstruction; PPM, permanent pacemaker.

**Fig. 1(abstract ABS033):**
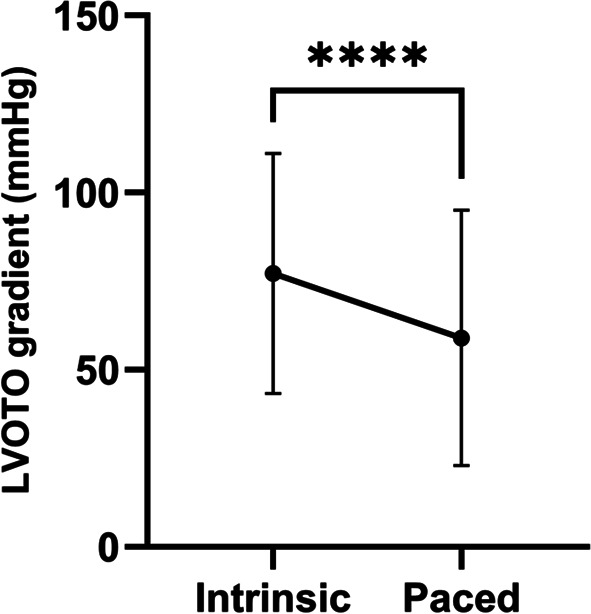
Acute change in LVOTO gradient between intrinsic sinus rhythm and paced ventricular activation. LVOTO, left ventricular outflow tract obstruction. ****=p<0.0001 in pairwise comparisons

**Fig. 2(abstract ABS033):**
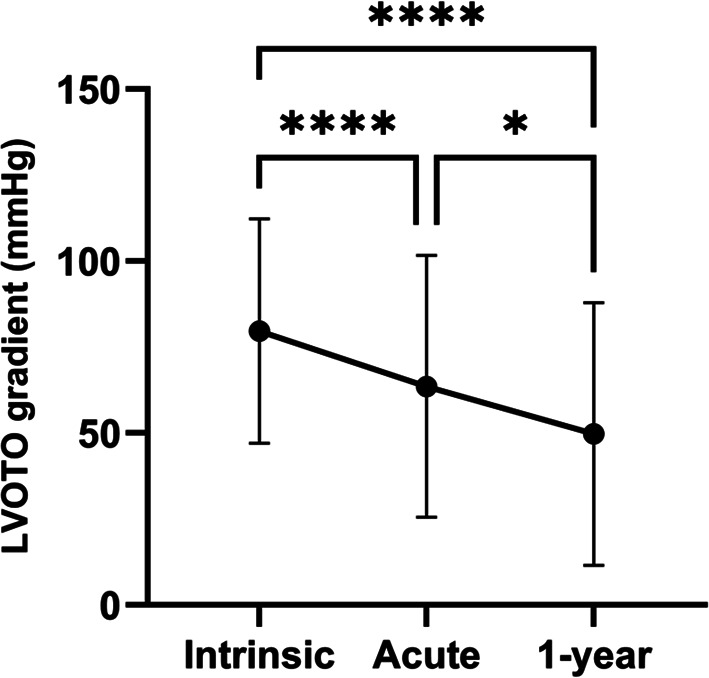
Change in LVOTO gradient between intrinsic and paced ventricular activation acutely, and further at 1 year in 53 patients with available follow-up data. LVOTO, left ventricular outflow tract obstruction. ****=p<0.0001, *=p<0.05 in pairwise comparisons

**Fig. 3(abstract ABS033):**
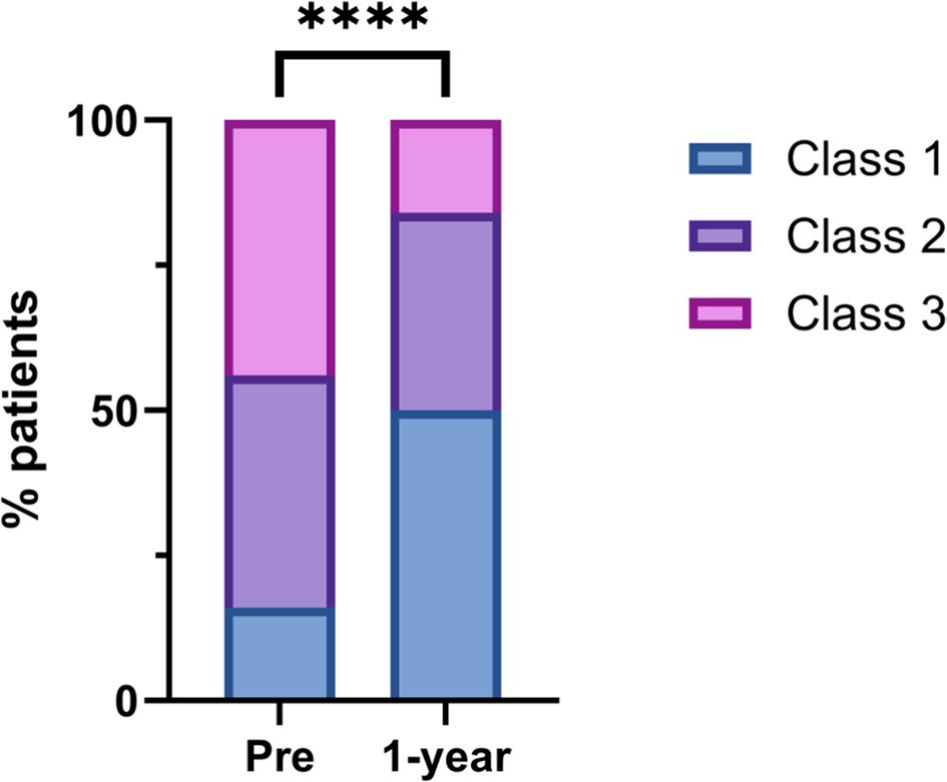
Change in NYHA class gradient between pre-optimisation and 1-year of follow-up in 53 patients with available data. ****=p<0.0001 in pairwise comparisons

## ABS034 A retrospective comparison of three international guidelines for the assessment of left ventricular diastolic function

Jacob Wehrle^1^

^1^Guy’s and St Thomas’ NHS Foundation Trust Hospital, London, UK

*Echo Research & Practice 2026*, **13(Suppl 1):**ABS034

In 2024, the British Society of Echocardiography released updated guidelines to assess left ventricular diastolic function, replacing pre-existing guidelines from the BSE and an alternative to the American Society of Echocardiography/European Association of Cardiovascular Imaging algorithm. Each guideline is unique and thus produces different gradings of diastolic function. Currently, the BSE-2024 guidelines have yet to be directly compared with alternative guidelines to identify whether similar diastolic gradings are provided. Ninety-four patients in sinus rhythm who underwent a transthoracic echocardiogram for the assessment of either left ventricular systolic function or heart failure were identified from the local echocardiography database. All three guidelines were used to assess left ventricular diastolic function and the gradings were compared. Chi-square tests of association and Cohen’s Kappa score were used to evaluate the data (significance: p-value <0.05). Overall, fewer patients were graded as indeterminate by the BSE-2024 guidelines (0%) compared to the ASE/EACVI guidelines (21.3%) and the BSE-2013 guidelines (40.4%). No significant differences were found between the BSE-2024 and ASE/EACVI guidelines for normal (60.6% vs 53.2%, P=0.499) or abnormal (39.4% vs 25.5%, P=0.096) gradings. Significantly more patients were graded as normal between the BSE-2024 and BSE-2013 (60.6% vs 28.7%, P<0.001) but no difference was found for abnormal (39.4% vs 30.9%, P=0.325) gradings. The addition of LA strain as a supplementary measurement along with fewer conflicting parameters throughout to ensure all grading routes are covered, both appear as key factors for fewer indeterminate gradings of diastolic function by the BSE-2024 algorithm for patients in sinus rhythm.

## ABS036 The comparison of transthoracic echocardiography and cardiac MRI in the estimation of left ventricular mass for the prediction of left ventricular hypertrophy

Caitlin Bugg^1^, Alexander McLaughlin^1^

^1^University Hospital Southampton, UK

*Echo Research & Practice 2026*, **13(Suppl 1):**ABS036

**Background** Left ventricular hypertrophy (LVH) is a predictor of cardiovascular mortality. Transthoracic echocardiography (TTE) and magnetic resonance imaging (MRI) can diagnose LVH by measuring LV mass. TTE is considered to overestimate LV mass, whilst MRI is seen as the gold-standard modality.

**Objective** To determine if TTE is as reliable as MRI when measuring LV mass and diagnosing LVH in a cohort of participants with no specific exclusion criteria.

**Methods** 422 participants were recruited from University Hospital Southampton who had received a TTE and an MRI less than 6 months between March 2023 - March 2024. A service evaluation was carried out to compare the data, with statistical analysis involving a Bland-Altman plot and a receiver operating characteristic (ROC) curve to assess the means and differences between the two modalities.

**Results** The median value for LV mass measured from TTE (62g/m^2^/53g/m^2^) was significantly higher than that of MRI (83g/m^2^/79g/m^2^) for males and females, respectively. TTE also reported more severe cases of LVH (11.6%/4.8%) when compared to MRI (4%/3.4%). Statistical analysis highlighted a mean difference between the two of -29.76g/m^2^. However, ROC analysis showed TTE to still have good diagnostic performance with an AUC (area-under-curve) of 0.83 compared to 0.98 with MRI.

**Conclusion** This study has highlighted that there is significant variation in LV mass when comparing imaging modalities, with TTE overestimating LV mass. Updated guidance regarding the formulae used to measure LV mass using TTE is required to improve LVH diagnosis in clinical use.

## ABS037 The predictive value of echocardiographic probability in pulmonary hypertension screening: a validation study under the revised haemodynamic definition

Hannah Morris^1^, Martin Johnson^1,2^

^1^Golden Jubilee University National Hospital, Clydebank, Scotland, ^2^Scottish Pulmonary Vascular Unit, Clydebank, Scotland

*Echo Research & Practice 2026*, **13(Suppl 1):**ABS037

**Background** Transthoracic echocardiography is the first-line screening method for pulmonary hypertension (PH). The British Society of Echocardiography (BSE) guidelines recommend assigning echocardiographic probability of PH using an algorithm centred on tricuspid regurgitant velocity (TRV) >2.8m/s. Additional indirect echo features further increase probability. Invasive right heart catheterisation (RHC) is indicated for increased probability patients.

**Purpose** TRV 2.8m/s equates to mean pulmonary artery pressure (mPAP) ~21-25mmHg. PH was redefined from mPAP ≥25mmHg to >20mmHg since adoption of the algorithm. This study aimed to validate the predictive value of the BSE algorithm using the lower >20mmHg definition.

**Methods** This retrospective, single-centre study included 300 patients that received both comprehensive echocardiography and RHC during 4-day admission from 2022-2024. Predictive value was assessed by receiver operator characteristic (ROC) curve analysis, and sensitivity/specificity analysis, comparing mPAP>20mmHg and mPAP>25mmHg definitions of PH.

**Results** Of 300 patients, 271 (90.3%) had PH (mPAP >20mmHg). ROC curve analysis of TRV in PH prediction confirmed good sensitivity and specificity (area under ROC 95%). Despite the lower haemodynamic definition of PH, reducing TRV cut-off only marginally improved sensitivity, at significant expense to specificity (<50%). TRV>3.1 m/s was identified as optimal over the current >2.8m/s cut-off (sensitivity 91.2%, specificity 93.7%). Inclusion of other echocardiographic PH features increased specificity and reduced false positives, particularly in low-probability patients.

**Conclusions** Our data suggests a higher TRV cut-off more accurately predicts PH at mPAP>20 mmHg and mPAP>25mmHg. Additional echo signs of PH provide little diagnostic value with TRV>3.4m/s; however, they are useful in low/intermediate probability patients. This study reports that the current BSE echocardiography algorithm for PH probability maintains comparable, robust predictive value under the new PH definition.

**Fig. 1(abstract ABS037):**
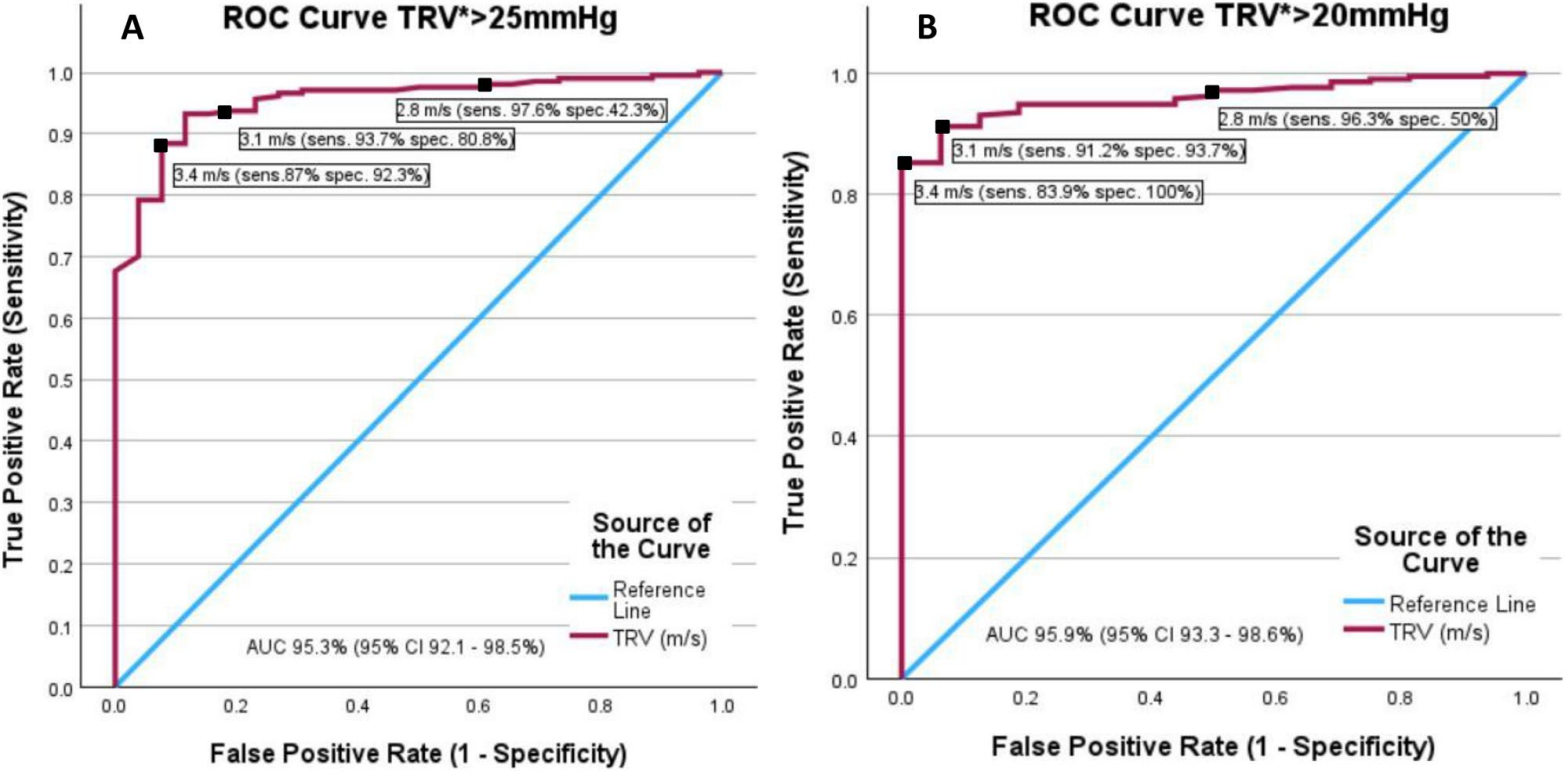
Receiver operating characteristic (ROC) curve of TRV for the prediction of PH defined at ≥25 mmHg (A) and >20 mmHg. (B). The ROC curve (red) plots sensitivity and specificity for predicting PH across a range of TRV values. Sensitivity and specificity values for TRV 2.8, 3.1 and 3.4 m/s are highlighted. Reference line (blue) represents 0.5. AUC, area under the curve; PH, pulmonary hypertension; ROC, receiver operating characteristic; sens., sensitivity; spec., specificity; TRV, tricuspid regurgitant velocity

**Table 13:**
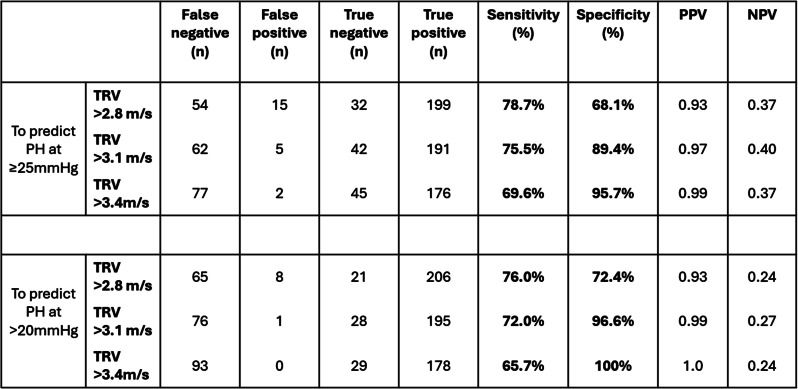
Sensitivity and specificity analysis of TRV cut-off values for prediction of PH defined at ≥25 mmHg and >20 mmHg. False negative, false positive, true negative and true positives are expressed as counts, sensitivity and specificity are expressed as percentages

**Table 14:**
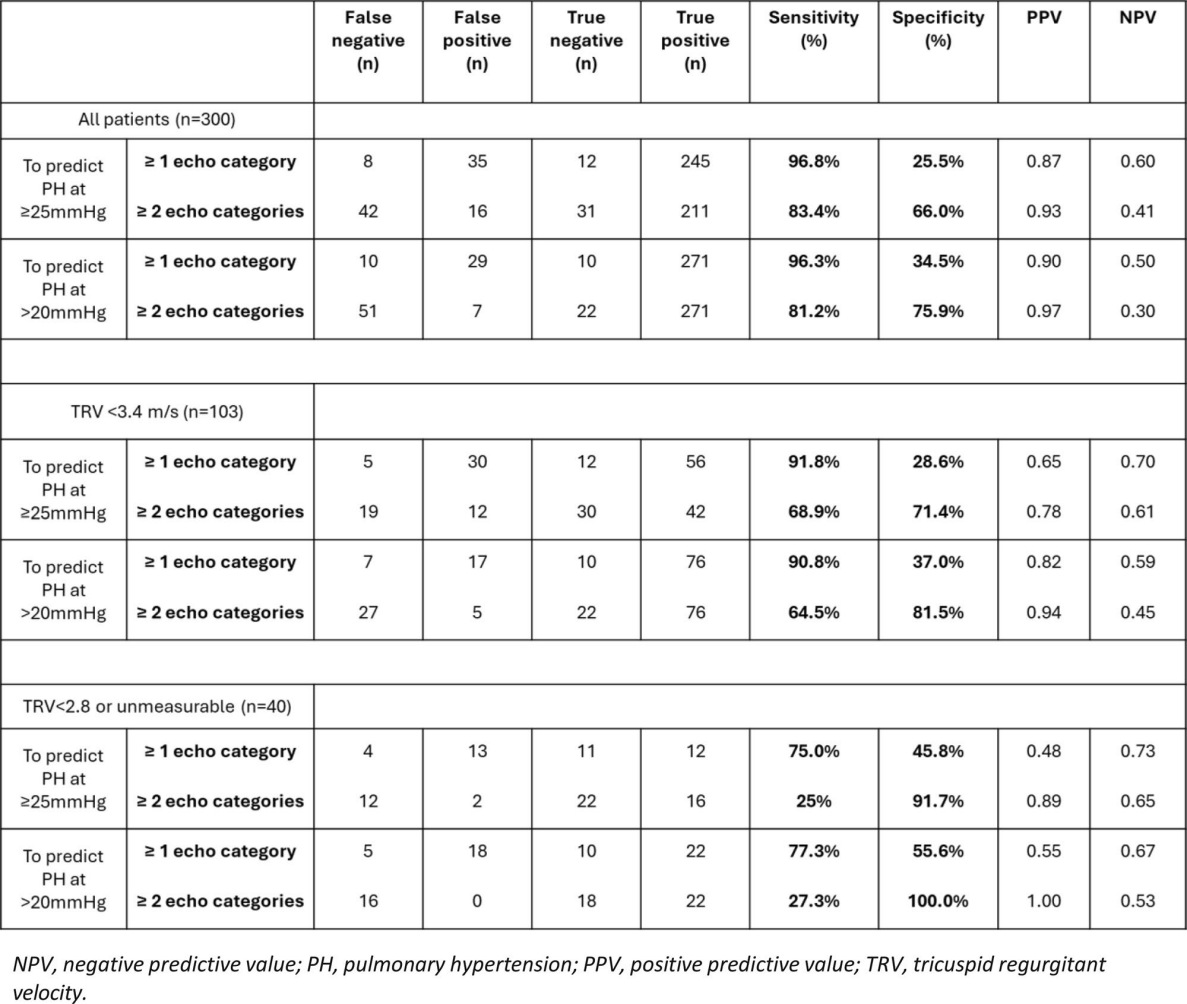
Sensitivity and specificity analyses for PH diagnosis using the presence of indirect echocardiographic signs of PH (as identified in the BSE algorithm). Analyses were performed in all patients, and in subgroups of patients assigned a low probability, and a low-or-intermediate probability

## ABS038 Evaluation of NT-proBNP level and LV function in the heart failure diagnostic pathway

Khin Lan^1^, Steven Hodgson^1^, Helen Oxenham^1^

^1^North Tees and Hartlepool NHS Foundation Trust, Stockton-on-Tees, UK

*Echo Research & Practice 2026*, **13(Suppl 1):**ABS038

**Introduction** Re-referral to the heart failure (HF) pathway is common. We wanted to evaluate whether patients referred for a repeat echocardiogram within a 4-year period following a second NT-proBNP result developed a change in LV systolic function.

**Methods** A retrospective audit identified 69 patients who had repeat assessment in the HF NT-proBNP pathway between 2018–2022 in one NHS Trust in England. LV ejection fraction (LVEF) and NT-proBNP levels were recorded and compared for each patient with each echocardiogram.

**Results** 11/69 (15.9%) of patients had a **new** change in LV function compared to their previous **normal** echocardiogram. 2 patients had LVEF ≤35%, 3 patients had LVEF 36-45%, 1 patient had LVEF 46-49% and 5 patients had LVEF 50-54%. 7 out of 11 patients (63.5%) who had a change in LV function had new AF. 3 out of 11 patients (27.2%) who developed LVEF ≤45% all were confirmed to have AF on their second echocardiogram and had an NT-proBNP level of > 600pg/ml. Patients who developed LVEF ≤35% had an NT-proBNP level of >2000pg/ml. The mean value for the change in NT-proBNP level is 269pg/ml for those without LV impairment. For those with LV impairment, the mean change in NT-proBNP level is 1222pg/ml whereas importantly for LVEF ≤35%, the value is 4594 pg/ml.

**Fig. 1(abstract ABS038):**
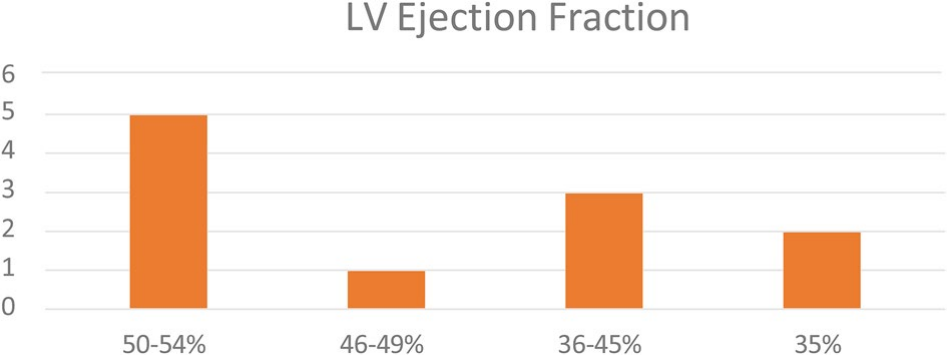
Change in LV function

**Fig. 2(abstract ABS038):**
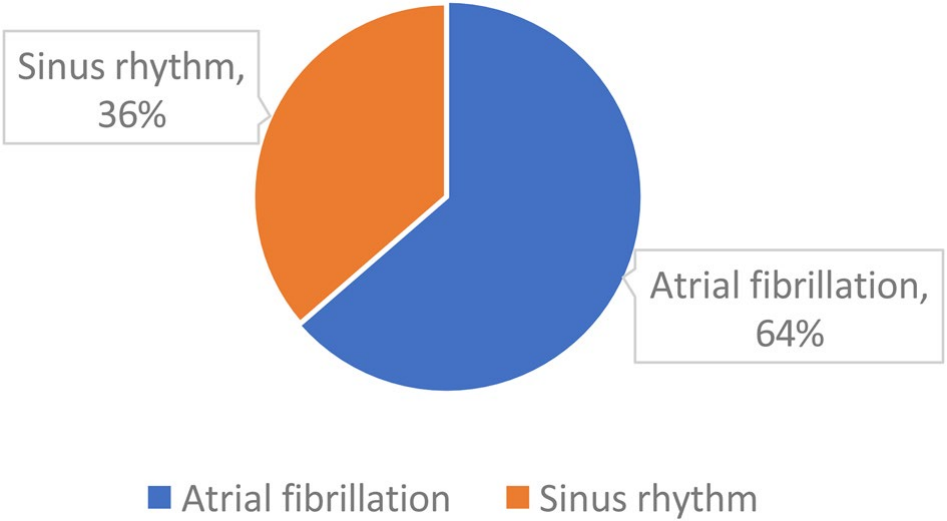
Presenting rhythm for repeat echocardiograms

**Conclusions** For patients to develop a new significant change in their LV function to LVEF ≤ 45%, they need a large numerical change in NT-proBNP level (>600pg/ml) or to have developed AF. Screening for AF should be considered as part of the re-referral pathway.

## ABS039 Comparison of manual, 2D automated, and 3D echocardiographic methods for measuring first-phase ejection fraction (EF1): A study of agreement and reproducibility

Labeed Ahmad^1^, Haotian Gu^2^, Francis Darrel^1^, Matthew Shun-Shin^1^

^1^Imperial College Healthcare NHS Trust, London, UK

^2^King’s College London, UK

*Echo Research & Practice 2026*, **13(Suppl 1):**ABS039

**Background** Left ventricular ejection fraction (EF) is a cornerstone of cardiac functional assessment but may overlook early systolic dysfunction, particularly in patients with preserved EF. First-phase ejection fraction (EF1), defined as the percentage change in volume from aortic valve opening to peak aortic flow, has emerged as a more sensitive and prognostically valuable metric. EF1 has demonstrated predictive utility in heart failure, aortic stenosis and hypertensive heart disease. However, no consensus exists on the optimal technique for EF1 measurement, and comparative reproducibility across manual, semi-automated, and 3D modalities remains unvalidated.

**Purpose** To compare the agreement and inter-operator reproducibility of manual 2D biplane, 2D semi-automated, and 3D echocardiographic techniques for EF1 measurement in clinical practice.

**Methods** Forty-seven patients were recruited. Method agreement was assessed in 32 patients using Bland–Altman analysis, repeated-measures ANOVA with post-hoc testing, and linear mixed-effects modelling. In 15 patients, two independent operators measured EF1 using all three techniques to assess inter-operator variability, quantified by within-subject standard deviation (SDw) and intraclass correlation coefficient (ICC).

**Results** All three techniques demonstrated excellent inter-operator reproducibility (ICC > 0.93), with 3D EF1 showing the highest consistency (SDw = 1.17, ICC = 0.967), compared to manual (SDw = 1.64, ICC = 0.937) and 2D automated (SDw = 1.87, ICC = 0.933). Mean EF1 values did not significantly differ between methods (ANOVA p = 0.70). Bland–Altman analysis revealed minimal bias and clinically acceptable limits of agreement across all pairwise comparisons.

**Conclusion** Manual, 2D automated, and 3D echocardiographic techniques for EF1 measurement demonstrate high inter-operator reliability and comparable results. These findings support EF1’s clinical utility suggests that multiple measurement approaches may be used interchangeably under optimal conditions. 
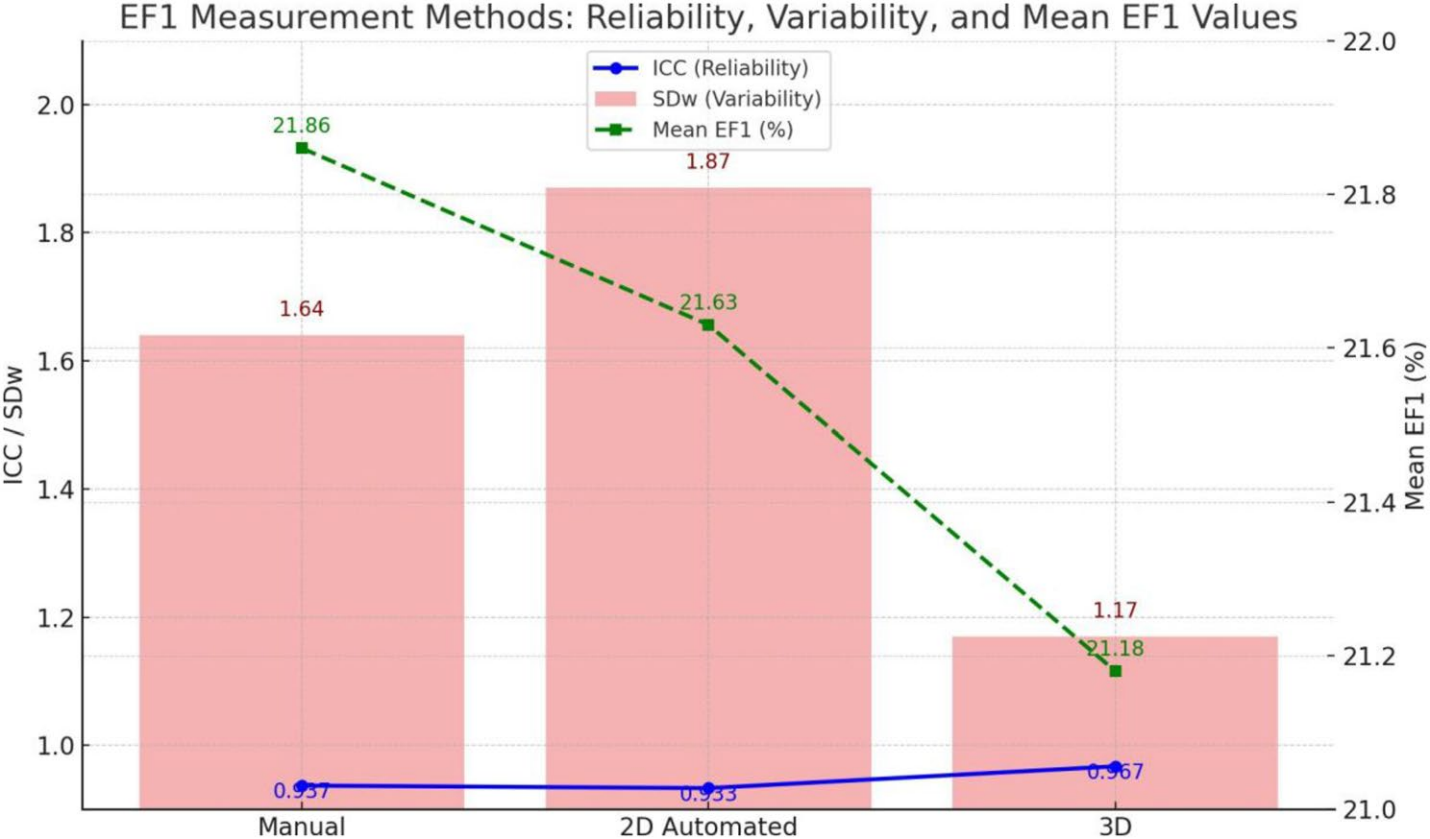


## ABS040 Improving appropriateness and efficiency of bubble echocardiography referrals for cryptogenic stroke: A quality improvement project

A. Rajendiran Tamilselvi^1^, S. Shepperd^1^, J. Redfern^1^

^1^Countess of Chester Hospital, UK

*Echo Research & Practice 2026*, **13(Suppl 1):**ABS040

**Background** Bubble echocardiography is a key investigation in the evaluation of cryptogenic stroke and should be reserved for patients who may be suitable for patent foramen ovale (PFO) closure. In May 2024, waiting times for bubble echocardiography at our medium-sized District General Hospital exceeded 16 weeks. We conducted a quality improvement project to enhance referral appropriateness and reduce waiting times, thereby promoting more efficient use of NHS resources.

**Methods** A retrospective review of 48 bubble echocardiogram referrals from 2023 was undertaken using NHS England criteria for PFO closure (Fig. 1). An education session was delivered to the stroke department, the electronic referral form was updated to align with national guidelines, and all referrals were formally triaged. A further 50 referrals from 2024 were reviewed to assess the impact.

**Fig. 1(abstract ABS040):**
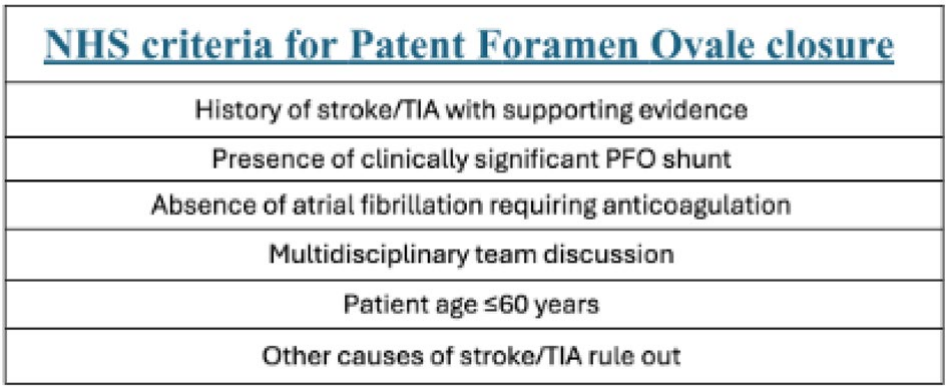
NHS England criteria for PFO closure

**Results** Of the 48 patients reviewed in 2023, 44% (n=21) had a shunt. Among these, 43% (n=9) were referred, and 56% (n=5) underwent PFO closure. Following triage, 24% (n=12) of 2024 referrals were rejected. Of the remaining patients, 21% (n=8) had a PFO, and 38% (n=3) were referred for closure. All three were accepted. Appropriate referral rates improved from 69% to 92%, acceptance for PFO closure increased from 54% to 100% (Fig. 2), and waiting times decreased from 16 to 10 weeks.

**Fig. 2(abstract ABS040):**
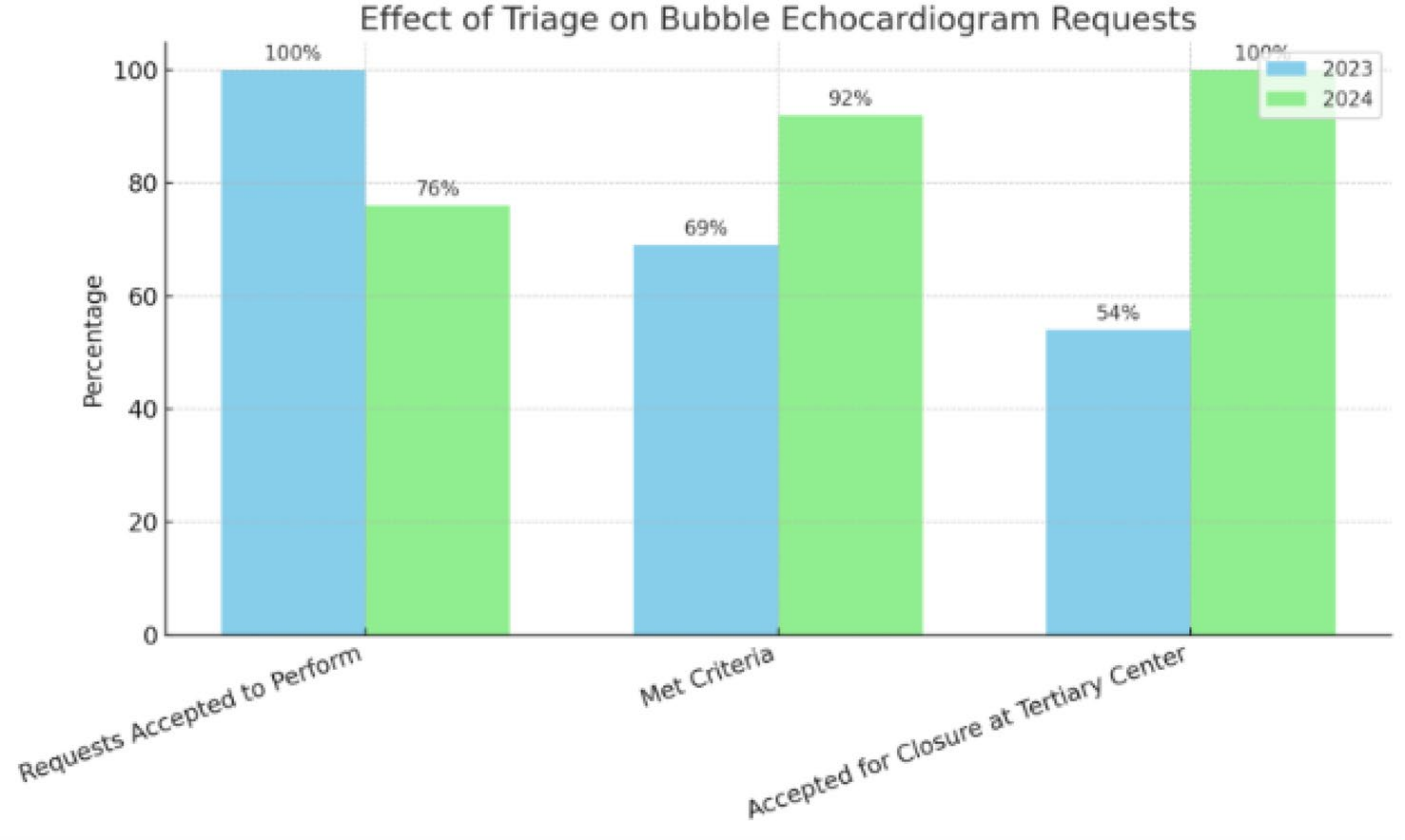
Effect of triage on requesting of Bubble echocardiograms and subsequent acceptance for closure

**Conclusion** Formal triage of bubble echocardiogram referrals in line with NHS England guidelines significantly reduced inappropriate investigations and waiting times, and improved acceptance rates for PFO closure.

## ABS042 Early systolic tricuspid annular motion as a novel marker of right ventricular function in patients with hypertrophic cardiomyopathy

Jie Shen^1^, Ernestene Vasa^1,2^, Gerry Carr-White^2^, Phil Chowienczyk^1^, Haotian Gu^1,2^

^1^School of Cardiovascular & Metabolism Medicine and Science, King’s College London, UK, ^2^Guy’s and St Thomas’ Hospital, NHS Foundation Trust, London, UK

*Echo Research & Practice 2026*, **13(Suppl 1):**ABS042

**Introduction** Hypertrophic cardiomyopathy (HCM), including its obstructive form (HOCM), primarily affects the left ventricle, but accumulating evidence demonstrates subtle yet clinically meaningful right ventricular (RV) involvement. First-phase TAPSE (TAPSE1), a simple measure of early RV longitudinal function, may be more sensitive in detecting early RV impairment.

**Purpose** To evaluate whether TAPSE1 detects RV dysfunction in patients with HCM in a single centre prospective study.

**Methods** Sixty adults (HOCM=13, non-obstructive HCM=30, and control=17) underwent echocardiography in the Inherited Cardiac Conditions Clinic at St Thomas’ Hospital who were prospectively recruited. TAPSE1 was defined as the longitudinal displacement of the tricuspid annulus from the onset of systole to the time of peak pulmonary flow velocity as shown in Fig. 1.

**Results** Demographics and echocardiographic parameters are summarised in Table 15. Age and body surface area were significantly higher in the HOCM and HCM groups compared to controls. Left ventricular ejection fraction (LVEF) was preserved across all groups (p=0.692). TAPSE or RV fractional area change did not differ significantly between groups (Fig. 2). However, TAPSE1 was significantly reduced in HCM compared to controls (0.70 ± 0.25 vs. 1.07 ± 0.31 cm, p<0.001), while no significant difference was observed between HCM and HOCM (Fig. 2). TAPSE1 correlated with conventional TAPSE (r=0.558, p<0.001), RV E velocity (r=0.420, p=0.011), and pulmonary valve maximum resting gradient (r=0.285, p=0.041), and was inversely associated with age (r=-0.480, p<0.001), body surface area (r=-0.362, p=0.008) and left atrial diameter (r=-0.372, p<0.007), but showed no association with LVEF (r=0.046, p=0.745).

**Conclusion** TAPSE1 is reduced in patients with non-obstructive HCM and has the potential to detect subtle RV dysfunction. Further studies are required to confirm its clinical utility.

**Table 15:**
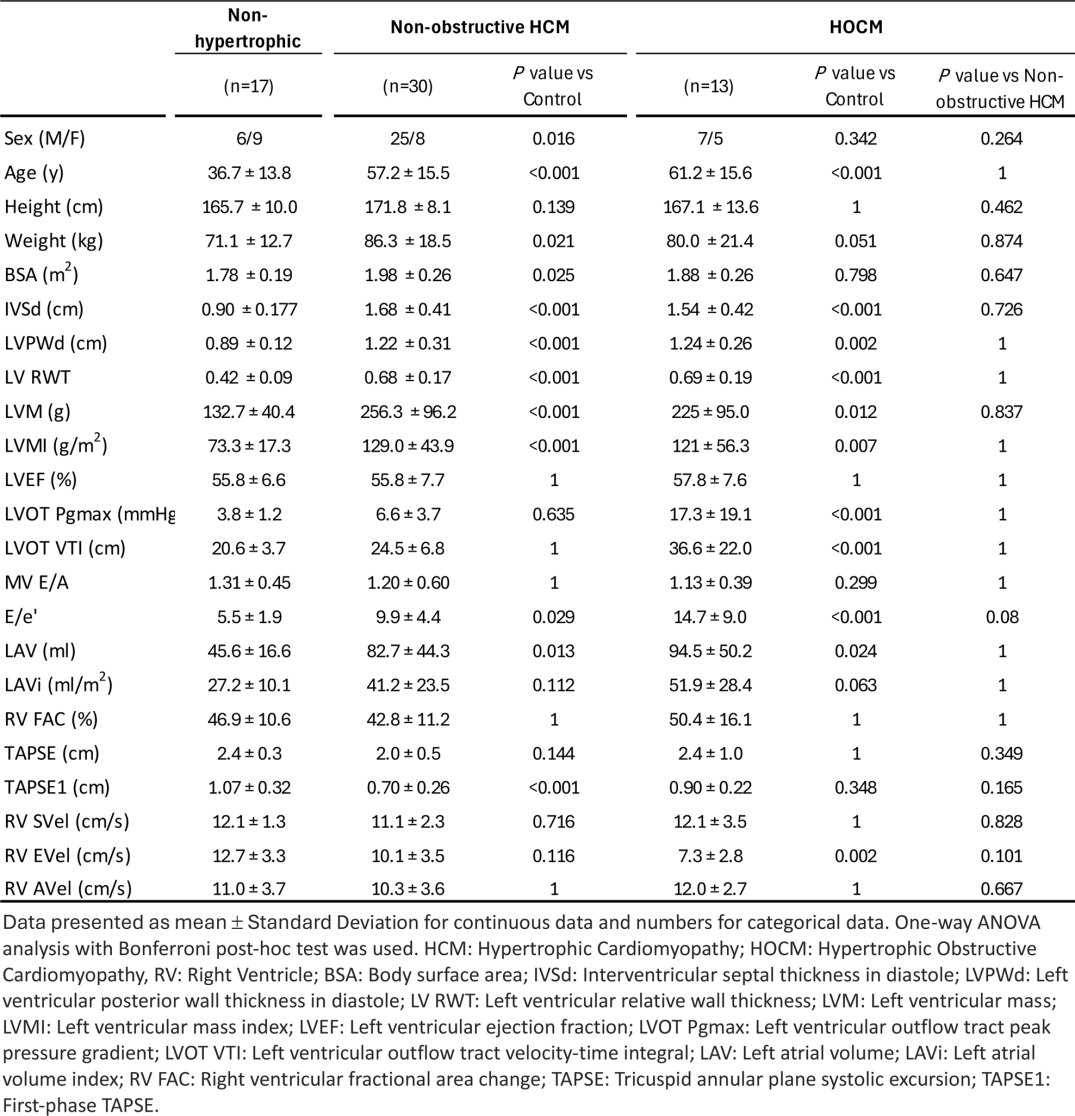
Demographics and echocardiogram measurements of groups

**Fig. 1(abstract ABS042):**
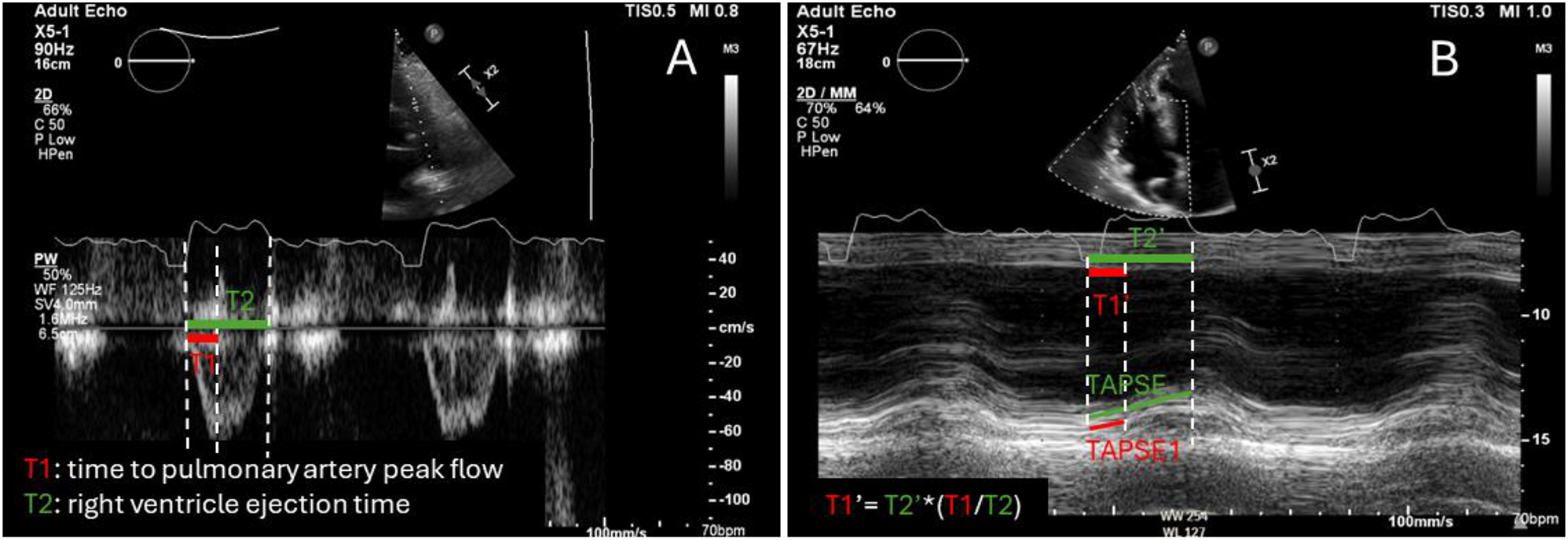
Measurement of first-phase TAPSE (TAPSE1). TAPSE1 was determined on echocardiogram by initially calculating the ratio (T1/T2) between the interval from the onset of systole to peak pulmonary artery flow velocity (T1) and total right ventricular (RV) ejection time (T2), obtained via pulsed-wave Doppler. This ratio was then applied to RV ejection time (T2’) measured separately in the apical four-chamber view to derive the corresponding interval (T1’). TAPSE1 thus represents the tricuspid annular displacement measured from the beginning of systole to the calculated time point (T1’)

**Fig. 2(abstract ABS042):**
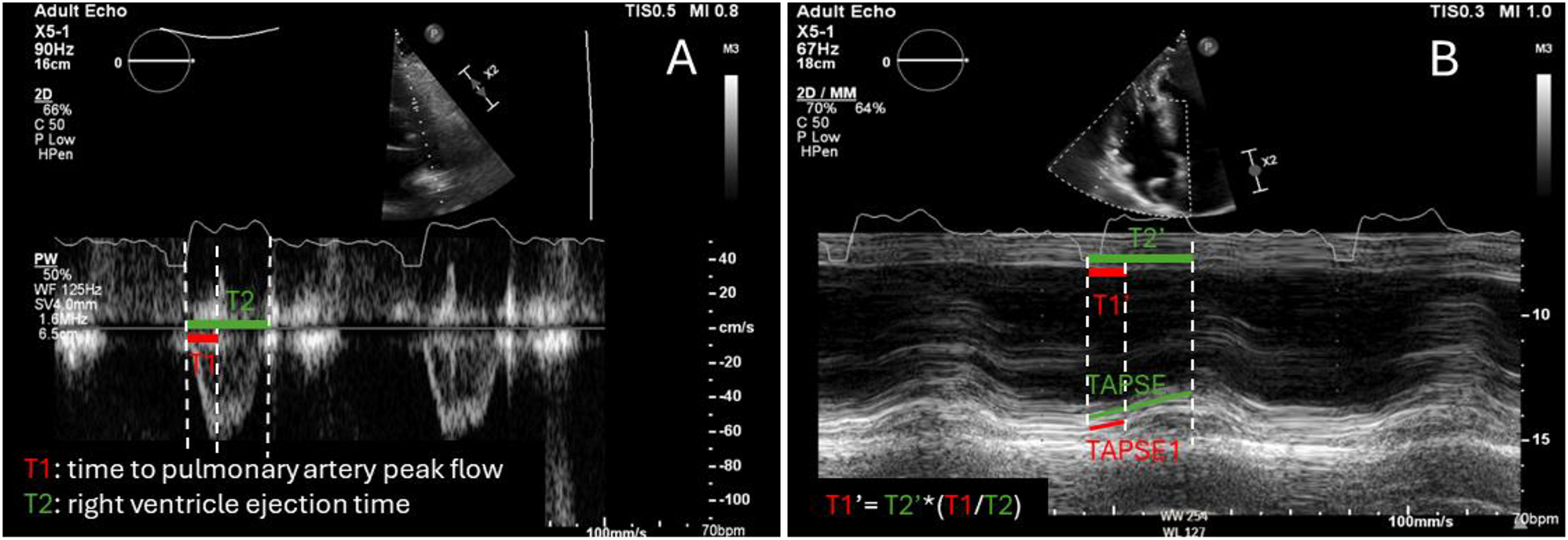
Right ventricular function assessed by tricuspid annular plane systolic excursion (TAPSE), right ventricular fractional area change (RVFAC), and first-phase TAPSE (TAPSE1) in patients with hypertrophic cardiomyopathy with and without left ventricular outflow tract obstruction, and in control subjects. Data shown at mean ± Standard Deviation. One-way ANOVA analysis with Bonferroni post-hoc analysis to control for multiple comparison. Ns defined as a p value ≥ 0.05

## ABS043 The incidence of coronary events within 12 months of a negative stress echocardiogram: a service evaluation of stress echocardiography at Queen Alexandra Hospital

Charley Sanford^1,2^, Emma Lane^1^, Munkit Choy^2^

^1^Portsmouth Hospitals University NHS Trust, UK, ^2^Manchester Metropolitan University, UK

*Echo Research & Practice 2026*, **13(Suppl 1):**ABS043

**Background** Stress echocardiography (SE) is a widely used imaging modality for the diagnosis and functional assessment of coronary artery disease (CAD). It is recommended for use in patients with 15-85% clinical likelihood of CAD (CL-CAD). High specificity is reported but is dependent on image quality, operator experience and patient demographics and risk factors.

**Aims**

- Assess the negative predictive value of SE
- Explore CL-CAD for patients referred for SE
- Explore the utilisation of optimisation techniques for SE

**Methods** A single-centre, observational cohort study was performed including all negative SEs completed between January 2022 - October 2023 (N=132). 12 patients were excluded due to missing follow-up data. CL-CAD was calculated for each patient. Retrospective data was collected from Portsmouth Hospitals University NHS Trust databases. Analysis was performed using descriptive statistics.

**Results** Of 120 patients (61.1 ± 14.2 years, 53.3% male), 1 patient experienced a coronary event. The negative predictive value was 0.99. Hypertension was the most common risk factor (52.2%) and raised BMI the least (15.6%). The mean (± SD) CL-CAD was 12% (± 0.11%), with 71.8% of those referred being classified as ≤ low CL-CAD. 80% of SEs used SonoVue contrast agent, 95% achieved target heart rate and 100% were reported by an experienced operator.

**Conclusion** SE at this centre provides highly specific results indicating effective delivery of service. The specificity and utilisation of optimisation techniques are comparable to large scale, multi-centre research, however, results were obtained from a population with a low CL-CAD.

## ABS044 Reduced global longitudinal active strain energy density in hypertrophic cardiomyopathy

Magda-Madalina Olaru^1^, Khalda Halim^1,2^, David H. MacIver^3,4^, Paulo-Angelo Bulleros^1,2^, Sanjay Sharma^1,2^

^1^St George’s Hospital, London, UK, ^2^Cardiology Academic Group, St George’s University of London, UK, ^3^Biological Physics Group, University of Manchester, UK, ^4^Musgrove Park Hospital, Taunton, UK

*Echo Research & Practice 2026*, **13(Suppl 1):**ABS044

**Introduction** Global longitudinal active strain energy density (GLASED) is a novel metric that quantifies myocardial work per unit volume by combining global longitudinal strain and wall stress, and is a measure of myocardial (dys)function. Recent studies demonstrate GLASED’s superior prognostic value compared to ejection fraction and strain.

**Purpose** We evaluated GLASED in athletes and non-athletes with hypertrophic cardiomyopathy (HCM).

**Methods** Thirty-seven asymptomatic individuals (19 athletes, 18 non-athletes) were assessed; 35 with clinically diagnosed HCM, one normal athlete (Case 1) and one with an uncertain phenotype (Case 24). GLASED was calculated from the global longitudinal strain (GLS), left ventricular end-diastolic wall thickness, internal diameter and systolic blood pressure.

**Results** The table summarises 4 of the cases in more detail. Mean peak GLS was –16.5% in HCM athletes versus -13.7% in HCM non-athletes (*P*<0.01). Figure 1 shows the GLASED results for all cases. Mean GLASED was significantly higher in athletes versus non-athletes (Fig. 2). Marked regional variation was observed in Case 2 with segmental longitudinal active strain energy density (Fig. 3). Case 24 had an uncertain phenotype but demonstrated a low GLASED of 1.61 kJ/m^3^. Gene-positive had a significantly lower GLASED than gene-negative HCM patients (Fig. 4).

**Conclusions** GLASED is significantly reduced in HCM with substantial segmental variation correlating with local wall thickness. Athletes with HCM had higher GLASED values than non-athletes with HCM and gene+ HCM had lower GLASED than gene- HCM individuals. GLASED shows promise as an enhanced diagnostic tool for detecting and characterizing HCM severity.

**Table 16:** Example results from 4 cases with differing HCM severity

Parameter	Control	Mild HCM genotype negative	Mild HCM MYBPC3 positive	Severe HCM MYBPC3 positive
Case no.	1	4	3	2
Age (yrs)	27	38	20	23
Sex	Male	Male	Male	Male
SBP (mmHg)	130	116	100	120
LVIDD (mm)	57	50	46	45
EDWT (mm)	8.8	12.6	11.6	16.3
LVEF (%)	68	60	65	75
GLS (%)	−20.0	−20.0	-16.7	−15.1
GLASED (kJ/m^3^)	2.43	1.23	0.88	0.73

GLASED deteriorates in more severe HCM phenotypes

**Fig. 1(abstract ABS044):**
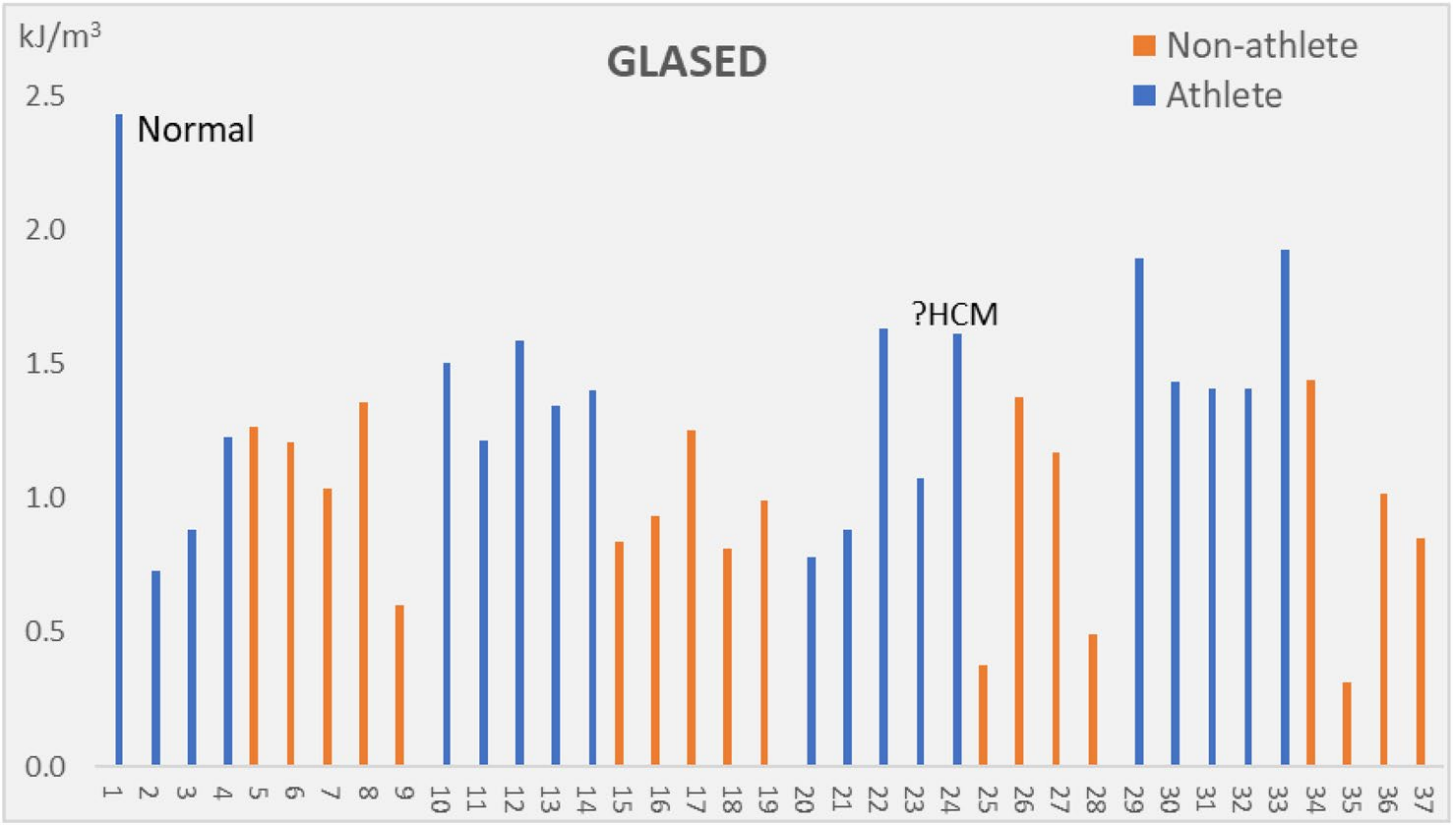
GLASED values in individual cases. GLASED is substantially reduced in all HCM patients

**Fig. 2(abstract ABS044):**
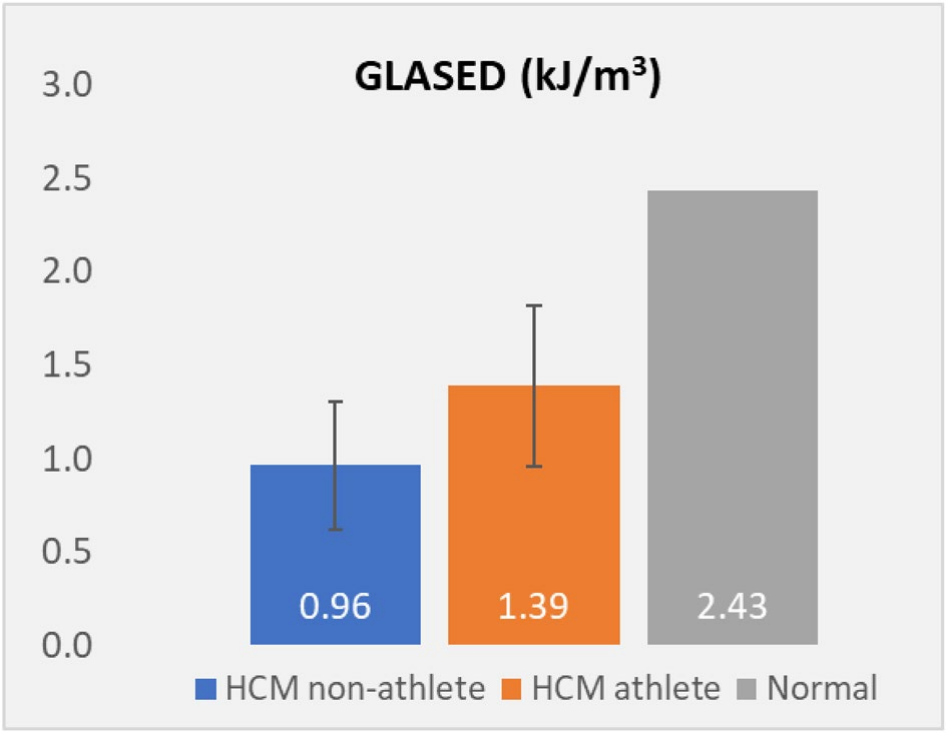
Mean GLASED (±1SD) in HCM athletes and HCM non-athletes compared to the normal

**Fig. 3(abstract ABS044):**
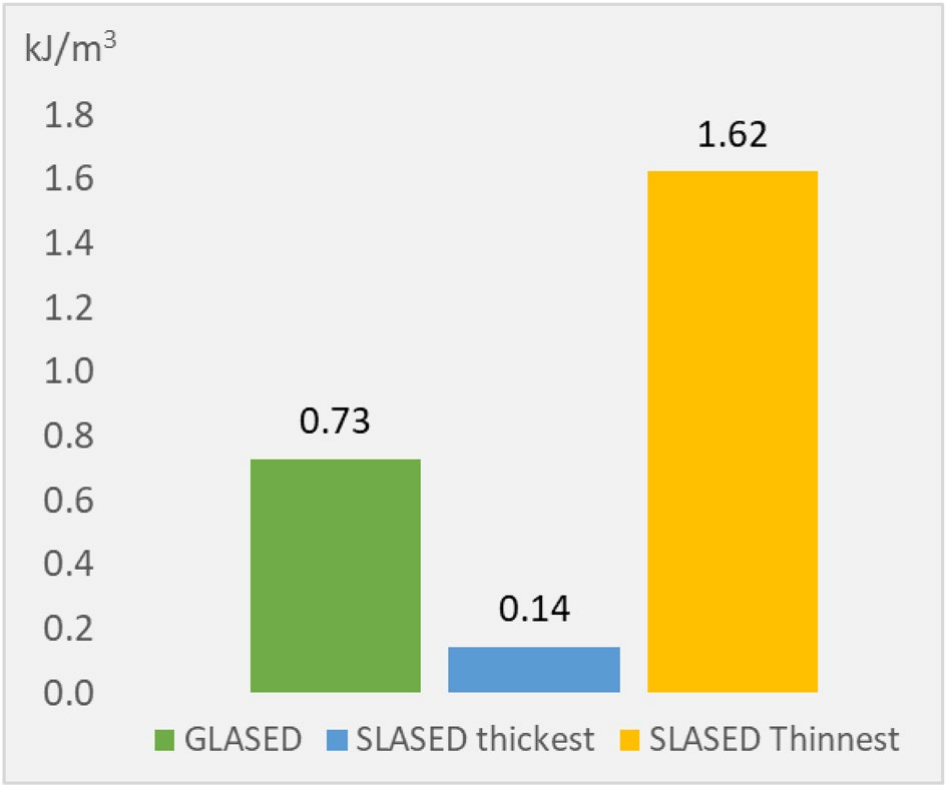
Comparison of GLASED and segmental longitudinal active strain energy density (SLASED). In the most abnormal segment (thickest) from Case 2, the SLASED is severely reduced. However, in the thinnest segment the SLASED is mildly reduced. The GLASED is moderately reduced compared to normal

**Fig. 4(abstract ABS044):**
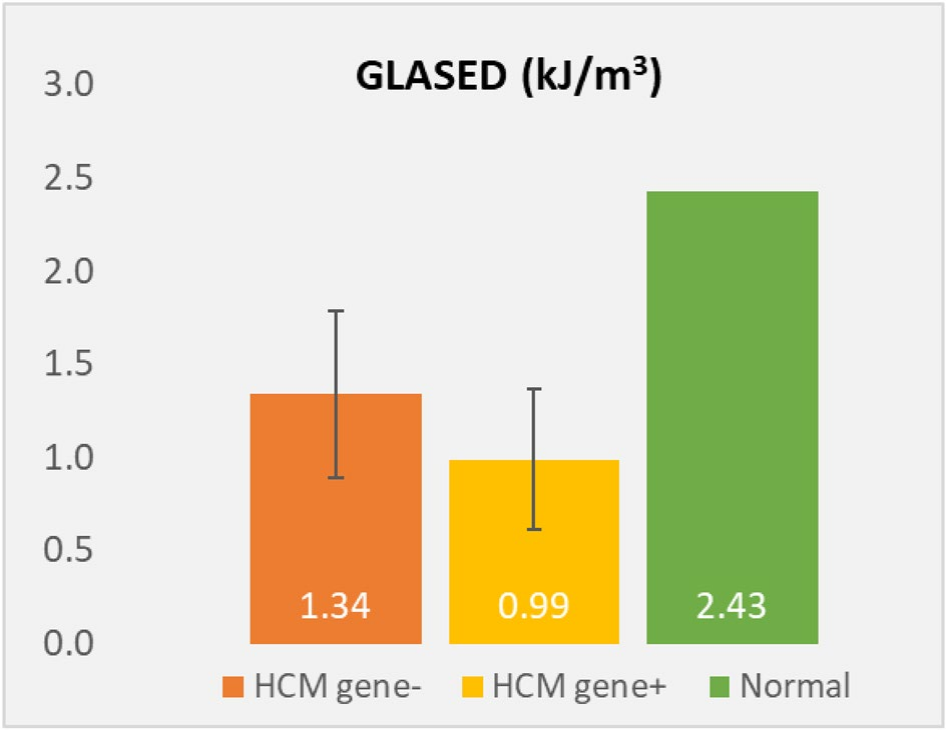
Comparison of GLASED in HCM gene- vs. HCM gene+ vs control athlete. Patients with gene- HCM have moderately reduced GLASED and gene+ HCM have severely reduced GLASED (mean±1SD, *P*<0.05)

## ABS045 BREATHE-QI. Breathlessness Evaluation with CPET-SE: Assessing Clinical Effectiveness – A Quality Improvement Project

Selda Ahmet^1,2^, Will Ricketts^1^, David Bruce^1^, Delfin Encarnacion^1^, Ashley Ashdown^1^, Sveeta Badiani^1^, Sanjeev Bhattacharyya^1^, Guy Lloyd^1,2^

^1^St Bartholomew’s Hospital, Barts Health NHS Trust, London, UK, ^2^Cleveland Clinic London, London, UK

*Echo Research & Practice 2026*, **13(Suppl 1):**ABS045

**Abbreviations** Breathing Pattern Disorders (BPD); Cardiopulmonary exercise test combined with stress echocardiogram (CPET-SE); Multidisciplinary team meeting (MDT); Referral-to-treatment time (RTT)

**Background** Chronic breathlessness is associated with high morbidity, mortality, and healthcare utilisation. Diagnostic delays are common, particularly in undifferentiated breathlessness, due to fragmented pathways. Cardiopulmonary exercise tests with stress echocardiography (CPET-SE) provide a comprehensive assessment of underlying mechanisms of breathlessness.

**Purpose** To evaluate the impact of an upfront CPET-SE–driven pathway on diagnostic accuracy and service efficiency in undifferentiated breathlessness.

**Methods** A quality improvement project using PDSA methodology was performed at Barts Health NHS Trust (Fig. 1). Previously, patients referred from North East London for undifferentiated breathlessness underwent respiratory-led assessment, with cardiac input via a separate pathway. The interventions introduced were (i)upfront CPET-SE for all referrals; and (ii)joint cardio-respiratory MDT to agree a diagnosis and streamline care (Fig. 2). Outcomes at 12 months included diagnostic distribution, referral-to-treatment time (RTT), cost savings and patient feedback.

**Results** Seventy-two patients (mean age 54.6, 63% female) were included. CPET-SE was abnormal in 93% (n=67) and a diagnosis was made; significant disease was excluded in the remainder. MDT outcomes were: (a) respiratory 35% (n=25); (b) cardiac 8% (n=6); (c) mixed cardio-pulmonary 10% (n=7); and (d) non-cardiopulmonary 40% (n=29) mainly comprising breathing pattern disorders (n=19) and obesity with deconditioning (n=10). Twenty-five percent were discharged or streamlined to other specialist clinics, while 68% continued on the pathway for targeted management, including Respiratory Physiotherapy. RTT shortened in 63% of patients, with estimated savings of £1,077/patient. All service users gave positive feedback.

**Conclusion** An upfront CPET-SE pathway improves diagnostic clarity in undifferentiated breathlessness, enables earlier targeted treatments, and has potential cost benefit.

**Fig. 1(abstract ABS045):**
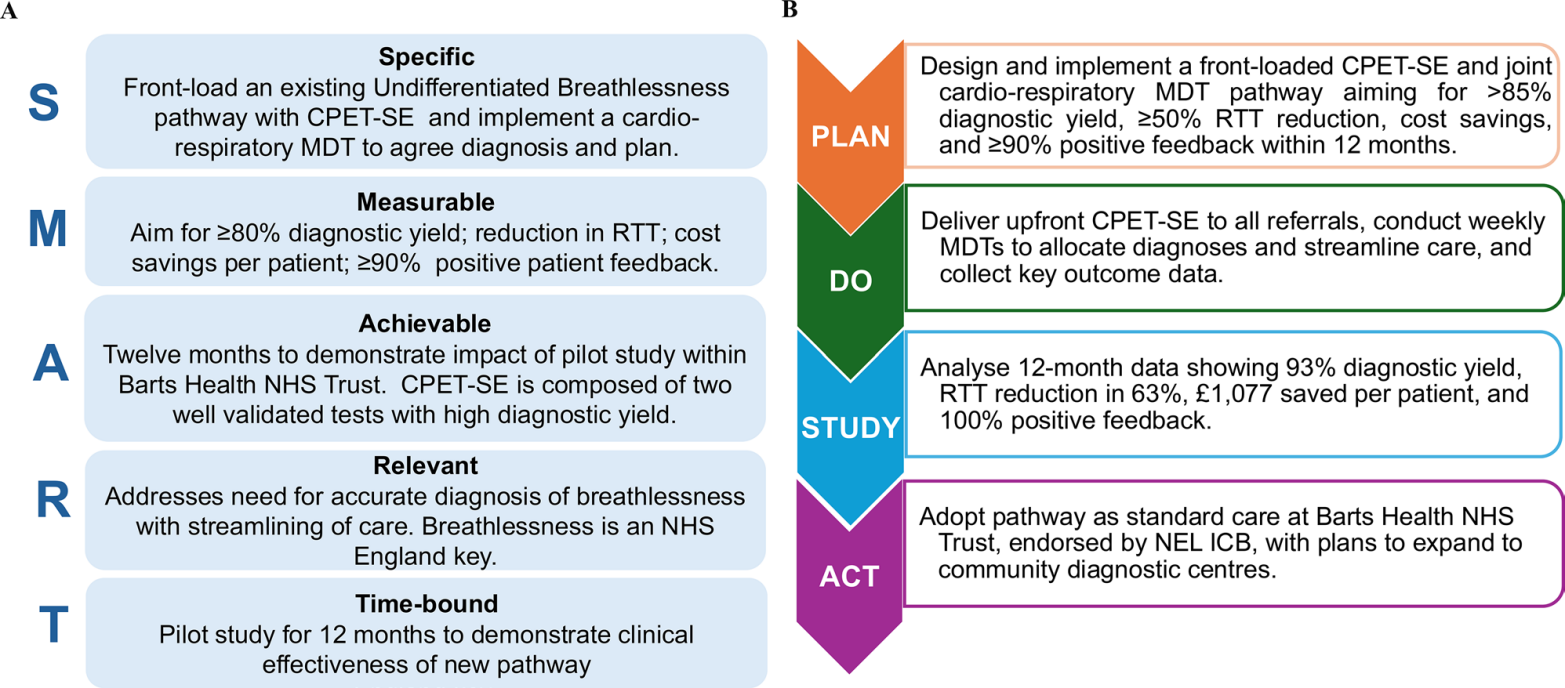
SMART Alms and PDSA Methodology. 1A. Summary of SMART Aims. The SMART framework was used to define specific objectives that were realistic, relevant, and measurable within a set period of time, CPET-SE = Cardiopulmonary Exercise Test combined with Stress Echocardiography; MDT= Multi-disciplinary Team Meeting; RRT = Referral to treatment time; SMART = Specific Measurable, Achievable, Relevant, Time-Bound. 1B. Summary of PDSA Methodology. The PDSA methodology provided a structured approach to testing, evaluating, and implementing the objectives identified by the SMART framework in Figure 1A. MDT=Multi-disdplinary team meeting; NEL/CB= North-East London Integrated Care Board; PDSA = Plan, Do, Study, Act

**Fig. 2(abstract ABS045):**
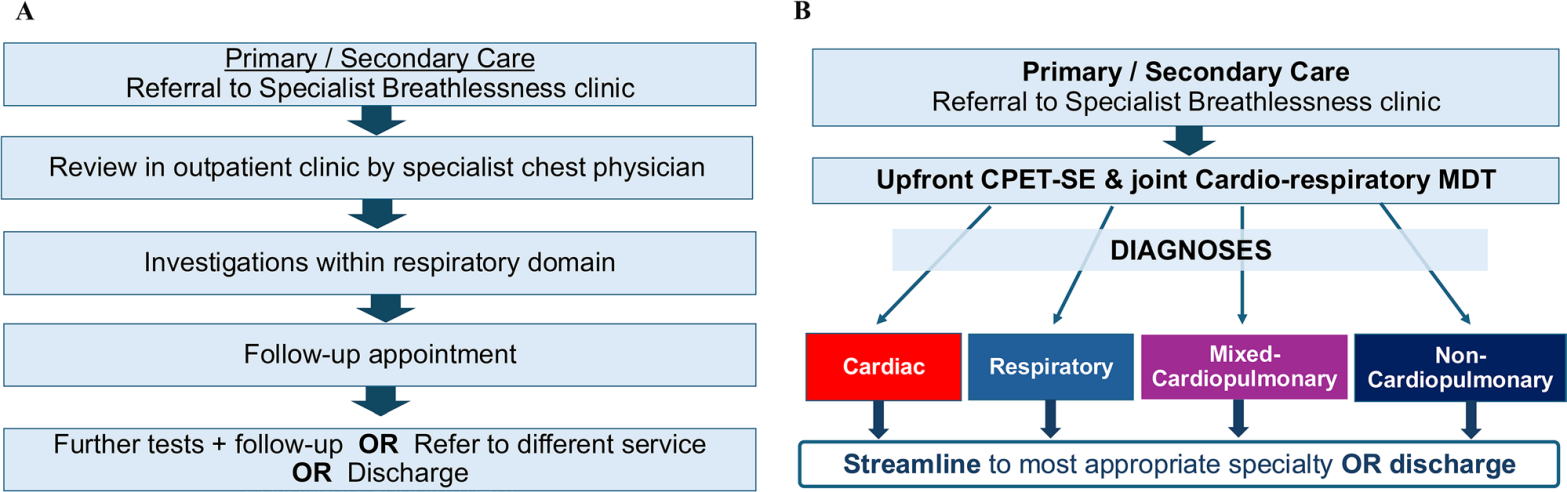
Undifferentiated Breathlessness Pathway Traditional vs Quality Improvement Project (QIP). 2A Undifferentiated Breathlessness pathway prior to QIP. The traditional pathway involved referral to the Specialist Breathlessness Clinic for assessment by a specialist respiratory physician and initial respiratory investigations, Test results were reviewed at a follow-up clinic. If the diagnosis remained unclear, additional tests with further follow-up or referral to another service (e.g., cardiology) were arranged. 2B New Undifferentiated Breathlessness pathway from QIP. The pathway was modified by introducing upfront CPET-SE, followed by a joint cardio-respiratory MDT to agree a diagnosis. Diagnoses were classified into four domains: cardiac, respiratory, mixed cardio-respiratory, and non-cardiopulmonary (e.g., breathing pattern disorders, obesity, deconditioning). Patients were streamlined to the appropriate services for treatment. Patients with normal CPET-SE were either discharged or retained on the pathway if they had complex comorbidities

## ABS046 Point-of-service questionnaire: a tool for implementing appropriate use criteria to manage echocardiography waiting lists

Kaivalya Divekar^1^, Harry Williams^1^, Hoda Abdelgawad^1^, Eleanor McPhail^1^, Nishat Jahagirdar^1^, Emily Denman^1^, Peter Pearson^1^, Alexandros Papachristidis^1^, George Amin Youssef^1^, Mehdi Eskandari^1^, Tom Marwick^2^

^1^. King’s College Hospital NHS Foundation Trust, London, UK, ^2^. Baker Heart and Diabetes Institute, Melbourne

*Echo Research & Practice 2026*, **13(Suppl 1):**ABS046

**Background** Widespread availability and affordability of transthoracic echocardiography (TTE) contributes to overuse, leading to long waiting lists and limited access for high-priority patients. The Point-of-Service-Questionnaire (PSQ) condenses appropriate use criteria into four key questions.

**Purpose** We assessed the PSQ’s utility in optimising TTE waiting list management by evaluating: i)validity in a universal healthcare setting, and ii)outcomes of patients with appropriate vs. inappropriate requests.

**Methods** To identify rarely-appropriate (RA) TTE requests, we applied the PSQ (RA=2 or more positive questions):

1. No new cardiovascular symptoms/signs/change in clinical status?
2. Routine-Surveillance(RS) scan outside guideline-recommended timeframes?
3. Prior TTE <12 months?
4. Suspected endocarditis without positive bacteraemia or new murmurs?

The PSQ was retrospectively applied to 100 echo requests to assess safety and then prospectively implemented in a Southeast-London tertiary care hospital, to screen 1,401 waiting list echo requests from 10/2023 to 5/2024. Incidence and proportions of adverse events (cardiovascular-related admissions) on the wait list in 1. Likely-Appropriate(LA)(scoring 0/1 on PSQ), 2. RA, and 3. RS (within guideline-recommended timeframes) exams were compared using Fisher’s exact test.

**Results** Among 1,401 patients (mean age 58.7±18.8years, mean wait time 49.7 ± 5.3weeks), 24 cardiovascular-related acute admissions occurred, all in 799 LA TTEs (3%). No readmissions among 113 RA (p=0.06) or among 212 RS TTEs noted(p=0.005) (Fig. 1). PSQ had 100% specificity for identifying low-risk cohorts. Compared to Fonseca et-al., who identified routine surveillance (<1 year) of known cardiomyopathy(19%) and asymptomatic screening of LV function(14%) as the most frequent RA indications, our cohort showed routine surveillance of heart failure patients without clinical change(22%) and pre-surgical echoes(16%) as the most common (Fig. 2).

**Conclusion** PSQ effectively identifies low-risk TTE requests with high specificity, offering a structured approach to reducing unnecessary scans while improving access for high-priority patients in a universal healthcare system.

**Fig. 1(abstract ABS046):**
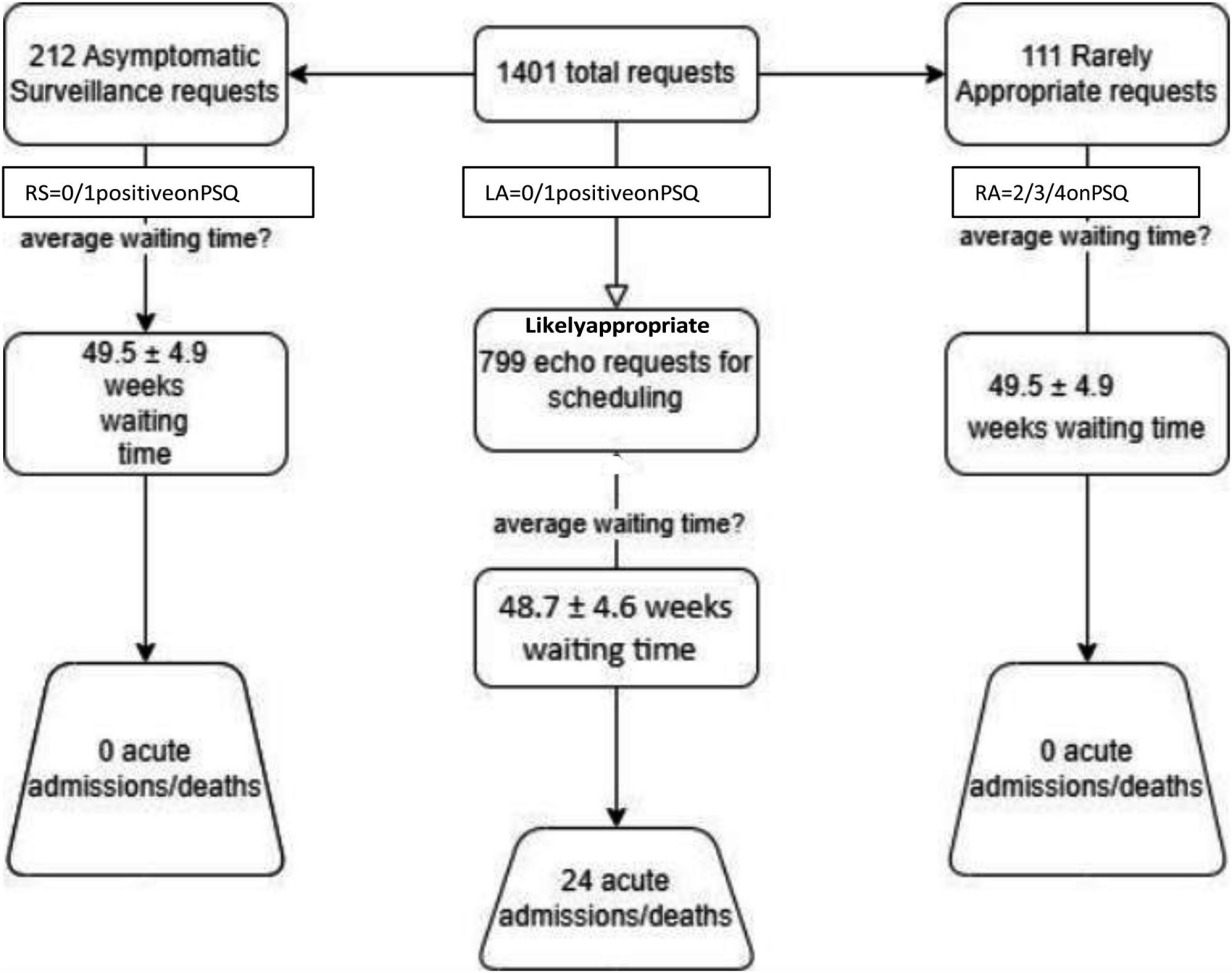
Patient outcomes. Flowchart depicting comparisons of outcomes over resultant waiting times of LA, RA, and RS echoes

**Fig. 2(abstract ABS046):**
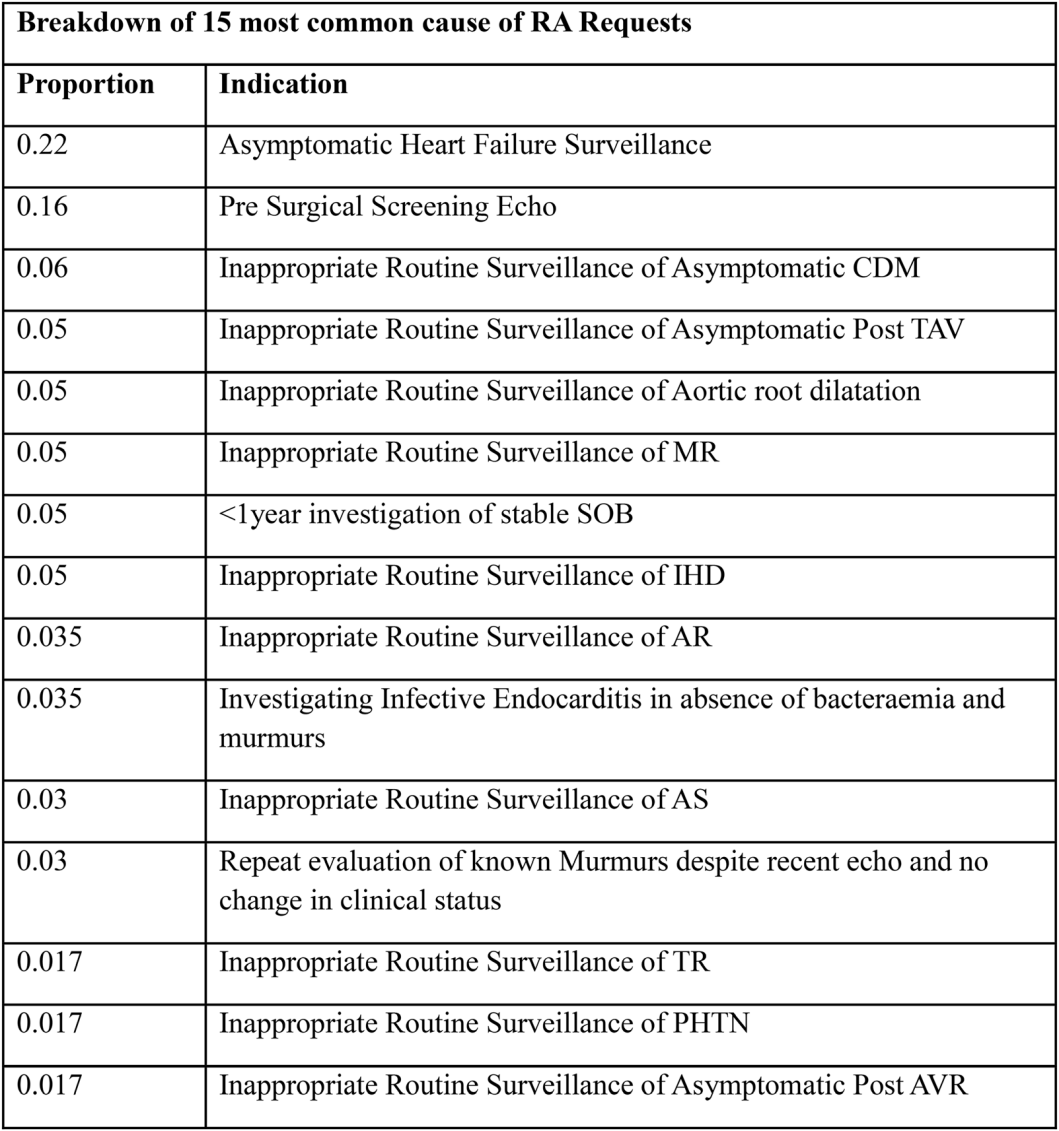
Breakdown of top 15 most common causes of RA requests

## ABS047 Echocardiographic measures of cardiac filling during heads-up tilt investigation of syncope

Amara Thanamayooran^1,2^, Mohamed Zuhair^1,2^, Cristian Ng^1,2^, Phil Eardley^1^, Mel Dani^1^, Patricia Taraborrelli ^1^, Phang Boon Lim ^1,2^, Darrel Francis^1,2^, Daniel Keene^1,2^, Matthew Shun-Shin^1,2^

^1^Imperial College Healthcare NHS Trust, London UK, ^2^Imperial College London, UK

*Echo Research & Practice 2026*, **13(Suppl 1):**ABS047

**Background** Vasovagal syncope (VVS) is defined as a transient loss of consciousness. It affects up to 50% of people, but the precise individual hemodynamic processes remain poorly understood. The heads-up tilt test (HUTT) is commonly employed to induce and investigate VVS. However, real-time valvular inflow dynamics during VVS are not documented.

**Purpose** To identify patterns of cardiac filling and stroke volume and how they may differ between syncope and control groups.

**Methods** Twenty patients with unexplained syncope completed a HUTT following the Italian protocol. Patients that had syncope were labelled as tilt-positive while non-syncope patients were labelled as tilt-negative. Specific time points were analysed: Supine, Passive tilt, GTN and End of Test (time of syncope in tilt-positive group, end of HUTT in tilt-negative group). Pulsed-wave Doppler recorded mitral and tricuspid inflow, and LVOT velocity-time integrals (VTI). Tilt-positive and tilt-negative groups were compared using the two-sided Wilcoxon rank-sum test.

**Results** MV E-wave decreased in both tilt-positive and tilt-negative groups, with a greater decline seen in tilt-positive patients−43 ± 10% (Fig. 1A, p = 0.002). In the tilt-negative group, MV A-wave increased−52 ± 14%, while the tilt-positive group decreased −12 ± 8% (Fig. 1B, p = 0.001). Reductions in all valvular VTIs were observed across phases in both groups (Fig. 2, p > 0.05).

**Conclusion** Continuous echocardiographic monitoring during HUTT revealed a greater reduction in passive filling, combined with a lack of increase in compensatory atrial “kick” in the tilt-positive group, despite overall reductions in inflow VTI remaining the same in both groups.

**Fig. 1(abstract ABS047):**
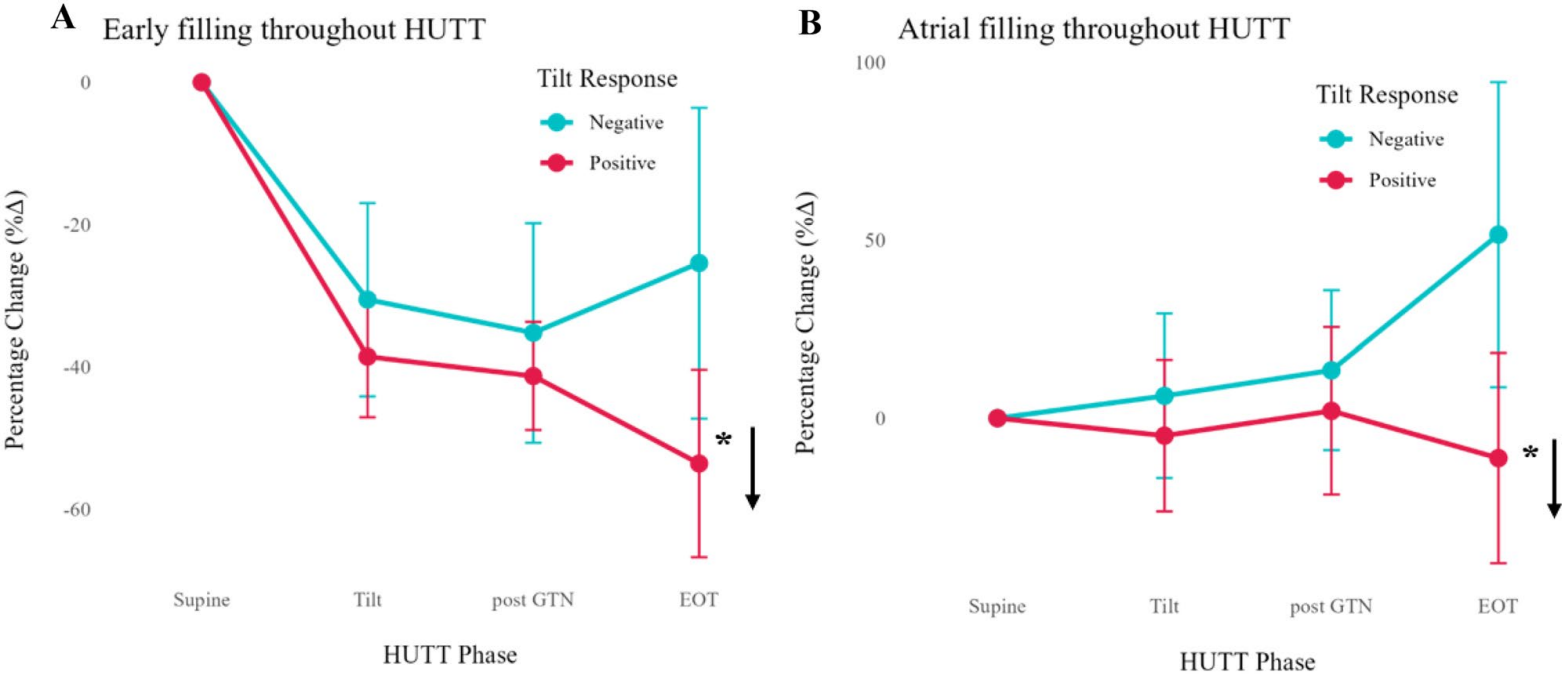
Percentage change of mitral early and atrial filling across HUTT

**Fig. 2(abstract ABS047):**
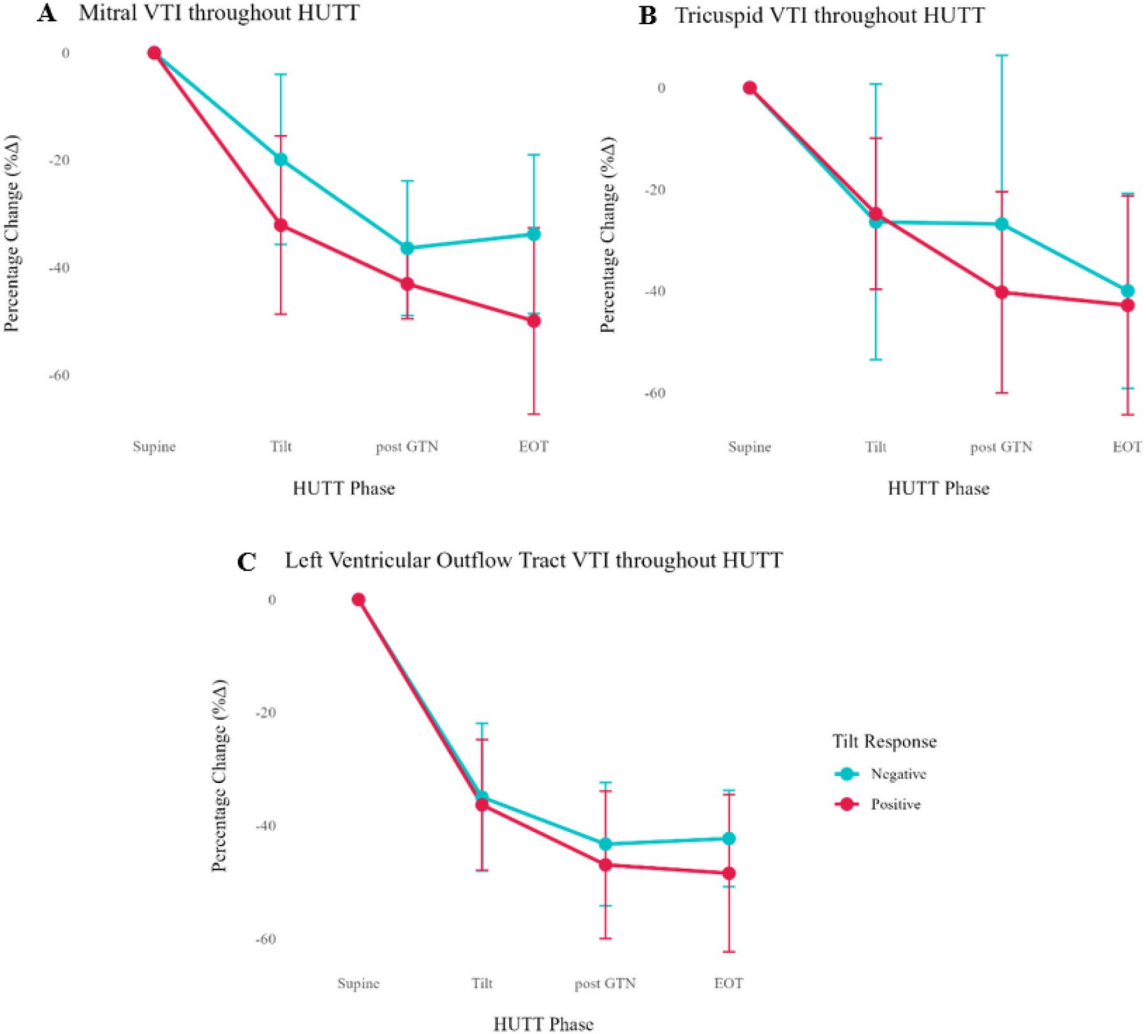
Percentage changes of valvular VTI’s across HUTT

## ABS048 Ventricular-vascular coupling in patients with hypertrophic cardiomyopathy

Ernestene Vasa^1, 2^, Jie Shen^1^, Louise Keehn^1^, Gerry Carr-White ^2^, Phil Chowienczyk^1, 2^, Haotian Gu ^1, 2^

^1^King’s College London, London, UK, ^2^Guy’s and St Thomas’ NHS Foundation Trust, London, UK

*Echo Research & Practice 2026*, **13(Suppl 1):**ABS048

**Background** In patients with hypertrophic cardiomyopathy (HCM), particularly those with obstructive HCM (HOCM), ventricular-vascular coupling (VVC) is often abnormal due to abnormal intrinsic changes in both ventricular haemodynamic and vascular function. However, VVC during early systole prior to the start of obstruction remains unclear.

**Purpose** This study aims to explore the association between early left ventricular (LV) contraction as measured by first-phase ejection fraction (EF1) and central aortic pressure waveforms as measured by augmentation pressure (cAP) and central augmentation index (cAIX).

**Methods** Sixty-four patients (HCM=30, HOCM=34) were prospectively recruited from the Inherited Cardiac Conditions clinic at St Thomas’ Hospital. EF1, the proportion of LV volume ejected from the onset of systole to the time of peak aortic flow velocity, was measured from echocardiography. cAP and cAIX, the difference between the first peak and second pressure peak, was measured by SphygmoCor (Fig. 1).

**Results** General characteristics are shown in Table 18. Patients with HOCM had increased filling pressure as measured by E/e’ compared to those with HCM. EF1 was significantly higher (35.4±8.9% vs 27.4±7.7%, p<0.001), whereas cAP and cAIX were lower (cAP:2.0±10.5mmHg vs. 8.3±6.3mmHg, p=0.011; cAIX=5.1±21.1% vs.20.0±12.5%, p=0.003) in HOCM patients compared to HCM (Table 18). Decreased cAP and cAIX were associated with a higher EF1, independent of age, sex, and mean arterial pressure (cAP:β=-0.32, p=0.046; cAIX: β=-0.38, p=0.013).

**Conclusion** Early systolic ventricular-vascular coupling differs in HOCM, with higher EF1 and lower cAP and cAIX compared to HCM. These findings suggest enhanced early LV contraction may reduce central wave reflection prior to obstruction onset.

**Fig. 1(abstract ABS048):**
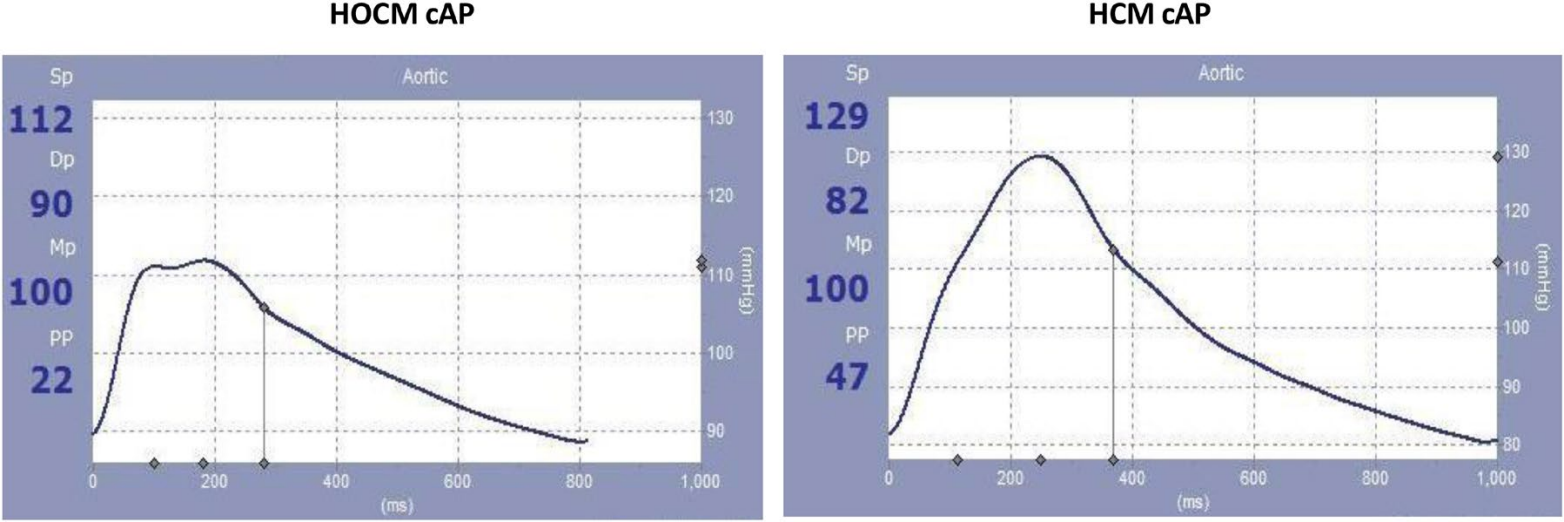
First and second pressure peaks. An example of central aortic pressure waveforms illustrating the difference between the first and second systolic pressure peaks in a patient with HOCM compared to a patient with HCM. Abbreviations: Sp = systolic pressure; Dp = diastolic pressure; Mp = mean pressure; PP = pulse pressure

**Table 18:** Demographics, echocardiographic and pulse wave analysis measurements between patients with HCM and HOCM

	HCM	HOCM	*P value*
	(n=30)	(n=34)	
Sex (M/F)	21/9	21/13	0.489
Age(y)	57.1±15.5	58.0±14.1	0.813
Weight(kg)	86.3±18.8	83.8±19.9	0.603
BSA (m^2^)	1.99±0.23	1.96±0.30	0.757
LVEDV (ml)	104.3± 27.1	98.8±33.7	0.476
LV RWT	0.69±0.14	0.69±0.20	0.985
LVM(g)	256.3± 96.2	232.8±76.0	0.280
LVMI (g/m^2^)	129.0±43.9	118.7±39.1	0.336
LVEF (%)	55.8±7.7	59.1±7.5	0.087
LVEF1 (%)	27.4±7.6	35.4±9.0	**<0.001**
E/e’	9.8±4.4	13.5±6.6	**0.014**
LAV(ml)	82.7±44.3	89.8±37.1	0.488
pSBP(mmHg)	133.9± 18.6	137.0±15.4	0.506
pDBP(mmHg)	78.4± 11.2	79.3 ±10.2	0.749
MAP(mmHg)	96.9±13.5	97.7±11.2	0.808
cSBP(mmHg)	119.9±17.5	122.6±16.7	0.571
cDBP(mmHg)	79.6 ±11.8	79.6±10.3	0.994
cAP	8.4±6.4	2.0±10.5	**0.011**
cAIX	20.0±12.5	5.2±21.1	**0.003**

Data shown are presented as mean±standard deviation. A two-tailed student t-test was done with a p-value of <0.05 indicating statistical significance

## ABS050 Is right heart remodelling prevalent and progressive in people receiving a pacemaker?

Chelsea Reji^1^, Nurul H. Abdul Samad^1^, John Gierula^1^, Judith E. Lowry^1^, Sam Straw^1^, Richard M. Cubbon^1^, Klaus K. Witte^1^, Maria F. Paton^1^

^1^Leeds Institute of Cardiovascular and Metabolic Medicine, Multidisciplinary Cardiovascular Research Centre, University of Leeds, Leeds, UK

*Echo Research & Practice 2026*, **13(Suppl 1):**ABS050

**Introduction** Whilst left ventricular (LV) systolic dysfunction remains the predominant concern, right ventricular (RV) remodelling and worsening tricuspid regurgitation (TR) are infrequent but important complications of RV pacing, associated with adverse outcomes, including right heart failure, hospitalisation and mortality. However, pacemaker-related RV remodelling on echocardiography remains unclear.

**Aim** To explore echocardiographic-derived right heart measurements in patients receiving de novo pacemakers for bradycardia.

**Methods** Patients from 3 UK centres receiving permanent pacemakers (2013-2016), with adequate echocardiographic images for right heart assessment were included. Data was prospectively collected with consent (IRAS:12/YH/0487). Guideline variations prompted exploration of multiple RV global longitudinal strain (GLS) impairment definitions. Descriptive statistics and paired t-tests were used (IBM: SPSS Statistics, Version 29).

**Results** Fifty-three patients were included (72% male, age 76±9 years) with common comorbidities including atrial fibrillation (13%), type 2 diabetes (10%), prior myocardial infarction (10%) and percutaneous coronary intervention (45%) (Table 19). Baseline right heart size and function were mostly normal, although 95% of patients had RV systolic dysfunction defined by GLS –23%, and 79% using –20% (BSE and ESC guidelines, respectively). After a median follow-up of 12 (IQR:11–12) months, paired data from 13 patients showed a significant increase in RA major diameter (Δ of 4.15±6.00mm; p=0.02), with statistically significant decrease in RV S’ (Δ of 0.03±0.01mm; p=0.04) (Table 20). One patient was hospitalised for heart failure with no deaths.

**Conclusion** Most pacemaker recipients have normal right heart size and function by traditional measurements, but reduced RV GLS is common, with unknown clinical significance. Although RA major diameter and RV S’ velocity reached statistical significance, the limited sample size restricts firm clinical conclusions. Larger studies are recommended to validate these findings.

**Table 19:** Baseline demographics

		**Total Cohort (n=53)**
Age (years)		76 (±9)
Sex (male)		38 [72%]
Height (cm)		169 (±10)
Weight (kg)		79 (±21)
Resting Heart rate (bpm)		68 (±12)
Systolic blood pressure		141 (±18)
Diastolic blood pressure		74 (±9)
Co-morbidities		
	Atrial fibrillation	25 [13%]
	Type II Diabetes Mellitus	19 [10%]
	Cerebrovascular accident	11 [6%]
Overt ischaemic heart disease		
	MI	19 [10%]
	PCI	8 [45%]
	CABG	15 [8%]
Blood investigation		
	NT-proBNP (pg/ml)	600 (166-1145)
Medications		
	Beta-blocker	23 [43%]
	Ace-inhibitor	21 [40%]
	Diuretic	14 [26%]
Pacing system		
	Dual chamber pacing	39 [74%]
Pacing programming		
	DDD (R)	20 [38%]
	RV pacing avoidance algorithm (R)	17 [32%]
	VVI (R)	13 [25%]
	Rate response	10 [19%]
	Base rate (bpm)	49 (±3)
	Max tracking rate (bpm)	129 (±9)
Traditional Echocardiographic measurements		
	LVEF (%)	51 (±8)
	RV Length (mm)	70 (±12)
	RV Mid (mm)	31 (±6)
	RV Base (mm)	38 (±7)
	RA Major (mm)	56 (±10)
	RA Minor (mm)	43 (±9)
	Proximal RVOT (mm)	32 (±5)
	Distal RVOT (mm)	21 (±5)
	TR jet (m/s)	2 (±0.3)
	PASP (mmHg)	32 (±8)
	RV EDA (cm^2^)	15 (±5)
	RV ESA (cm^2^)	9 (±3)
	RV FAC (%)	40 (±5)
	TAPSE (mm)	21 (±4)
	S’ Velocity (m/s)	0.14 (±0.02)
Advanced Echocardiographic measurements (Strain)		
	LA S_CD (%)	-12 (±7)
	LA S_CT (%)	-6 (±7)
	LA S_R (%)	16 (±11)
	RV GLS (%)	-17 (±4)

Continuous data are presented as mean (± standard deviation) or median (interquartile range) and categorical data are presented as n (%). A p-value ≤0.05 was considered significant. MI; myocardial infarction, PCI; percutaneous coronary intervention, CABG; coronary artery bypass grafting, NT-proBNP; N-terminal pro-B-type natriuretic peptide, ACE inhibitor; Angiotensin-converting enzyme, LVEF; left ventricular ejection fraction, RV; right ventricle, RA; right atrium, RVOT; right ventricular outflow tract, TR; tricuspid regurgitation; PASP; pulmonary artery systolic pressure, EDA; end diastolic area, ESA; end systolic area, FAC; fractional area change, TAPSE; tricuspid annular plane systolic excursion, LA; left atrium, S_CD; conduit strain, S_CT; contractile strain, S_R; reservoir strain, GLS; global longitudinal strain

**Table 20:** Echocardiographic measurements at baseline and follow-up (n=13)

	Baseline (6 week)	Follow-up (12 months)	Delta Change (∆)	p-value
**Traditional Echocardiographic measurements**				
LVEF (%)	50 (±13)	43 (±14)	-7 (±8)	0.07
RV Length (mm)	66(±20)	71 (±8)	+4.15 (±18)	0.44
RV Mid (mm)	31(±5)	33 (±7)	+1.85 (±8)	0.42
RV Base (mm)	37 (±5)	38 (±6)	+0.85 (±5)	0.56
RA Major (mm)	53 (±8)	57 (±9)	+4.15 (±6)	0.02*
RA Minor (mm)	39 (±7)	43 (±8)	+3.77 (±8)	0.10
Proximal RVOT (mm)	32 (±7)	33 (±5)	+1.15 (±5)	0.44
Distal RVOT (mm)	21 (±7)	19 (±2)	-1.44 (±7)	0.54
TR jet (m/s)	2.7 (±0.4)	2.7 (±0.6)	+0.02 (±0.36)	0.91
PASP (mmHg)	38 (±11)	39 (±17)	+0.72 (±8)	0.85
RV EDA (cm^2^)	15 (±3)	16 (±4)	+0.77 (±4)	0.49
RV ESA (cm^2^)	9 (±2)	10 (±4)	+1.11 (±3)	0.18
RV FAC (%)	40 (±6)	37 (±7)	-2.8 (±7)	0.15
TAPSE (mm)	20 (±3)	19 (±3)	-1.62 (±4)	0.18
S’ Velocity (m/s)	0.14 (±0.01)	0.11 (±0.02)	-0.03 (±0.01)	0.04*
**Advanced Echocardiographic measurements (Strain)**				
LA S_CD (%)	-12 (±7)	-13 (±7)	+0.55 (±8)	0.82
LA S_CT (%)	-7 (±9)	-7 (±7)	-0.9 (±10)	0.99
LA S_R (%)	15 (±16)	19 (±11)	+4.55 (±18)	0.43
RV GLS (%)	-18 (±4)	-19 (±3)	+0.23 (±4)	0.87

Values are presented as mean (± standard deviation). A p-value ≤0.05 was considered significant. LVEF; left ventricular ejection fraction, RV; right ventricle, RA; right atrium, RVOT; right ventricular outflow tract, TR; tricuspid regurgitation; PASP; pulmonary artery systolic pressure, EDA; end diastolic area, ESA; end systolic area, FAC; fractional area change, TAPSE; tricuspid annular plane systolic excursion, S_CD; conduit strain, S_CT; contractile strain, S_R; reservoir strain, GLS; global longitudinal strain

**Fig. 1(abstract ABS050):**
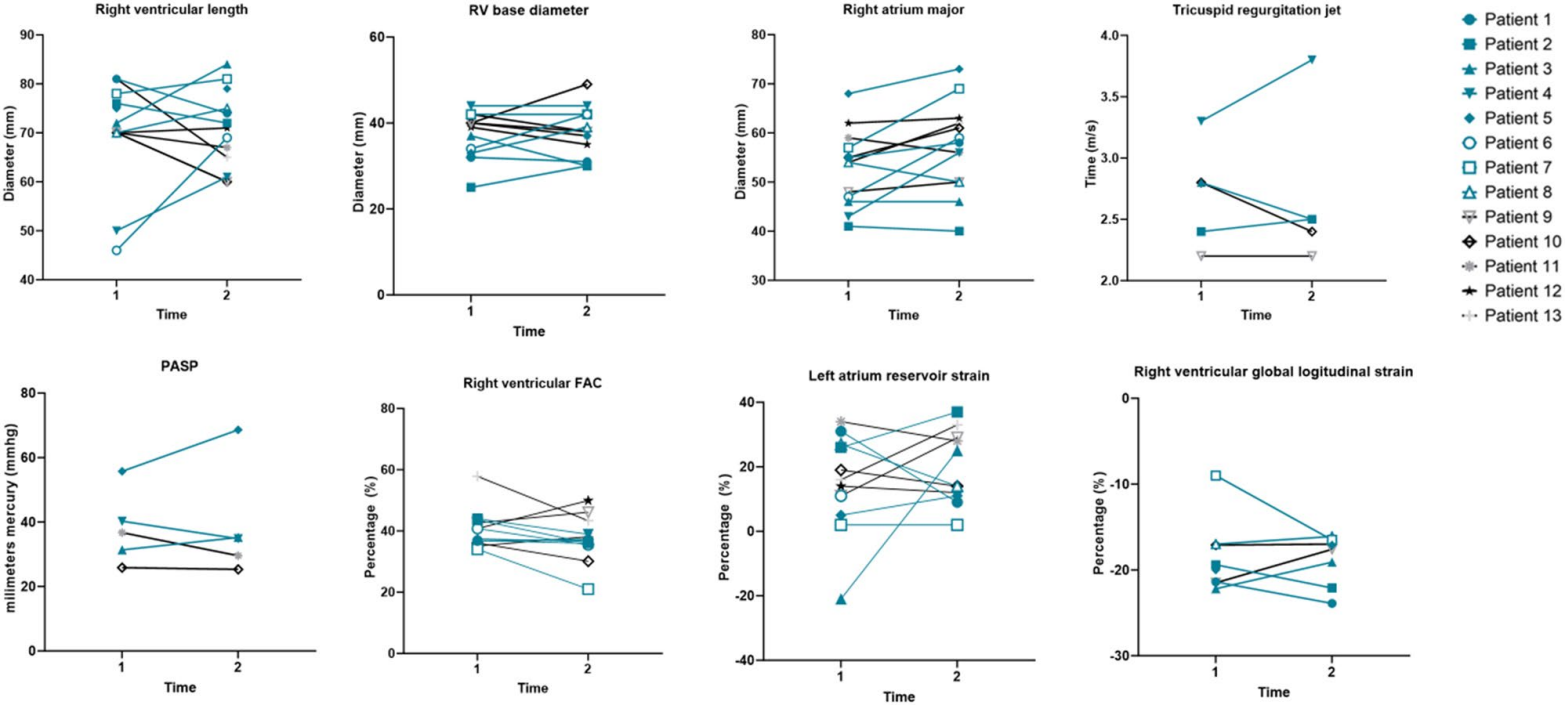
Line plots showing changes from baseline to follow-up in echocardiographic measurements (n=13)

## ABS052 Physiologist-led structural intervention clinic to identify early complications after percutaneous procedures

David Hoare^1^, Florence Lorenzo^1^, Rebecca Macrae^1^, Amit Bhan^1^

^1^Barts Heart Centre, St Bartholomew’s Hospital, London, UK

*Echo Research & Practice 2026*, **13(Suppl 1):**ABS052

**Background** Early (up to 6 months) complications after percutaneous closure of atrial septal defect (ASD) or patent foramen ovale (PFO) include device embolisation, erosion (causing pericardial effusion), arrhythmia and access site complications.^(1,2)^ Due to these risks, patients at our centre are reviewed 6 weeks post-procedure.

**Purpose** To evaluate the safety and feasibility of a physiologist-led follow-up clinic for patients 6 weeks after ASD/PFO closure, building on evidence that such models can increase capacity and reduce waiting times.^(3)^

**Method** We established a physiologist-led clinic for 6-week post-ASD/PFO closure follow-up, including echocardiogram, electrocardiogram and consultation. Clinics were held alongside the consultant who performed the procedure, allowing supervision and escalation when required.

**Results** Between 01/2024-01/2025, 88 patients were seen; 68 post-PFO closure, 19 post-ASD closure and 1 post stenting for coarctation of the aorta. 61% (n=54) were male, 39% (n=34) were female, mean age at time of procedure was 46 years old. 98.5% (n=66) of PFO closures were for previous stroke, 1.5% (n=1) were before complex neurosurgery. 75% (n=15) of ASD closures were due to right heart dilatation, 25% (n=5) were for previous stroke, Fig. 1. All patients had uncomplicated procedures. Palpitations were the most frequently reported symptom at follow-up, Table 21. No cases of device embolisation, significant pericardial effusion or puncture site complications were discovered. 1.1% (n=1) of patients were identified as non-compliant with antiplatelet therapy. 64% (n=56) of cases were discussed with the consultant in clinic, although it was fewer (43%) after 6 months of inception, representing increased physiologist autonomy.

**Conclusion** A physiologist-led clinic proved to be a safe and effective way to exclude significant early complications following percutaneous closure of ASD or PFO.

**Table 21:** Documented complications at the physiologist-led structural intervention clinic

88 patients	%	n=
Device embolisation	0	0
Pericardial effusion (>physiological)	0	0
Puncture site concerns	0	0
Compliant with antiplatelet therapy	98.8	87
Symptoms		
Palpitations	18.1	16
Breathlessness	9.1	8
Chest pains	4.5	4
Other, significant (listed below)	5.7	5
*Myocardial infarction not related to procedure*	1.1	1
*Increased bruising/bleeding*	1.1	1
*Headache/migraine*	1.1	1
*Probable left eye amaurosis fugax (temp loss of vision)*	1.1	1
*Recurrent dizziness on beta-blocker*	1.1	1

**Fig. 1(abstract ABS052):**
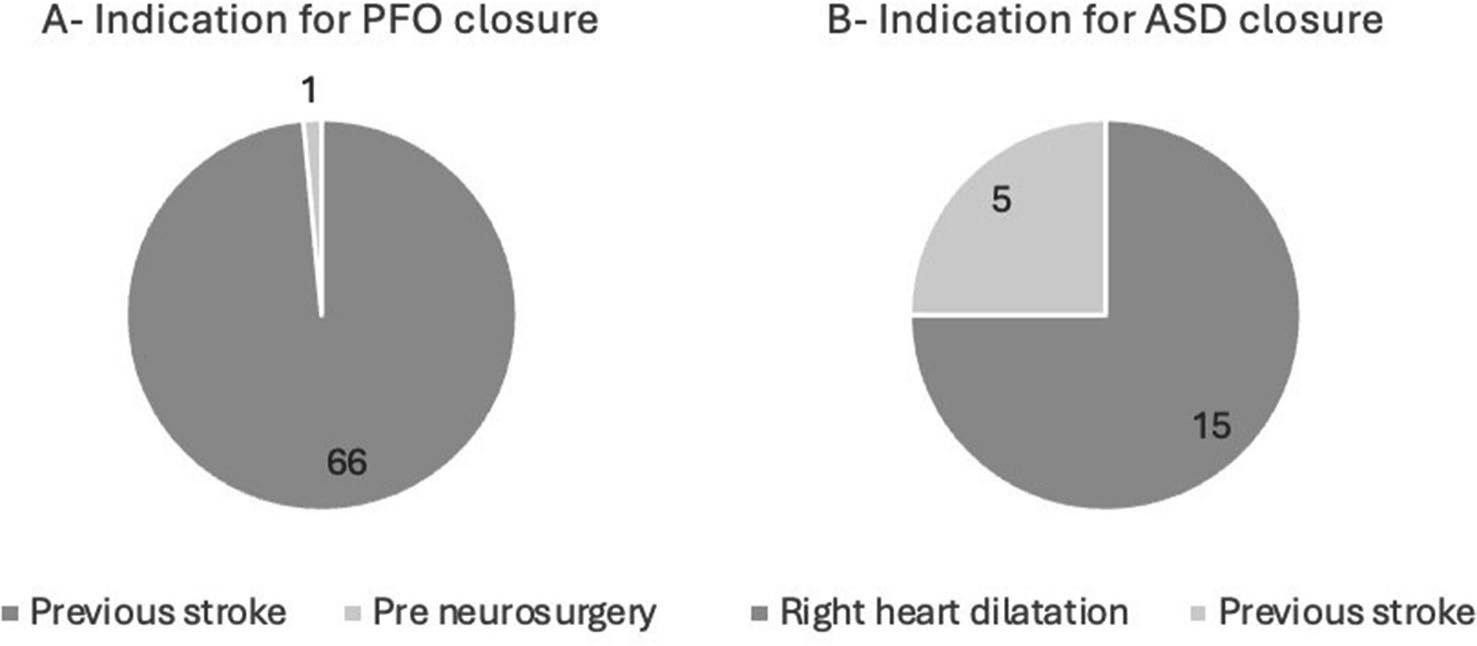
Indications for PFO/ASD closure in patients attending the physiologist-led structural intervention clinic. PFO, patent foramen ovale; ASD, atrial septal defect

**References**

1. Chessa M, Carminati M, Butera G, Bini RM, Drago M, Rosti L, et al. Early and late complications associated with transcatheter occlusion of secundum atrial septal defect. J Am Coll Cardiol. 2002 Mar 20;39(6):1061–5.
2. Wolf DD. Complications of transcatheter atrial septal defect closure. Interv Cardiol. 2009;1(2):209–18.
3. Sinclair H, Ackrill M, Holdsworth H, Chase C, Guillen M, Bowman L, et al. 88 Rapid access heart failure clinic: impact of a physiologist-delivered service in a uk district general hospital. Heart. 2019 May 1;105(Suppl 6): A74–A74.

## ABS053 How long does an echocardiogram take? An audit of transthoracic echocardiography scanning and reporting times in an adult population at a UK tertiary centre

Toby Park^1,2^, Maria Paton^2,3^, Hilary Jessop^2^

^1^Manchester Metropolitan University, UK, ^2^Leeds Teaching Hospitals NHS Trust, Leeds, UK, ^3^University of Leeds, Leeds, UK

*Echo Research & Practice 2026*, **13(Suppl 1):**ABS053

**Background** Transthoracic echocardiography (TTE) remains pivotal in the non-invasive assessment of cardiac structure and function, particularly for evaluating valvular and myocardial disease. The British Society of Echocardiography (BSE) currently recommends 40–45 minutes to perform a standard, comprehensive BSE- level scan. However, advancements in automated measurement tools, streamlined data transfer systems, and updates to the BSE minimum dataset may be influencing the efficiency and overall workflow of adult TTE services.

**Purpose** To audit if the adult cardiac ultrasound service within the Leeds Teaching Hospitals NHS Trust (LTHT) is aligned with the BSE’s guidance. To establish if specific workflow parameters impact scanning performance and reporting.

**Methods** Data was prospectively collected using a standardised form completed by echocardiographers for each scan performed from Monday to Friday over one week, commencing Monday 17 February 2025. Each form detailed the start and end time of each scan and the completion time of the corresponding report. Additional measured variables included referral reason, patient arrival time, timing of measurements, ultrasound machine manufacturer, time of day (AM or PM) and trainee presence. This audit was approved by LTHT. Ref: LOC0788.

**Results** Data from n=150 TTE scans from 22 echocardiographers across the LTHT, showed a mean scan time of 22 minutes and reporting time of 14 minutes, totalling 37 minutes (± 9 minutes) per echocardiogram and utilising 79% ± 20% of the allocated appointment. While scan time was not significantly affected by any measured variable, reporting time was significantly influenced by the presence of a trainee (p=0.012), time of day (p=0.049) and patient’s arrival time (p=0.008) (Table 22). Variation by referral reason is shown in Figs. 1 and 2.

**Conclusion** The adult cardiac ultrasound service at LTHT operates efficiently and in alignment with current BSE minimum dataset standards.

**Table 22:** T-test results showing the measured independent variables: (Training list (Trainee/No trainee), time of day (AM/PM), Late arrival (late/on time), timing of measurements (during/after/both), ultrasound machine manufacturer (Philips/GE), with the dependent variables of mean scan and mean reporting times

Variables	Total	Scan time mean (± SD)	P value	Report time mean (± SD)	P value
Training list	149				
Trainee	48	**22.83 (8.828)**		16.85 (7.410)	
			.523		.012
No trainee	101	21.96 (7.236)		13.60 (7.041)	
Time of the day	149				
AM	95	22.87 (8.271)		13.74 (6.477)	
			.188		.049
PM	54	21.13 (6.73)		16.20 (8.343)	
Late arrival	149				
Late	20	19.50 (7.626)		18.79 (10.664)	
			.90		.008
On time	129	22.67 (7.728)		14.03 (6.481)	
Timing of			Sig.		
measurements	148				
During	76	22.09 (7.957)			
After	23	23.13 (8.103)	.850		
Both	49	22.16 (7.476)			
Manufacturer	124				
Philips	60	22.55 (7.115)	.800		
GE	64	22.19 (8.624)

**Fig. 1(abstract ABS053):**
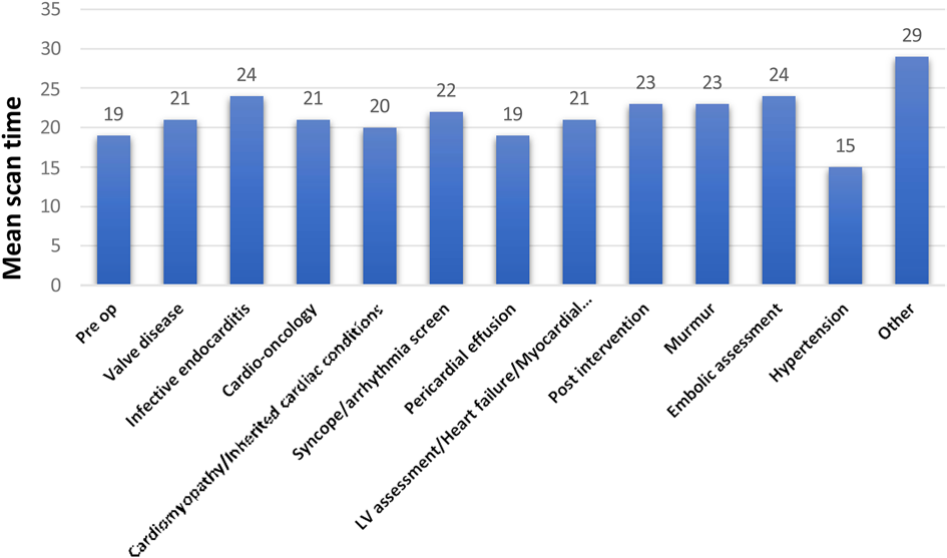
Mean Scan Time by Referral Reason. “Other,” “Infective endocarditis” and “embolic assessment” referrals required longer scan durations compared to “Hypertension,” “pericardial effusion” and “pre-operative” referrals, which had the shortest scan times

**Fig. 2(abstract ABS053):**
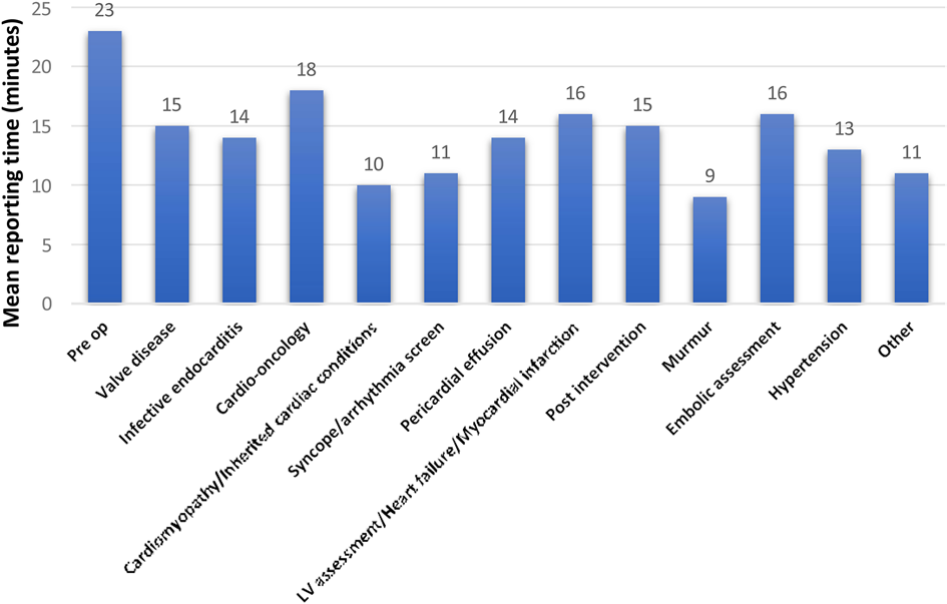
Mean reporting time by referral reason. “Pre-op,” and “cardio-oncology” referrals required longer reporting durations compared to “Murmur,” “cardiomyopathy/inherited cardiac conditions,” “syncope/arrhythmia screen” and “other” referrals taking the longest to report on

## ABS054 Evaluating left ventricular reverse remodeling in contemporary cardiac resynchronisation therapy patients using echocardiography

Tim Atkinson^1,2^Katie Finlay^1,2^, Nurul Abdul Samad^2,3^, Hillary Jessop ^2^, Maria Paton ^2,3^

^1^School of Healthcare Science, Manchester Metropolitan University, UK, ^2^Leeds Teaching Hospitals NHS Trust, Leeds, UK, ^3^University of Leeds, Leeds, UK

*Echo Research & Practice 2026*, **13(Suppl 1):**ABS054

**Background** Cardiac resynchronisation therapy (CRT) is a treatment for chronic heart failure (HF) characterised by left ventricular (LV) systolic dysfunction and ventricular dyssynchrony. Historically, “response” to CRT has been defined as a reduction in LV end systolic volume (LVESVi) of ≥15%, with up to 40% of patients failing to show benefit. The concept of “response” is flawed, and new paradigms suggest a spectrum of remission instead. In light of advancements in medical and device therapy, the degree of expected cardiac remodelling following CRT in contemporary clinical practice is unknown.

**Purpose** To determine the level of HF remission seen in people receiving CRT in a contemporary patient cohort.

**Methods** We performed a retrospective service evaluation of people with HF, receiving a CRT who were attending the Leeds CHAD service. Each patient underwent transthoracic echocardiography, 12-lead ECG and clinical assessment prior to implant, and this was repeated at 6-months post-implant. Remission was defined as a ≥15% reduction in LV end-systolic volume (LVESVi) from baseline to follow-up. The ability of baseline characteristics to predict the extent of remodelling at follow-up was assessed.

**Results** 41 patients were included; of these, 28 (68%) were male with a mean age of 71 (±10)yrs (Table 23). 83% of patients showed a ≥15% decrease in LVESVi at 6-month follow-up, meeting internationally defined criteria for remission. LVESVi decreased significantly from baseline to follow-up (86±5 mL/m^2^ vs 54±3 mL/m^2^ respectively, p< 0.01), LVEF increased significantly (26±7% vs 39±10% respectively, p<0.01) (Figs. 1 and 2). No clinical characteristic at baseline was associated with change in LVESVi at follow-up.

**Conclusion** 6-months of CRT alongside optimal medical therapy led to significant LV reverse remodeling. Remission was achieved in 83% of patients, considerably higher than in existing CRT literature. Echocardiography is key to assess HF remission and can be feasibly embedded within a combined HF and device service.

**Table 23:** Baseline patient characteristics and demographics. Continuous data expressed as mean ± SD and categorical data expressed as n (%) as indicated

Baseline patient characteristics (n = 41)	
Age, *years*	71 ± 10
Male sex, *n (%)*	28 (68%)
Height, *cm*	170 ± 10
Weight, *kg*	82 ± 19
*NYHA Classification*	
Class I, *n (%)*	6 (15)
Class II, *n (%)*	25 (61)
Class III, *n (%)*	8 (20)
Class IV, *n (%)*	1 (2)
*Existing diagnoses*	
IHD, *n (%)*	15 (36)
History of VT/VF, *n (%)*	2 (5)
Hypertension, *n (%)*	20 (48)
Diabetes, *n (%)*	15 (36)
COPD, *n (%)*	4 (10)
CKD, *n (%)*	8 (19)
*Medications*	
Prescribed β-blockers, *n (%)*	34 (83)
Prescribed ACE inhibitor, *n (%)*	18 (44)
Prescribed ARB, *n (%)*	2 (5)
Prescribed ARNI, *n (%)*	13 (32)
Prescribed SGLTII inhibitor, *n (%)*	28 (68)
Prescribed Diuretics, *n (%)*	34 (83)
**Baseline Haemodynamic Data**	
Resting heart rate, *beats min*^*-1*^	70 ± 13
Resting systolic blood pressure, *mmHg*	120 ± 19
**Baseline ECG Data**	
Sinus rhythm, *n (%)*	30 (73)
Atrial fibrillation, *n (%)*	9 (22)
Atrial tachycardia, *n (%)*	1 (2)
LBBB, *n (%)*	33 (81)
QRS duration, *ms*	161 ± 19
**Baseline TTE Data**	
LVEF, *%*	26 ± 7
LVEDVi, *mL/m*^*2*^	118 ± 35
LVESVi, *mL/m*^*2*^	87 ± 30

ECG, electrocardiogram; TTE, transthoracic echocardiogram; IHD, ischaemic heart disease; VT/VF, ventricular tachycardia/ventricular fibrillation; COPD, chronic obstructive pulmonary disease; CKD, chronic kidney disease; ACE, angiotensin-converting-enzyme; ARB, angiotensin receptor blocker; ARNI, angiotensin receptor neprilysin inhibitor; SGLTII, sodium-glucose co-transporter II; LBBB, left bundle branch block; LVEF, left ventricular ejection fraction; LVEDVi, indexed left ventricular end-diastolic volume; LVESVi, indexed left ventricular end-systolic volume

**Fig. 1(abstract ABS054):**
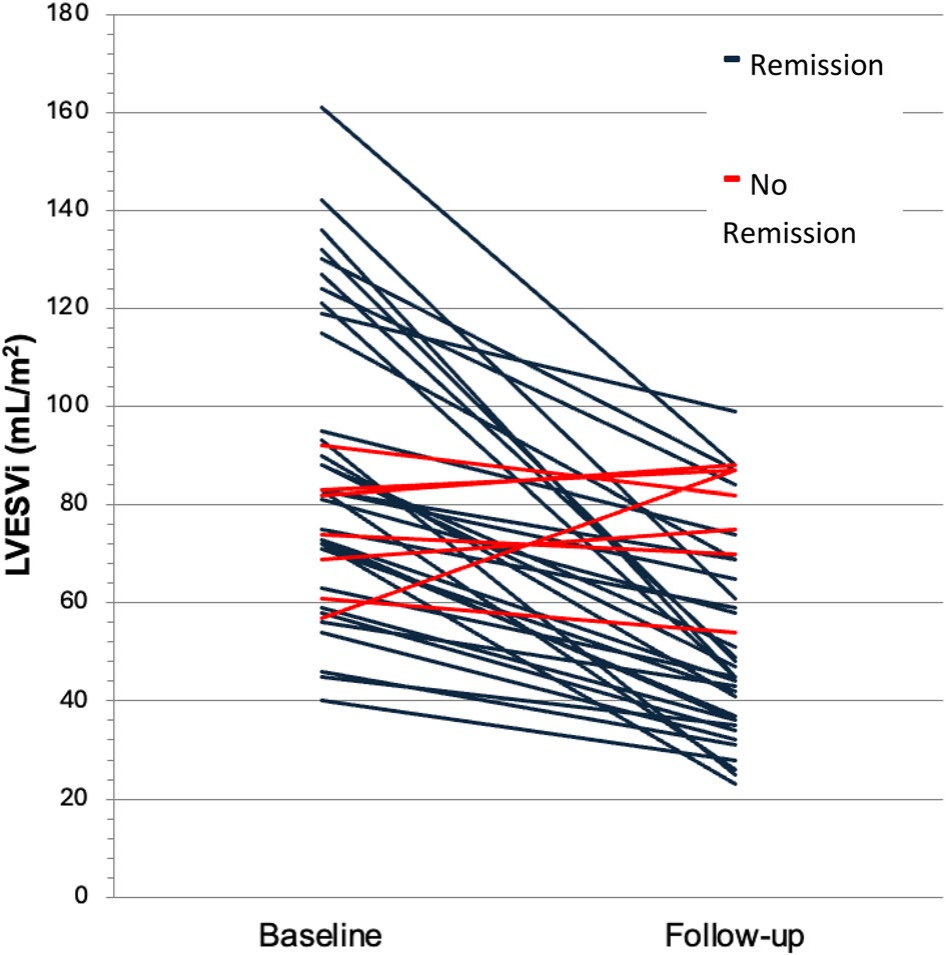
LVESVi change from baseline to follow-up grouped by remission status at 6-month follow-up. Remission is defined as a ≥15% decrease in LVESVi from baseline to 6-month follow-up. LVESVi, indexed left ventricular end-systolic volume

**Fig. 2(abstract ABS054):**
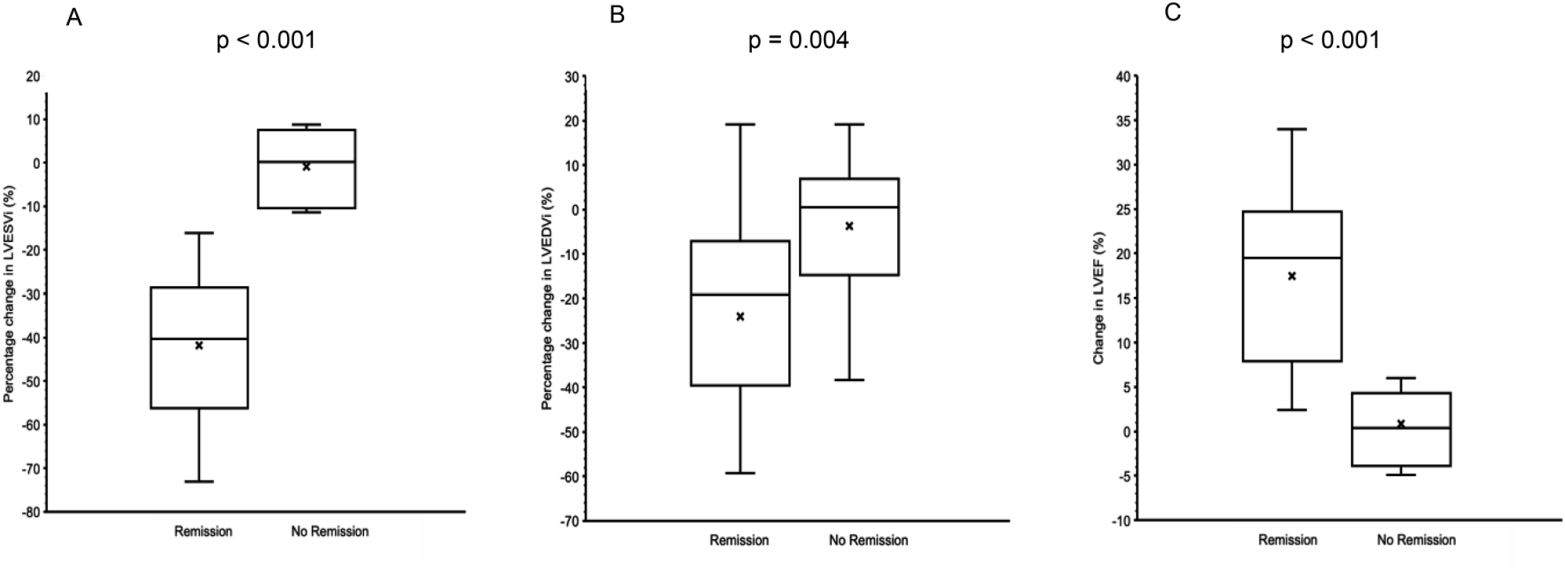
Differences in markers of LV reverse remodelling between patients showing remission and patients showing no remission at follow-up. Panel A – percentage change in LVEDVi from baseline to follow-up. Panel B – percentage change in LVEDVi from baseline to follow-up. Panel C – Percentage change in LVEF from baseline to follow-up. The boxes represent the interquartile range, with the central line indicating the median value. The mean value for each group is represented by the “x” marker and the whiskers represent the spread of data within each group. LVESVi, indexed left ventricular end-systolic volume; LVEDVi, indexed left ventricular end-diastolic volume; LVEF, left ventricular ejection fraction

## ABS055 Can educational interventions improve appropriate use of ultrasound contrast agents in routine echocardiography?

Sara Delgado^1^, Gavin McClean^1^, Joane Daradar^1^, Wei Li^1^, Rajdeep Khattar^1^, Roxy Senior^1^

^1^Echocardiography Department, Royal Brompton and Harefield Hospitals, Part of Guy’s and St Thomas’ NHS Foundation Trust, London, UK

*Echo Research & Practice 2026*, **13(Suppl 1):**ABS055

**Introduction** Contrast enhanced echocardiography can reduce the need for additional testing and expediate diagnosis (Fig. 1). Clinical use in rest echocardiography, however, remains low.

**Aim** To evaluate whether an educational intervention improves appropriate UCA utilisation.

**Methods** A retrospective, multi-cycle clinical audit was conducted using a before-and-after design following training and logbook submission for competency sign-off across three audit cycles (Fig. 2). 300 consecutive echocardiograms per cycle were evaluated for class I indications for and utilisation of UCAs per 2017 European Association of Cardiovascular Imaging (EACVI). UCAs were administered by a Clinical Fellow/Nurse. Comparisons utilised χ^2^ or Fisher’s exact tests. An anonymous questionnaire exploring barriers to UCA utilisation was distributed to all echocardiographers.

**Results** Significantly more echocardiography studies met class I indication for UCA in 2022 (24%) and 2024 (22%) than in 2023 (15%), respectively (P<0.05). Of the echocardiography studies that met class I criteria (Fig. 3), significantly more were graded as ‘green’ in 2023 (34%) vs. 2022 (18%) (P<0.05). Conversely, significantly more were graded as ‘amber’ in 2022 (43%) vs. 2023 (20%) and 2024 (28%), respectively (P<0.05). Of 23 respondents, 91% felt confident in determining when a UCA study is indicated, 49% in handling common UCA artefacts, and 26% felt that a 1-hour appointment was sufficient to undertake a UCA study. Barriers to UCA studies were attributed to time constraints posed by staff availability for cannulation and administration.

**Conclusion** Educational training and local competency sign-off did not result in a significantly sustained increased utilisation of UCA. To overcome barriers, a specialised echocardiographer could be assigned to review, cannulate and deliver UCAs across the department under the patient specific directive; forming a scientist-physiologist-led service.

**Fig. 1(abstract ABS055):**
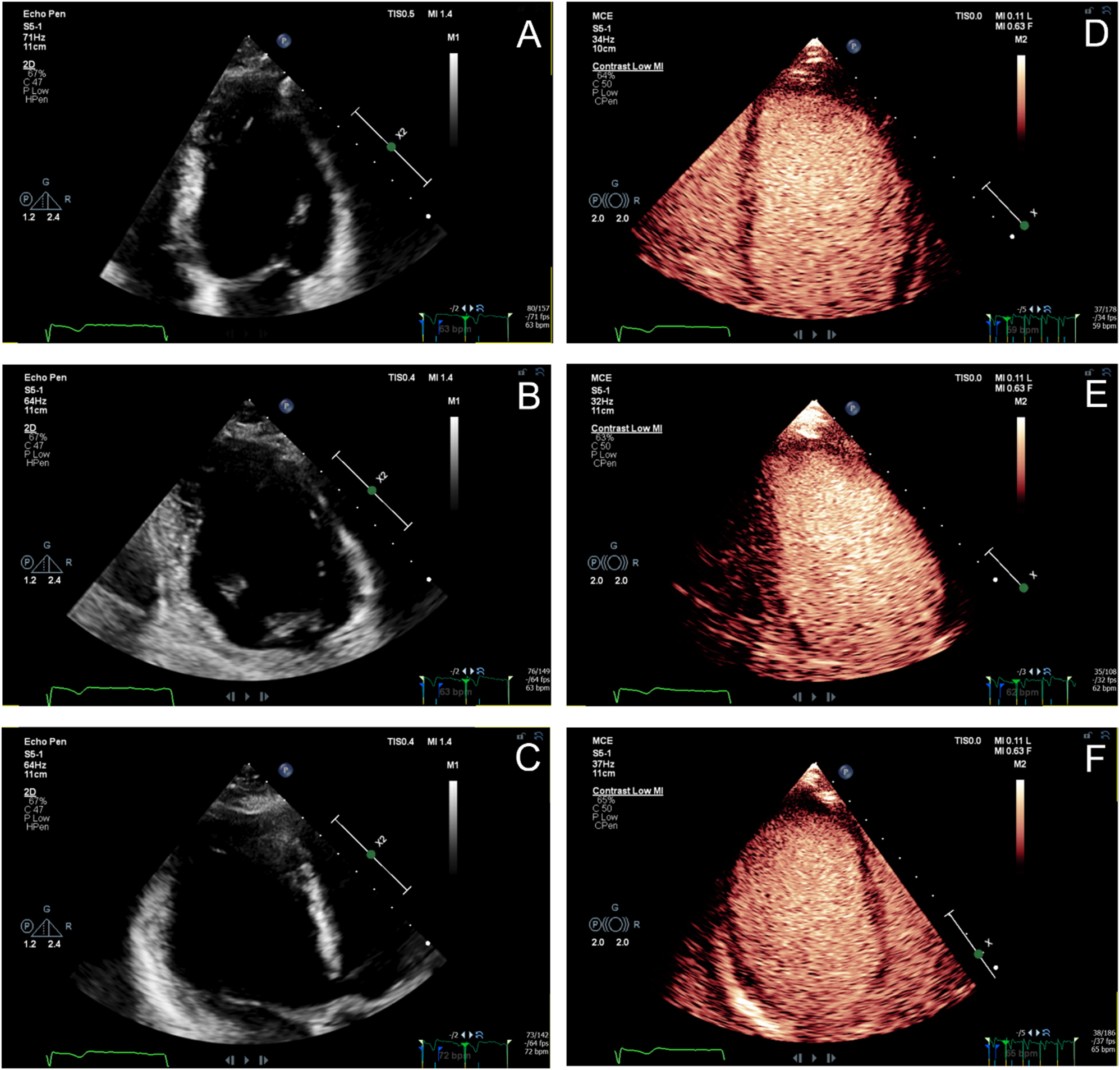
Unenhanced apical 4 chamber (A), 2 chamber (B) and 3 chamber (C) vs contrast enhanced apical 4 chamber (D), 2 chamber (E) and 3 chamber (F) at end-diastole in the same patient. Images used for the assessment of left ventricle ejection fraction for cardiac late effects in the setting of cardio-oncology

**Fig. 2(abstract ABS055):**
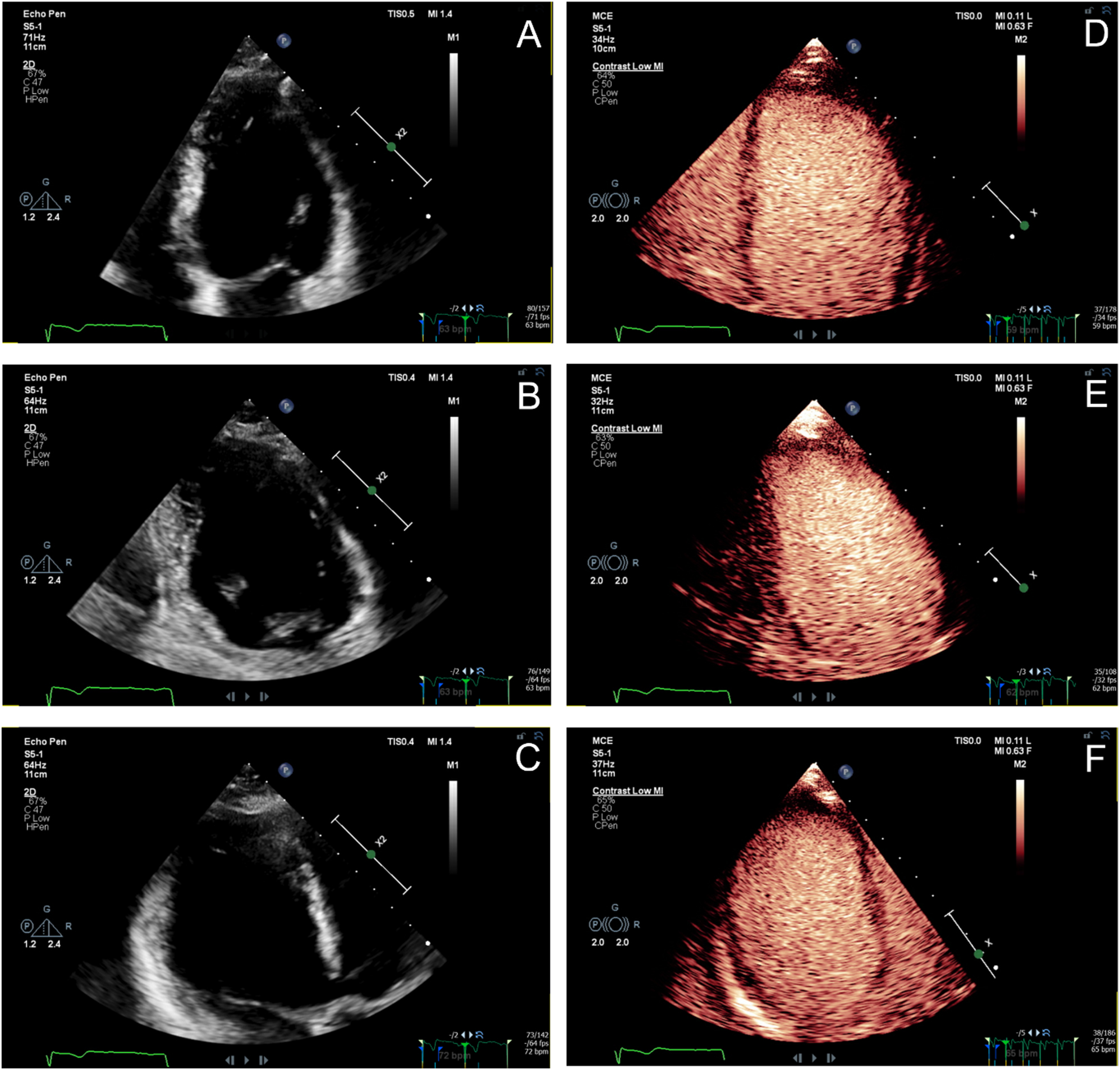
Flowchart summarising the study design and grading criteria for a multi-cycle audit assessing adherence to 2017 European Association of Cardiovascular Imaging (EACVI) Class I indications for ultrasound contrast agent (UCA) use. TTEs, transthoracic echocardiograms

**Fig. 3(abstract ABS055):**
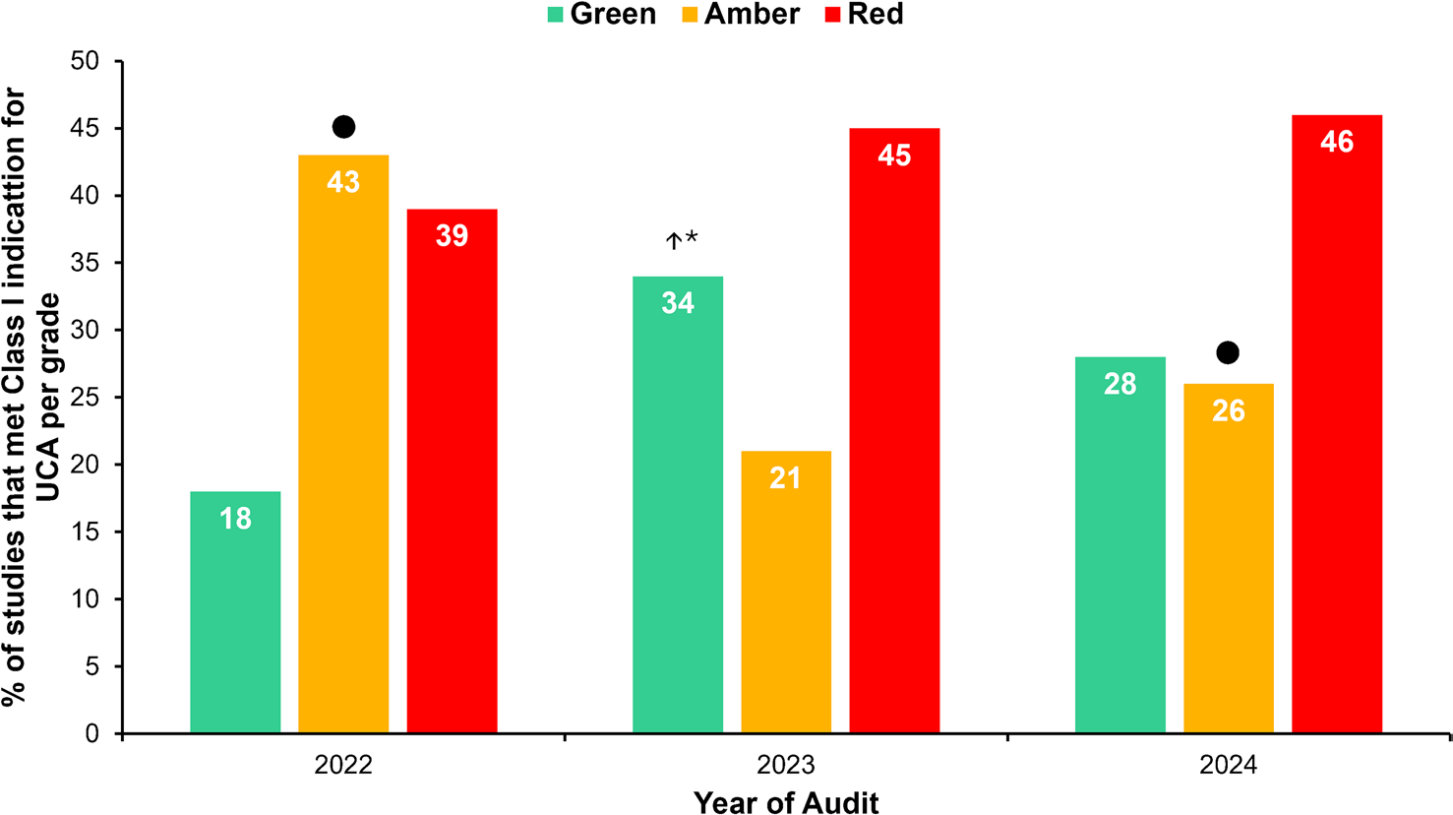
Bar chart of TTE studies that met class I indication for use of ultrasound contrast agent as per the 2017 European Association of Cardiovascular Imaging guidelines. Studies were graded as red, amber and green, respectively, with 300 consecutive studies analysed per audit year. ● Significantly more were graded as amber in 2022 than in 2023 and 2024, respectively (P<0.05); ↑* significantly more were graded as green in 2023 than in 2022 (P<0.05)

## ABS056 How often and when is contrast echocardiography indicated in the assessment of Hypertrophic Cardiomyopathy?

Gavin McClean^1^, Neeta Satawji^1^, James Malcolmson^2^, Joane Daradar^1^, Sara Delgado^1^, Roxy Senior^1^

^1^Echocardiography Department, Royal Brompton, Part of Guy’s and St Thomas’ NHS Foundation Trust, London, UK, ^2^Barts Heart Centre, St Bartholomew’s Hospital, London, UK

*Echo Research & Practice 2026*, **13(Suppl 1):**ABS056

**Introduction** Risk stratification for sudden cardiac death (SCD) in hypertrophic cardiomyopathy (HCM) involves echocardiography assessment of left ventricle max wall thickness (LVMWT), left atrial size, peak LV outflow gradient, LV ejection fraction (EF), and the presence/absence of an LV apical aneurysm (LVAA) as per the American Heart Association (AHA) SCD risk calculator. Echocardiography assessment, however, of such parameters is often technically challenging in HCM. Contrast-enhanced echocardiography (CEE) can overcome these challenges and reduce downstream testing costs.

**Aim** Determine the prevalence and indications for CEE in HCM patients, facilitating accurate assessment of AHA SCD risk parameters.

**Methods** Two BSE accredited echocardiographers independently reviewed 100 consecutive HCM studies from our tertiary centre (January-April 2025). Studies were evaluated for the potential benefit of CEE in assessing AHA SCD risk parameters, including those already using CEE. Consensus was reached in all cases.

**Results** Fifty-five percent of HCM studies would’ve benefited from CEE, of which, 23% received it. In 42% of cases LVAA was suspected but not clearly visualised or confidently excluded without CEE, and/or CEE was necessitated to assess for a thrombus (Figs. 1 and 2). Poor visualisation of ≥2 contiguous LV segments prevented accurate LVEF assessment in 32%. Apical hypertrophy was suspected but unclear in 30%. In 13%, basal-to-mid LVMWT was unclear in ≥2 segments (Fig. 3).

**Conclusions** Echocardiography assessment of HCM is complex; necessitating a comprehensive, multi-parametric approach. Over half of our studies would’ve benefitted from CEE, yet only 23% received it. We highlight the need to modify practice to improve test accuracy and risk stratification.

**Fig. 1(abstract ABS056):**
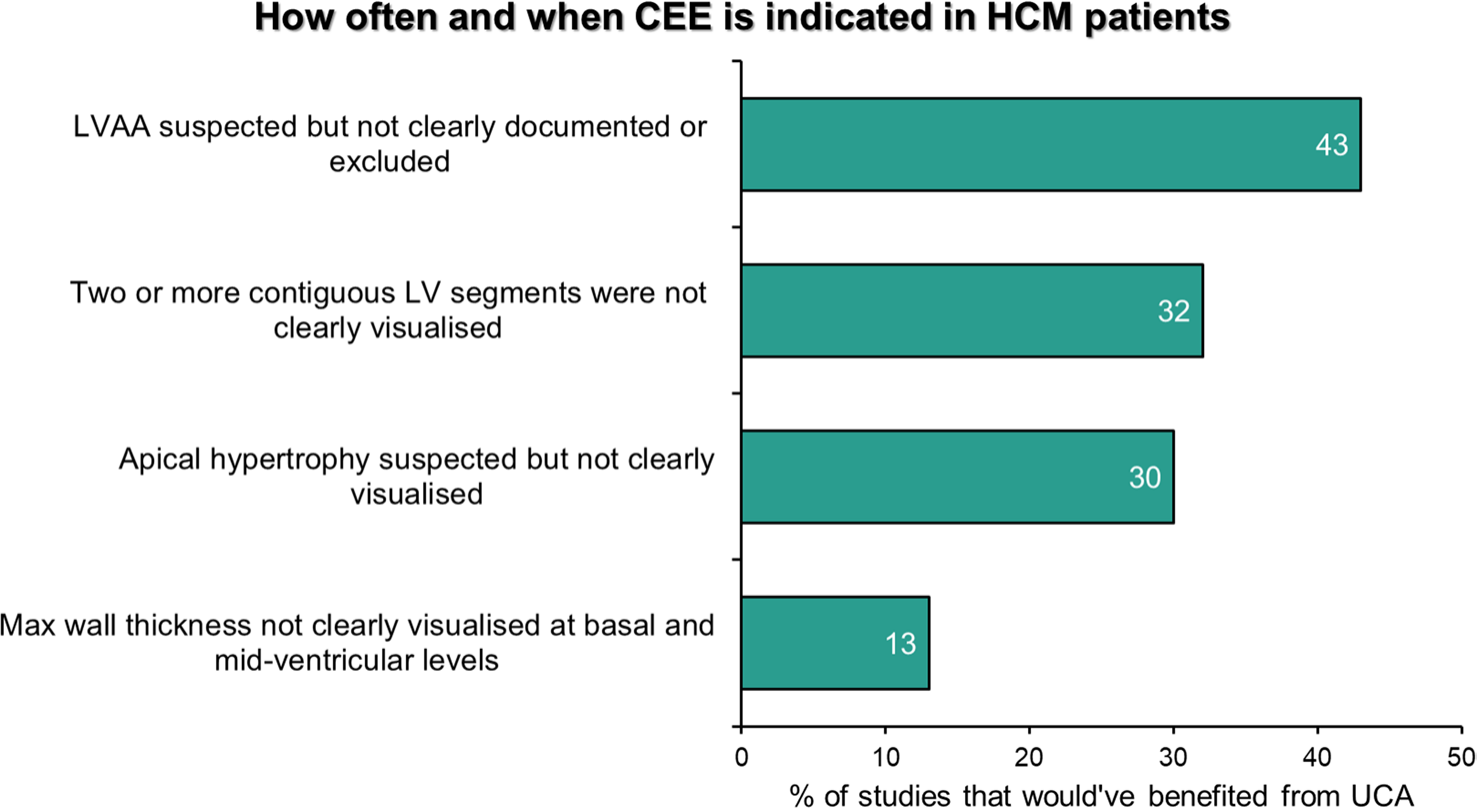
Bar chart illustrating how often and when contrast enhanced echocardiography (CEE) is indicated in the assessment of hypertrophic cardiomyopathy (HCM) patients. LVAA, left ventricle apical aneurysm

**Fig. 2(abstract ABS056):**
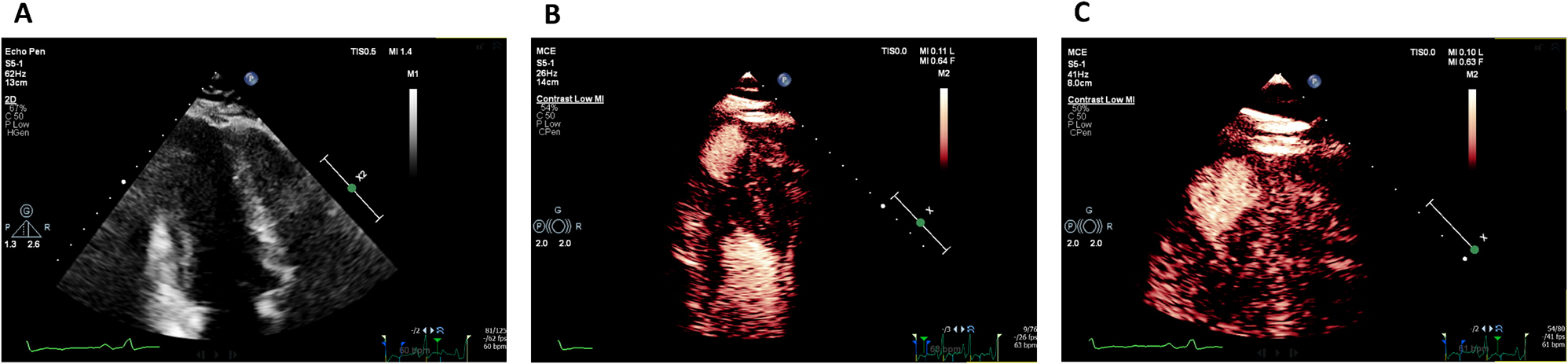
Unenhanced apical 3 chamber (A) vs contrast enhanced apical 3 chamber views (B & C) at end-systole in the same hypertrophic cardiomyopathy patient revealing a significant left ventricle apical aneurysm with no thrombus that was previously not visualised without the use of contrast enhanced echocardiography

**Fig. 3(abstract ABS056):**
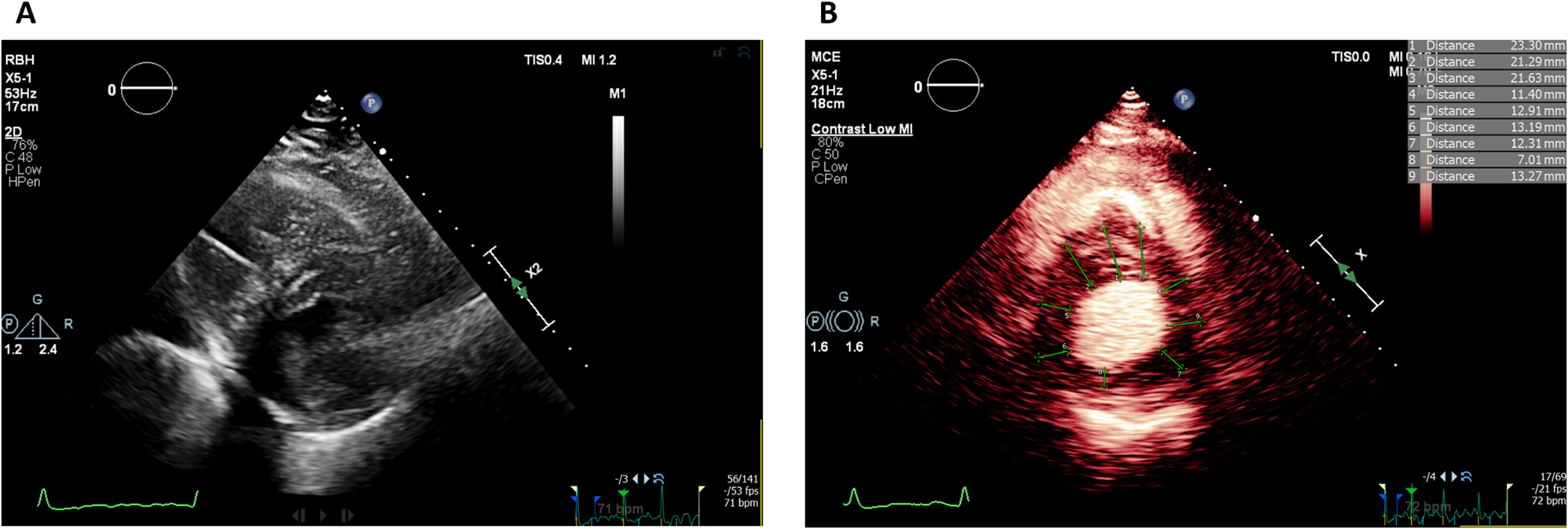
Unenhanced parasternal short axis (A) vs contrast enhanced parasternal short axis (B) at the mid-ventricular level in the same hypertrophic cardiomyopathy patient permitting accurate max wall thickness measurement of 23 mm at the mid-anteroseptal wall at end-diastole

## ABS057 Clinical outcomes of patients under surveillance post-aortic valve replacement in valve clinic

Chelsea Omeni-Nzewuihe ^1^, Robin Shome ^1^, Sitara Khan ^1^, Kirsty Bolter ^1^, Yaya Olufunke ^1^, Elnara Imanova ^1^

^1^Frimley Park Hospital, Frimley Health NHS Foundation Trust, Camberley, UK

*Echo Research & Practice 2026*, **13(Suppl 1):**ABS057

**Background** The 2024 BSE guidelines emphasise appropriate timeframes for prosthetic tissue valve surveillance, and consideration of patient factors to determine the appropriateness of ongoing surveillance^1^. We evaluated our valve clinic at Frimley Park Hospital, to ensure good resource utilisation and patient outcomes.

**Aim** To evaluate outcomes of post-aortic valve replacement (AVR) patients under valve clinic surveillance and adherence to 2024 BSE recommendations.

**Methods** A retrospective audit was conducted on 113 patients seen in the valve clinic between February 2023 and February 2024. Data on demographics, interventions, follow-up, and outcomes was analysed.

**Results** Median patient age was 77; time since AVR ranged from 2 months to 24 years (median 2.7 years). 109 (96.5%) continued under surveillance; 4 (3.5%) were discharged and of those discharged, 1 (0.88%) had moved out of area and 3 (2.7%) were discharged due to frailty. 2 patients (1.8%) developed endocarditis and 5 (4.4%) required emergency cardiac admissions. No deaths occurred during the audit period.

**Conclusions** Our patient group demonstrated favourable clinical outcomes, with low rates of endocarditis and unplanned cardiac admission. We only identified a few patients suitable for discharge. However, this was a retrospective audit that concluded prior to release of the 2024 BSE guidance. Going forwards, we will more proactively assess frailty in all valve clinic patients, to help improve resource utilisation further.

**References**

1. Clinical indications and triage of echocardiography: Heart valve disease. BSE poster 2024.

## ABS058 First year outcomes of an advanced clinical practitioner-led integrated valve clinic

Charles Spencer^1^, John Fryearson^1^

^1^SWFT – South Warwickshire University NHS Foundation Trust, UK

*Echo Research & Practice 2026*, **13(Suppl 1):**ABS058

**Introduction** The number of patients >65 years living with valvular heart disease in the UK is predicted to double to 3 million between 2015 and 2046, and has been dubbed the ‘next cardiac epidemic’ (d’Arcy 2011). Traditionally the surveillance of valve disease patients occurs in general cardiology clinics with timing of imaging at the discretion of the treating cardiologist. Anecdotally this approach has led to a number of delays to treatment and suboptimal coordination of imaging at Warwick Hospital, a medium sized district general hospital. There is evidence that specialist valve clinics improve patient outcomes and reduce the number of unnecessary echocardiograms (Ionescu, McKenzie et al. 2015).

**Purpose** To evaluate the first-year outcomes of a combined valve surveillance and echo clinic.

**Methods** We developed a ‘one stop’ valve surveillance clinic led by a single British Society of Echocardiography (BSE) Level 2 accredited Advanced Clinical Practitioner (ACP). A Plan, Do, Study, Act (PDSA) approach was used to enable a flexible approach to continuous improvement while running an initial 2-year trial focussing on those patients likely to benefit. Initially all images and cases were reviewed by the lead imaging cardiologist with a more selective approach used as the trial progressed. Patients were identified through a combination of direct referrals from cardiologists and screening of echo requests on the radiology booking system cross referenced with cardiology clinic access plan data. Patients meeting the clinic criteria who were due or overdue clinic follow-up were listed in the 4 x 1hr valve clinic slots per week. Inclusion criteria; patients with left-sided valve disease of at least moderate severity. Exclusion criteria; contraindications to intervention, prosthetic valves, significant aortic root dilatation, another primary cardiac complaint e.g. Heart Failure, Atrial Fibrillation. Data was collected prospectively on an excel spreadsheet including the type and severity of valve disease and clinic outcomes with reference to BSE guideline directed echo intervals (Bennett, Stout et al. 2022), and appropriate timing of intervention (Vahanian, Beyersdorf et al. 2021). Clinical outcomes were reviewed by electronic chart review 01/07/2025 (3-15 months post 1^st^ valve clinic slot).

**Results** The characteristics of patients seen in clinic between 01/04/2024 and 31/03/2025 are detailed in Table 24. There was wide variability in the time since clinic appointment and echocardiograms (Fig. 1) and waits for 58% of severe lesions were outside of the BSE recommended 6 months (±10%) (Table 24). Clinic outcomes are detailed in Fig. 2 including 6 cases of intervention, 5 TAVI and 1 mTEER. A further 2 patients are on the waiting list for surgical mitral valve repair, 1 has declined the offer of mTEER and 3 more are awaiting assessment at tertiary centres.

**Table 24(abstract ABS058):**
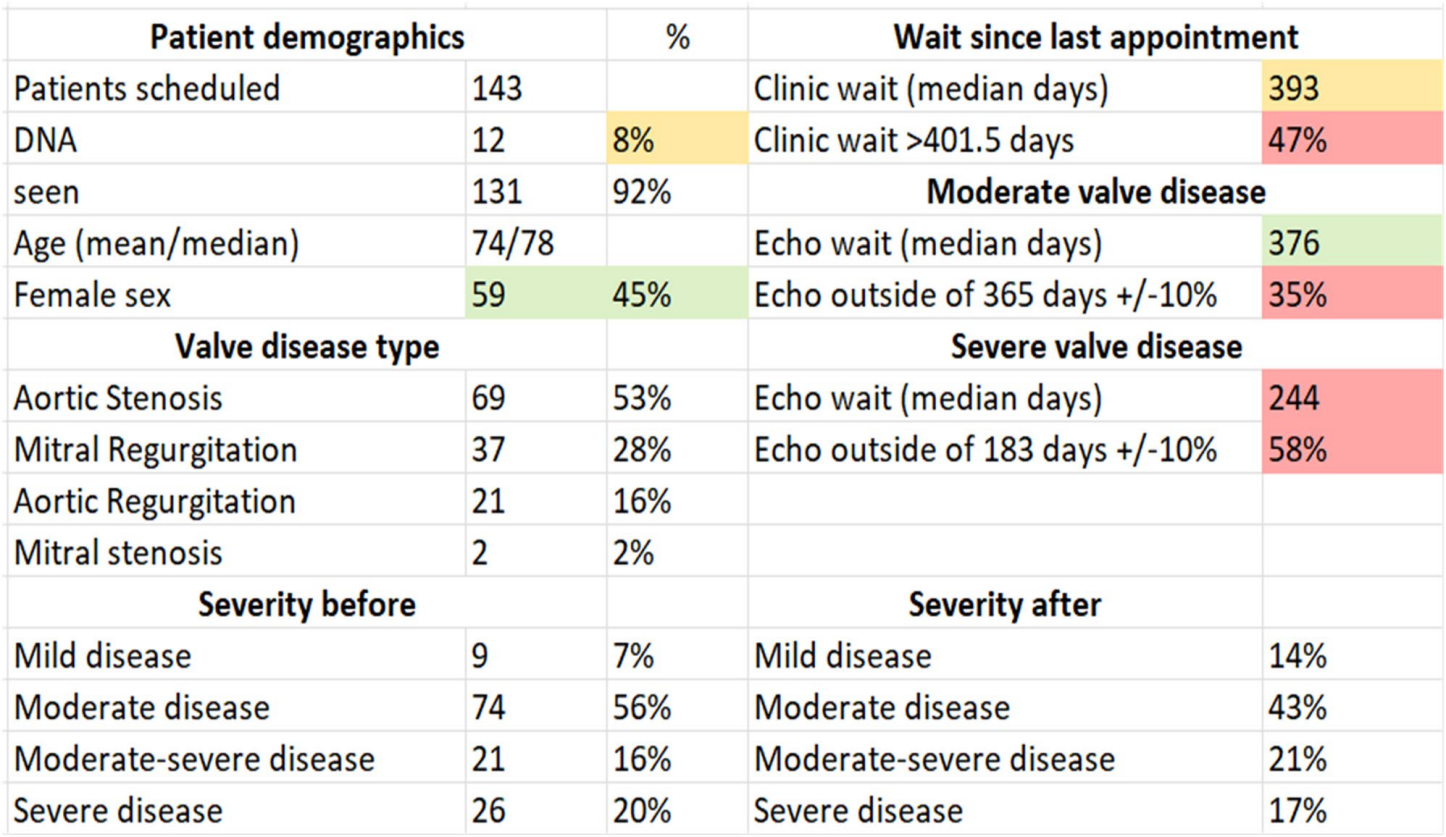
Patient demographics and average clinic waits

**Fig. 1(abstract ABS058):**
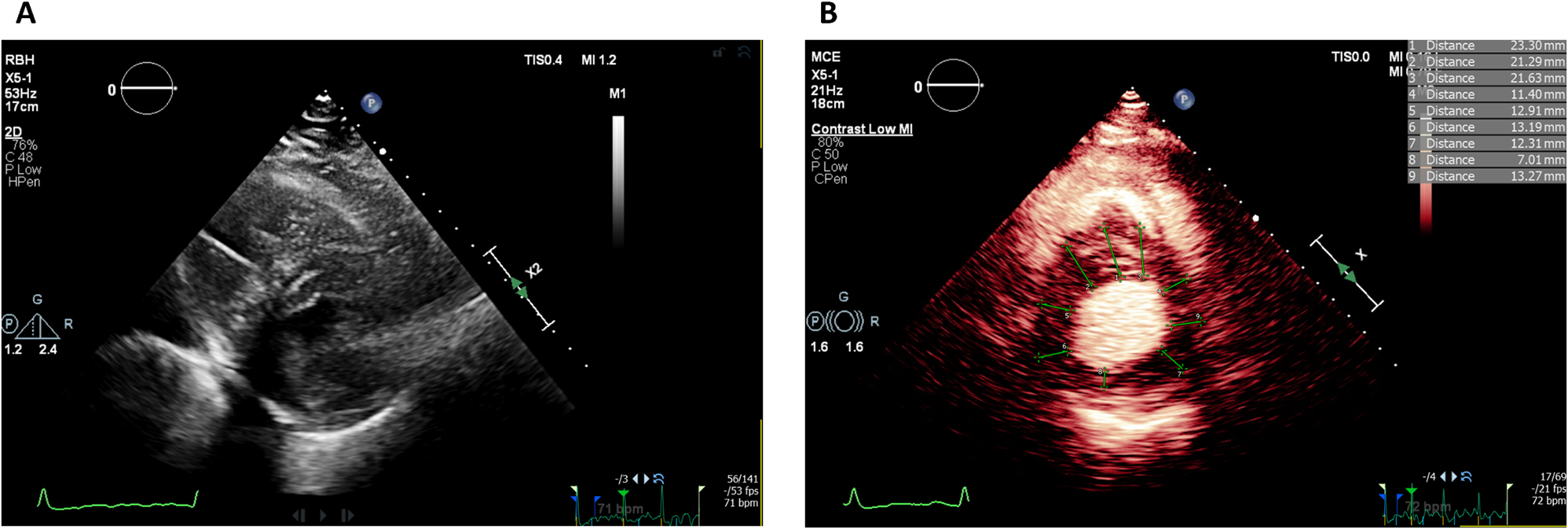
Outcomes and actions following valve clinic appointment

**Fig. 2(abstract ABS058):**
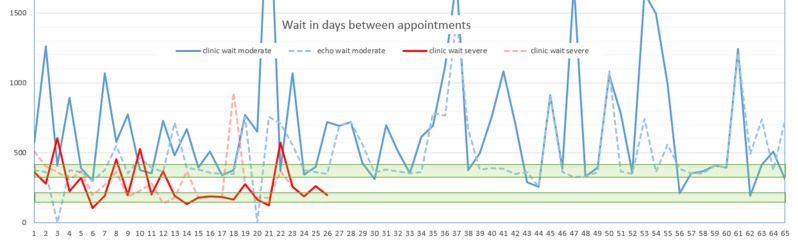
Line graph showing the wait in days to between clinic and echo appointments by valve disease severity

**Conclusion** This study highlights the potential for this valve clinic to improve the timing and coordination of clinical and echocardiographic local valve surveillance. It is likely this will reduce the considerable variability in the timing of echo and follow-up as well as freeing up consultant clinic slots. Longer term data will allow evaluation of clinical outcomes and the timing of follow-up and interventions with reference to a standard care group in other clinics. Patient and stakeholder feedback and economical and sustainability assessments could also be assessed in future studies.

**References**

1. Bennett S, et al. Clinical indications and triaging for adult transthoracic echocardiography: a consensus statement by the British Society of Echocardiography in collaboration with British Heart Valve Society. Echo Res Pract. 2022;9(1):5.
2. d’Arcy JP, Chambers BJ, Ray S, Bridgewater B Valvular heart disease: the next cardiac epidemic. Heart. 2011;97:91-93.
3. Ionescu A, et al. Are valve clinics a sound investment for the health service? A cost-effectiveness model and an automated tool for cost estimation. Open Heart. 2015;2(1):e000275.
4. Vahanian A, et al. 2021 ESC/EACTS Guidelines for the management of valvular heart disease. Eur Heart J. 2021.

## ABS059 Much echo about nothing? A retrospective audit of aortic valve calcification and aortic stenosis in Cornish lung cancer screening patients

Cara Campbell^1^, Matthew Berry ^1^, Mohammed Abubakr ^2^

^1^Respiratory Medicine Department, Royal Cornwall Hospital, Truro, UK, ^2^Cardiology Department, Royal Cornwall Hospital, Truro, UK

*Echo Research & Practice 2026*, **13(Suppl 1):**ABS059

**Introduction** Lung cancer screening identifies a multitude of incidental findings requiring further investigation. As per the national lung cancer screening protocol, visual moderate to severe aortic valve calcification (AVC) on low dose CT (LDCT) requires evaluation with echocardiography for aortic stenosis.

**Objective** To review the outcomes of screening patients with AVC referred for echocardiograms and the use of AVC as a predictor for stenosis in a local screening population.

**Methods** The study included all lung cancer screening patients with moderate to severe AVC on their LDCT scans from November 2023 to May 2025 in Cornwall. Severity of aortic stenosis was defined by the AV Max and valve area as per the British Society of Echocardiography (BSE) triage of echocardiography.

**Results** A total of 11,085 lung cancer screening LDCT scans were performed. The incidence of moderate to severe AVC was 1.21% with 4 (0.036%) patients referred for TAVI. Echocardiograms were available for 120/134 (89.6%) of patients. Of the 42/120 (35.0%) patients with severe AVC on CT, only 2/42 (4.76%) had severe aortic stenosis on echocardiogram requiring TAVI. 25/42 (59.5%) had mild to moderate aortic stenosis. The remaining 15/42 (35.7%) with severe AVC had normal aortic valves with no evidence of stenosis. From the moderate AVC on CT scan group, 2/78 (2.56%) had severe aortic stenosis, requiring TAVI. An increased proportion had no aortic stenosis, with 56/78 (71.79%) having normal aortic valves on echocardiogram. An inter-related reliability analysis was performed using Cohen’s Kappa. There was only slight agreement (k = 0.05) between severe AVC and severe aortic stenosis. Severe AVC was a better predictor for aortic stenosis (k = 0.65) than moderate AVC (k = 0.23).

**Fig. 1(abstract ABS059):**
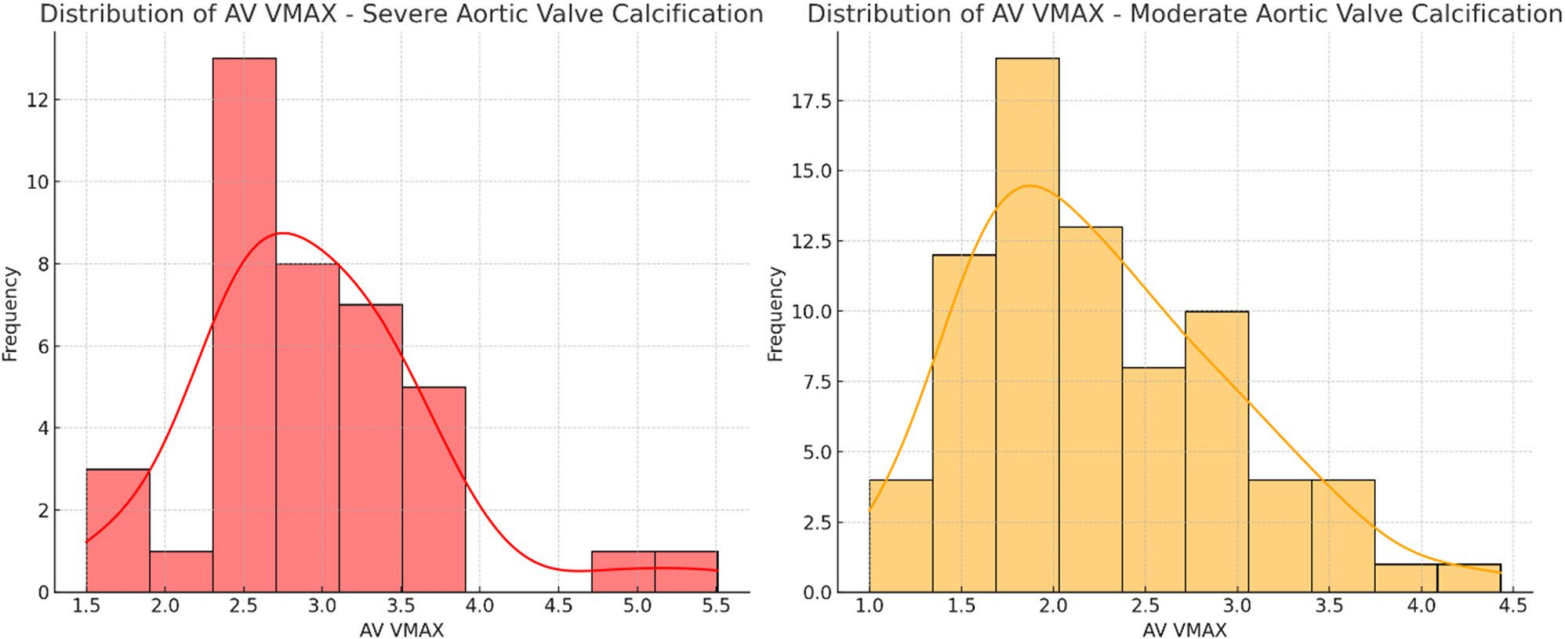
Distribution of AV VMAX for patients with severe and moderate aortic valve calcification

**Conclusion** In our population AVC correlates poorly with aortic stenosis for patients with moderate AVC. Severity of AVC is weakly correlated with increased valvular velocities. Investigating patients with AVC for aortic stenosis requires a significant amount of time and resources, and further research is needed to confirm the utility of following up these incidental findings.

